# Insights into the Protonation State and Counterion Interactions of Linear Polyethyleneimine Materials

**DOI:** 10.3390/gels12100912

**Published:** 2026-10-10

**Authors:** Agustín J. Byrne, Daniel Vladimir, Yamila Garro-Linck, Guillermo J. Copello, Ayelén F. Crespi, Daniel Vega, Mariela Bollini, Paula G. Franco, Juan M. Lázaro-Martínez

**Affiliations:** 1 Universidad de Buenos Aires, Facultad de Farmacia y Bioquímica, Ciudad Autónoma de Buenos Aires 1113, Argentina; abyrne@docente.ffyb.uba.ar (A.J.B.); daniellaura@ffyb.uba.ar (D.V.); gcopello@ffyb.uba.ar (G.J.C.); acrespi@docente.ffyb.uba.ar (A.F.C.); pgfranco@ffyb.uba.ar (P.G.F.); 2 CONICET—Universidad de Buenos Aires, Instituto de Química y Fisicoquímica Biológicas (IQUIFIB), Ciudad Autónoma de Buenos Aires 1113, Argentina; 3 ONICET—Universidad de Buenos Aires, Instituto de Química y Metabolismo del Fármaco (IQUIMEFA-UBA-CONICET), Ciudad Autónoma de Buenos Aires 1113, Argentina; 4 Centro de Investigaciones en Bionanociencias (CIBION – CONICET), Polo Científico Tecnológico, Ciudad Autónoma de Buenos Aires 1425, Argentina; mariebollini@gmail.com; 5 Universidad Nacional de Córdoba, Facultad de Matemática, Astronomía, Física y Computación, Córdoba 5000, Argentina; y.garrolinck@unc.edu.ar; 6 Consejo Nacional de Investigaciones Científicas y Técnicas (CONICET), Instituto de Física Enrique Gaviola (IFEG), Córdoba 5000, Argentina; 7 Comisión Nacional de Energía Atómica, San Martín, Buenos Aires 1650, Argentina; danielvega@cnea.gob.ar

**Keywords:** polyethyleneimine, protonation, crystallinity, *solid-state* NMR, XRD, SAXS, theoretical calculations, TD-NMR, *gel-state* NMR, HRMAS NMR

## Abstract

Polyethyleneimine (PEI) polymers are widely used in the synthesis of nanoparticles, hydrogels and composite materials for biotechnological and environmental applications. However, the solid-state structural organization of linear PEI (L-PEI) materials, and how it is influenced by molecular weight, protonation state and counterion type, have not yet been comprehensively studied. In this work, *solid-state* nuclear magnetic resonance (*ss*-NMR), X-ray powder diffraction (XRD), small-angle X-ray scattering (SAXS) and time-domain NMR (TD-NMR) experiments were combined with theoretical calculations to analyze the local chemical environments, crystalline and nanoscale organization, and molecular mobility of L-PEI materials with different molecular weights. The analyzed materials were the free base (PEI·FB), the hydrochloride (PEI·HCl), the trifluoroacetate (PEI·TFA), and the sulfate-containing forms of L-PEI (PEI·Na_2_SO_4_ and PEI·H_2_SO_4_), as well as an intermediate protonation state obtained by the heterogeneous treatment with HCl-saturated isopropanol. Proton, ^13^C or ^15^N *ss*-NMR studies evidenced different chemical environments associated with changes in protonation state and counterion identity. Furthermore, XRD and SAXS showed that these variables, together with molecular weight and sample preparation conditions, influenced the crystalline arrangement and characteristic nanoscale organization of the polymer chains. Moreover, TD-NMR revealed the presence of three dynamically distinct proton populations, assigned to rigid, interfacial, and mobile domains, whose relative contributions and relaxation behavior varied among the PEI materials. Finally, high-resolution magic-angle spinning (HRMAS) NMR experiments were used to study different PEI-gel materials and films as well as the crosslinking of protonated PEI and sulfate ions.

## 1. Introduction

Interest in PEI polymers has increased significantly in recent years in fields such as bioengineering and nanotechnology, mainly because of its structure and high charge density, which favor interactions with various charged biomolecules [1,2,3]. These characteristics have enabled applications ranging from antimicrobial strategies to environmental remediation. Moreover, the physicochemical properties of PEI materials are ideal for the design and development of nanoparticles intended for the encapsulation of genetic material [4,5,6]. Recent studies have demonstrated the capacity of PEI polymers to improve the colloidal stability of nanoparticles and their interactions with biological membranes [7]. Studies focusing on the antibacterial capacity, conferred by the high charge density and the effects on certain bacteria, of different PEI materials are also noteworthy [8]. Beyond the biomedical field, PEI, used for membrane functionalization, has demonstrated high efficiency in wastewater decontamination [9] and the removal of heavy metals such as Pb(II), Fe(III), Cu(II), and Ni(II) [10].

Unlike its branched version (B-PEI), L-PEI contains exclusively secondary amines in its chain, allowing it to adopt a semicrystalline structure (Scheme 1) [11,12,13]. In contrast to the amorphous, semi-solid nature of B-PEI, the solid-state organization of L-PEI favors the formation of complexes with other biomolecules that exhibit greater stability and lower cytotoxicity than their branched counterparts [14]. These advantageous properties have prompted researchers to study the possible applications of L-PEI, for which efforts have focused on its structural characterization [2]. Although L-PEI is widely employed in gene delivery, nanomedicine and biomaterial applications, the relationship between its structural organization and functional performance remains poorly understood. Parameters such as protonation state, hydration, molecular weight and preparation conditions may influence not only the physicochemical properties of the polymer but also its interactions with nucleic acids and other biological systems. Therefore, establishing links between structural characterization and application-relevant properties could provide valuable information for the rational selection and optimization of PEI-based materials.

The semicrystalline nature directly influences fundamental properties such as complexing capacity, thermal stability, and behavior in different environments [5,6,7,12,15,16]. Among the available techniques, NMR stands out as an ideal tool for determining key parameters such as the degree of polymerization, purity, and functional group distribution [15,17,18]. However, the determination of these parameters mostly relies on *solution-state* NMR, which presents limitations when the polymer either has low solubility or forms crosslinked networks, as is the case of hydrogels. In these systems, the absence of molecular rotation and the low mobility of the polymer chains broaden the signals and reduce the spectral resolution degree [19,20]. To overcome the limitations of these conventional techniques, *ss*-NMR has become essential because it allows for the acquisition of atomic-level structural information from both insoluble materials [19,21,22] and ordered systems [23,24]. Finally, HRMAS NMR spectroscopy emerges as a tool capable of providing information about swollen gel systems with a resolution comparable to that of solution NMR [25,26,27], facilitating the characterization of the liquid–solid interface [28].

In the case of L-PEI, these techniques are extremely useful in providing a deeper characterization of its structural properties, which directly determine its performance in biotechnological applications [29]. While *ss*-NMR and HRMAS spectroscopies provide local, atomic-level information, XRD and SAXS complement this analysis by resolving longer-range chain organization. SAXS allows the study of semicrystalline and partially ordered systems [30], providing information of chain organization in the nanometer range such as interchain domain spacing, chain folding density and/or particle agglomerates [31,32,33]. Simultaneously, time-domain NMR and low-field experiments serve as tools to map the heterogeneity of the macromolecular network, enabling the quantification of dynamically distinct rigid, interfacial, and mobile polymer domains [34]. The combination of these scattering and relaxation techniques offers greater perspective on how structural order influences local chain mobility [35]. Moreover, the use of computational techniques complements the NMR studies by allowing the analysis of how protonation and chain packing independently affect the ^13^C chemical shifts [36].

The structural characterization presented in this work revealed that L-PEI cannot be considered a single, structurally invariant material. Instead, the protonation state, the hydration level, the molecular weight and the preparation conditions generate distinct supramolecular organizations and dynamic behaviors. Since PEI is widely employed in gene delivery, biomaterials and nanotechnology applications, understanding these structural features may be essential for predicting and optimizing its functional performance. In this work different spectroscopic techniques were used to characterize the changes in the protonation degree, as well as the effect of the counterion type (chloride, sulfate and trifluoroacetate) in different PEI structures in the solid state. In particular, the changes in the bulk chain mobility in PEI materials and the impact of the different synthesis-related treatments (lyophilization, crystallization, protonation and deuterium exchange) were analyzed by TD-NMR. Furthermore, novel materials consisting of TFA and Na_2_SO_4_ or H_2_SO_4_-containing linear PEI polymers are reported for the first time. Finally, the use of HRMAS NMR experiments were used to extend this structural and dynamic characterization to swollen PEI gels and PEI-containing films, revealing that the local chemical environments identified in the solid state persist upon swelling and are further modulated by ionic crosslinking with sulfate ions.

## 2. Results and Discussion

### 2.1. XRD and Solid-State NMR Studies in PEI Polymers

In recent years, the acidic hydrolysis of commercial poly(2-ethyl-2-oxazoline) polymers of different molecular weights has become a standard approach for the synthesis of L-PEI hydrochloride (PEI·HCl) materials [12,37]. These polymeric solid materials have been studied by *ss*-NMR using standard or filtered ^13^C CP-MAS experiments, which allowed the detection of differences in molecular order. Specifically, ^1^H dipolar or ^1^H spin-lock filters allowed the detection of the magnetization coming from either the amorphous (δ^13^C = 44.5 ppm) or the crystalline regions (δ^13^C = 42.9 ppm), respectively [12]. In the present work, linear PEI·HCl polymers were neutralized with a NaOH solution in order to yield the corresponding solid PEI free-base (PEI·FB) polymers. Even though the synthetic procedure is relatively simple, some precautions need to be taken into account in order to yield a particular crystalline phase in the samples. According to previous works of Chatani et al. done on crystallized samples of PEI·FB [38,39,40], the XRD profiles are particularly relevant to determine which of the four crystalline phases are present in the powder samples. This is because the water molecules induce phase transitions among the crystalline arrangements of PEI·FB, giving rise to four different structures depending on the ratio between the monomer and water molecules (-CH_2_CH_2_NH-:H_2_O): anhydrate (1:0), hemihydrate (1:0.5), sesquihydrate (1:1.5) and dihydrate (1:2). Within these phases, the polymer chains adopt the fully extended planar-zigzag form in the three types of hydrates, while adopting the double-stranded helical conformation in the anhydrate state due to the different hydrogen bonding in each crystal form. Interestingly, the XRD profiles of the different PEI·FB samples were completely different when the same experimental procedure was used (Figure 1). Moreover, the PEI·FB samples with molecular weights of 11 and 22 kDa presented dihydrate crystalline structures in contrast to the hemihydrate pattern observed for the 87 kDa PEI·FB sample. The 217 kDa PEI·FB sample did not fit in any particular crystalline model, being intermediate between the hemihydrate and dihydrate forms. Although crystalline profiles are known to vary with temperature and humidity [41], in this work we demonstrated that the crystalline arrangement was also strongly affected by the synthesis conditions used to obtain PEI·FB from the corresponding PEI·HCl forms. Interestingly, the use of water that was not freshly distilled during the neutralization process allowed the detection of additional XRD reflections consistent with the presence of a bicarbonate-containing phase or species co-crystallized with PEI·FB (Figure S1), whereas when freshly distilled water was employed in the preparation of working solutions, the cocrystallization of this chemical species was prevented. Moreover, the rapid addition of the NaOH solution resulted in an XRD profile corresponding to one water molecule per PEI monomer in the 22 and 87 kDa PEI·FB samples (Figure S1).

Different factors affected the diffraction patterns obtained by XRD. The protonation of amine groups (PEI·HCl) yields a completely different XRD spectrum compared to PEI·FB materials. However, among the PEI·HCl samples, different molecular weights do not result in differences in their XRD patterns as was previously shown [12]. Regarding the PEI·FB materials, hydration, and thus sample preparation, has a primary impact on the XRD spectra, with the hemihydrate, monohydrate, and dihydrate phases being obtained in this work (Figure 1 and Figure S1 and Table S1). For this reason, before performing the characterization techniques in all the samples, the XRD spectra were measured to be able to infer the present crystalline structure. In addition, the measurements were carried out within 4 h of their synthesis (after being dried) to avoid transformations by contact with humidity.

Regardless of the molecular weight, the *solution-state* NMR spectra of PEI·FB performed in deuterated methanol (CD_3_OD) were essentially identical, showing two [δ^1^H (ppm) = 2.74 (s, -CH_2_-CH_2_-NH-, main signal) and 3.40 (t, *J* = 7.1 Hz, -NH-CH_2_-CH_2_-OH, terminal group)] and four resonance signals [δ^13^C (ppm) = 41.7 (-NH-CH_3_, terminal group), 48.0 (-NH-CH_2_-CH_2_-OH, terminal group), 49.7 (-CH_2_-CH_2_-NH-, main signal) and 58.3 (-NH-CH_2_-CH_2_-OH)] in the ^1^H- and ^13^C-NMR spectra, respectively (Figure S2). These NMR results were relevant to study the terminal groups and the main monomeric structure of the PEI polymers. The terminal methyl group of the linear PEI polymer could not be resolved due to the overlapping of its signal with the main signal in the ^1^H-NMR spectrum; however, this signal was observed in the ^13^C-NMR spectrum at 41.7 ppm. Additionally, the reaction with carbon dioxide and the amine groups could be detected by the visualization of the carbamate groups at δ^13^C = 165.3 and 161.4 ppm (^−^O_2_C-^+^NH_2_-CH_2_-), which is relevant for materials intended for direct air capture and CO_2_ removal [42,43,44,45,46,47].

As for the ^13^C *ss*-NMR characterization, all the PEI·FB samples exhibited higher chemical shift values (δ^13^C ~48–54 ppm) in comparison with those corresponding to the PEI·HCl samples (δ^13^C ~38–47 ppm) (Figure 2). As in the hydrochloride samples, two ^13^C resonance signals were observed in the ^13^C CP-MAS spectra for the free bases. Previous works employing ^13^C-NMR edition experiments with ^1^H dipolar filter and ^1^H spin-lock filter allowed the assigning of each signal to differences in the molecular ordering in PEI·HCl materials [12]. In the case of PEI·FB, these experiments did not allow us to obtain the same information for each ^13^C signal. In particular, the 11 kDa PEI·FB sample displayed signals at slightly lower ^13^C chemical shift values (δ^13^C = 48.4 and 50.3 ppm) compared with the samples with other molecular weights. Considering the shorter polymer chain length in comparison with the other PEI samples, it is possible that a different degree of molecular confinement may take place; however, further studies are needed to support this possibility.

Another aspect analyzed by *ss*-NMR was the treatment of the PEI·FB samples with HCl-saturated isopropyl alcohol (IPA·HCl) to precipitate the corresponding PEI·HCl samples. Upon addition of IPA·HCl to a methanolic PEI·FB solution, the PEI structure showed a ^13^C spectrum compatible with complete protonation, displaying only one signal at 42.9 ppm (PEI·HCl, Figure 3C). In contrast, the addition of IPA·HCl to the solid PEI·FB sample resulted in an intermediate protonation state for the PEI structure (PEI-IPA·HCl, Figure 3D). Thus, this heterogeneously protonated form showed at least four ^13^C resonance signals at 51, 48, 44 and 43 ppm. The signals at 51.8/51.1 and 43.7/43.0 ppm were tentatively assigned to the methylene carbons bonded to non-protonated and protonated nitrogen atoms, respectively. For the signals at 47.8 and 47.0 ppm, the protonation degree varied along with the degree of confinement of the polymeric regions into crystalline or amorphous domains, as evidenced by the chemical shift values obtained for PEI-IPA·HCl (Figure 3).

The addition of TFA, in turn, yielded the corresponding PEI trifluoroacetate (PEI·TFA). The ATR-FTIR spectrum for the PEI·TFA sample (Figure S3) shows the ionic interaction between the trifluoroacetate and protonated amine groups due to the characteristic signals from the asymmetric (ν*_as_* = 1672 cm^−1^) and symmetric (ν*_sym_* = 1440 cm^−1^) stretching modes of the carboxylate groups (RCO_2_^−^) in comparison with the stretching mode of the carbonyl group of the carboxylic acid (RCO_2_H) in the trifluoroacetic acid present at ~1783 cm^−1^ [48,49,50]. Furthermore, the asymmetric stretching of the C-F bond is present at 1196 cm^−1^. Additionally, the protonated PEI structure presents an intense absorption bands associated with its strong intermolecular hydrogen-bonding network around 2900–2300 cm^−1^. Moreover, the ^13^C *ss*-NMR result shows values at 43.3 (-CH_2_-^+^NH_2_-) and 117 (-CF_3_)/163.3 (-CO_2_^−^) ppm associated with the PEI and TFA moieties, respectively (Figure 4). In particular, the -CF_3_ signal appeared as a broad resonance peak at 117 ppm. This broadening arose from both the coupling between ^13^C and ^19^F nuclei and the low efficiency during the ^1^H → ^13^C cross-polarization step in the ^13^C CP-MAS acquisition. Furthermore, the XRD profile of PEI·TFA was markedly different from those of the PEI·FB samples, indicating a different crystalline arrangement together with an amorphous background contribution (Figure 4). To the best of our knowledge, this structural organization has not been previously described for linear PEI·TFA (Figure 4). The diffraction pattern for the PEI·TFA sample revealed the coexistence of ordered and disordered regions, as evidenced by the intense reflections at 2θ = 19.5 and 20.5°, as well as the broad amorphous background extending throughout the entire spectrum. The presence of sharp and well-defined peaks suggested that the interaction between PEI and TFA promotes a high degree of short-range ordering within the material. As with the hydrochloride salt, the protonation of the PEI·FB amine groups by TFA gave rise to ionic pairs (PEI·TFA). In this structure, the trifluoroacetate anion provided a sterically demanding and highly electronegative environment that might influence chain packing and intermolecular interactions. Previous studies on TFA-containing organic salts have shown that this anion can promote strong hydrogen bonding interactions while simultaneously inducing segregation of fluorinated domains [24,51,52]. The peaks observed at 2θ = 30.1 and 32.4° are unusual for semicrystalline PEI materials [12,35,40] and may be tentatively associated with short-range packing involving trifluoroacetate groups and dense arrangements of fluorine and oxygen-rich domains. However, additional structural studies are required to confirm these assignments.

Furthermore, the introduction of other counterions results in dramatic changes to the XRD profiles, as seen when switching from chloride to TFA or sulfate ions (Figure 4 and Figure S4). In particular, the PEI·sulfate samples, where protonated PEI chains were crosslinked with sulfate ions, had previously been reported using copper sulfate [12]. In this work the PEI·H_2_SO_4_ and PEI·Na_2_SO_4_ samples were synthesized from aqueous solutions of PEI·HCl and sulfuric acid or sodium sulfate, respectively. The physicochemical characterization of these materials was practically identical among all samples (Figures S4 and S12). Particularly, the PEI·sulfate materials are of interest for industrial application. In this regard, Yang et al. reported the combination of PEI with ammonium sulfate as a composite leaching agent for mining [53].

Additionally, the ^1^H *ss*-NMR spectra at a MAS rate of 60 kHz of the different PEI materials showed well-resolved signals (Figure 5). These signals corresponded to the hydrocarbon chain (δ^1^H = 2–4 ppm), the protonated amine groups (-^+^NH_2_-, δ^1^H > 9–10 ppm) and water molecules (δ^1^H = 5–7 ppm), including both free water and water that was hydrogen-bonded to the -NH- groups. Surprisingly, the δ^1^H values for the hydrocarbon skeleton of the PEI·HCl samples were greater than those of the PEI·FB materials, in contrast to the trend observed for the δ^13^C values. In the case of the PEI·HCl material, the crystallized sample showed slight differences when compared to the lyophilized sample. Particularly, the methylene moieties in the crystallized sample of PEI·HCl appeared at δ^1^H = 3.39 ppm, the region of water molecules appeared at δ^1^H = 5.37 ppm and the -^+^NH_2_- signal was observed at δ^1^H ~10 ppm (Figure 5). In the lyophilized PEI·HCl samples, methylene signals, water molecules and -^+^NH_2_- groups resonated at δ^1^H of 3.65, 6.05 and ~9.8 ppm, respectively (Figure 5). In particular, the broad -^+^NH_2_- signal was more defined in the case of the crystallized PEI·HCl in comparison with the lyophilized PEI·HCl. During the lyophilization process, the disorder of the hydrocarbon chain of PEI increased, giving rise to a more heterogeneous distribution of local environments consistent with the redistribution of the signal toward the interfacial component observed by TD-NMR which may explain the broader signal for the -^+^NH_2_- in comparison with the crystallized sample. Moreover, the ^1^H signal for water molecules was detected at different δ^1^H values, being higher for the lyophilized PEI·HCl sample. The different crystallized PEI·HCl polymers showed a similar behavior; however, the water populations were not observed in the ^1^H-MAS spectra. This might be due to the broader signal of the methylene segments and the stronger ^1^H-^1^H dipolar interactions, which likely overlapped with the water signal at δ^1^H = 3.66 ppm in the PEI polymers of other molecular weights. The PEI·FB materials only showed the ^1^H resonance signals associated with the methylene (δ^1^H = 2.58 ppm) and -NH-/water molecule moieties (δ^1^H = 5.04 ppm) (Figure 5).

Additionally, the PEI-IPA·HCl system, which consisted of a partially protonated structure as determined by the ^13^C *ss*-NMR studies (Figure 3D), showed a ^1^H-MAS spectrum that was quite similar to that of the PEI·FB polymer (Figure 5). No signal corresponding to the protonated form was detected at δ^1^H ~10 ppm. This observation may be associated with the rapid proton exchange between the small fraction of protonated nitrogen sites, resulting from the fast addition of IPA·HCl, and the water population at δ^1^H = 4.86 ppm. Alternatively, the crystalline signal of the PEI·FB might have masked the signals corresponding to the low content of PEI·HCl, preventing them from being detected in ^1^H-MAS RMN experiments.

In the different samples, the assignment of the ^1^H resonance signal of water molecules was based on the information obtained from the 2D ^1^H-^1^H SQ-DQ experiments (Figure 6). Using different rotor-synchronized times (1–4), it was demonstrated that the water signals (H_2_O in Figure 6) did not display a strong interaction with the PEI hydrocarbon structure in the different protonated materials (PEI·HCl or PEI·FB) neither in the crystallized nor in lyophilized PEI·HCl samples. This phenomenon is attributed to the mobile nature of these water molecules, as opposed to crystallographic or rigid water molecules [54].

Specifically, the PEI·TFA sample showed four ^1^H-NMR signals at δ^1^H = 2.88 (-CH_2_-), 6.94 (H_2_O), 9.82 (-^+^NH_2_-), and 12.24 (-^+^NH_2_-) in the solid state (Figure 5). These ^1^H-NMR patterns differed from those of the other PEI structures. As confirmed by the 2D ^1^H-^1^H SQ-DQ spectrum analyzed below (Figure 6D), at least two well-resolved resonance signals could be attributed to different protonated states of the nitrogen atoms in the PEI·TFA (signals a and a’ in Figure 6D). Although these resonance signals can only be attributed to the protonation of the amine groups by the action of the TFA molecules, the main hypothesis is that these protonated structures coexist in different segments during the crystallization of the polymeric chains. Interestingly, and unlike in the ^1^H-MAS (SQ) spectrum, in the 2D ^1^H-^1^H SQ-DQ spectrum obtained for the PEI·TFA sample, the water signal at δ^1^H = 6.94 ppm (“H_2_O” in Figure 6B) was absent, which is due to the sufficient rotational/translational dynamics that average out the dipolar couplings and hinder the detection of DQ coherences. However, the 2D ^1^H-^1^H SQ-DQ spectrum was particularly useful to demonstrate that both protonated structures (SQ = 12.2 and 9.8 ppm) are in close spatial proximity to each other, as evidenced by the off-diagonal signals at DQ = 22.0 ppm, along with the signal corresponding to the hydrocarbon chain of the PEI·TFA polymer (DQ = 13.8 ppm, Figure 6D).

Likewise, the ^15^N CP-MAS spectra were acquired at natural abundance for PEI·HCl, PEI·FB, PEI·TFA and PEI-IPA·HCl (Figure 7) and for the sulfate-containing PEI samples (Figure S4(IIC)). PEI·FB exhibited a single resonance signal at δ^15^N = −342.6 ppm, which was shifted upfield relative to PEI·HCl (δ^15^N = −334.9 ppm) [NO_PRINTED_FORM]. This upfield shift contrasts with the downfield shift observed for PEI·FB relative to PEI·HCl in the ^13^C CP-MAS spectra reported in this work (Figure 2 and Figure 7).

Particularly, the PEI·TFA, PEI·H_2_SO_4_ and PEI·Na_2_SO_4_ samples resonated at −345.1, −343.3 and −342.9 ppm, respectively. Thus, all three salt forms lie within 2.1 ppm of PEI·FB, whereas their ^13^C resonances show a consistent upfield displacement of approximately 6–8 ppm relative to the free base (Figure 3, Figure 4 and Figure S4). These observations show that, for the counterions studied here, the ^15^N chemical shift alone does not provide an unambiguous measure of protonation. The assignment of the protonated salt structures relies instead on the combined ^13^C- and ^1^H- *solid-state* NMR and FTIR experiments (described in the Supplementary Material Section).

PEI-IPA·HCl showed two resolved resonances at −341.9 and −346.6 ppm (Figure 7), demonstrating distinct local nitrogen environments, in agreement with the heterogeneity inferred from its ^13^C CP-MAS spectrum (Figure 3D). Their proximity to the free-base resonance does not, by itself, identify their protonation states or quantify the corresponding populations. These results support the coexistence of nitrogen sites with different protonation states and local structural organizations, giving rise to multiple ^15^N resonance signals. Previous studies conducted on lyophilized PEI·HCl allowed the identification of crystalline and amorphous domains with resonances at δ^15^N = −334.9 and −333.4 ppm, respectively [12], demonstrating that the differences in molecular order may also contribute to the resolution of distinct nitrogen environments.

### 2.2. DFT Calculations

To assess whether the differences observed in the ^13^C CP-MAS spectra arose from the protonation degree or from chain packing, theoretical calculations were carried out. In addition to the standard functional, dispersion-corrected calculations were performed to evaluate their influence on the results. The inclusion of dispersion improved the agreement with the experimental values for the fully protonated systems, but did not alter the relative trends on which the following analysis is based; the values obtained at the different levels of theory are detailed in Tables S2–S7. In the calculated models, the protonation of the internal secondary amines shifted the resonance of the neighboring carbons upfield (Table 1). The six internal backbone carbons (C3–C8, Figure 8A), which are flanked on both sides by protonatable secondary amines, exhibited an average calculated chemical shift of 57.2 ppm in the unprotonated system (with 0% protonation corresponding to the PEI·FB). Upon full protonation, the average calculated chemical shift decreased to 47.9 ppm for the chloride-containing model (Figure 8B) and to 45.7 ppm for the TFA-containing model (Figure 8C), corresponding to changes of approximately 9.3 and 11.5 ppm, respectively. For both counterions, the calculated results were compared with experimental data using the same two metrics: the absolute chemical shift of the fully protonated form, and the protonation-induced shift Δδ relative to the free base (Table 1). For the absolute shift, the calculated values (47.9 ppm for chloride and 45.7 ppm for TFA) reproduced the experimental values (42.9 and 43.3 ppm, respectively). For the protonation-induced shift, the calculated Δδ was 9.3 ppm for chloride and 11.5 ppm for TFA, compared with experimental values of approximately 7.9 and 7.5 ppm; the agreement was therefore good for chloride but the shift was overestimated for TFA.

The interchain packing, defined here as the effect of stacking two chains held at the same extended conformation, had a much smaller effect on the ^13^C chemical shifts. Upon comparing the isolated hexamer with the dimeric model at the same protonation degree, it was found that the center of the backbone resonances changed by only 0.5 ppm in the free base (57.2 to 56.7 ppm) and by 0.6 ppm in the fully protonated system (47.9 to 48.5 ppm). In these models, and at constant chain conformation, the contribution of interchain packing to the ^13^C chemical shifts was roughly one order of magnitude smaller than that of protonation.

The chain conformation, however, was not independent of the counterion. In the dimeric models with chloride, the two chains are extended and strictly equivalent in the unprotonated system, whereas upon full protonation, the chains folding and this equivalence is lost, with differences of up to 4 ppm between formally equivalent carbons (Figure 8D,E). A comparable folding is observed in the fully protonated hexamer containing chloride, but not in the corresponding hexamer containing TFA, where the chain remains unfolded (Figures S5 and S6). This finding is consistent with the small, compactly charged chloride ion, which can bridge adjacent ammonium groups, thus favoring the generation of folded conformations. Conversely, the bulkier TFA moiety hinders such compact arrangement.

The steric role of the counterion is further supported by the arrangement it can adopt along the chain. Placing the chloride ions on the same side of the backbone (*cis*) rather than on opposite sides (*trans*) leads to the generation of a slightly less unfolded conformation, but leaves the calculated ^13^C shifts essentially unchanged, indicating that the results are robust with respect to the specific placement of the counterions. The equivalent *cis* arrangement could not be stabilized by TFA since during optimization, spontaneous proton transfer from the ammonium groups to the carboxylates occurred, with the resulting neutral acid molecules being reoriented towards the *trans*-like disposition. Conversely, the corresponding *trans* arrangement, optimized under identical conditions, retained the ion pair. Minor conformational adjustments therefore left the ^13^C shifts unaffected, whereas the pronounced folding described above induced a substantial change in the chemical shift.

The interchain packing was therefore modeled only with chloride, as the steric requirements of TFA made a comparably compact arrangement unlikely. This conformational difference contributed to the ^13^C shifts through additional through-space interactions, constituting a third variable, which is different from interchain packing as defined above, which modulated the resonances of the backbone carbons.

Protonation and packing differ not only in the chemical shifts they produce, but also in their dispersion among the individual backbone carbons. Full protonation causes internal carbons to resonate within a narrow frequency range (1.7–4.3 ppm, depending on the counterion), yielding a single, broad signal. Partial protonation, in contrast, can generate markedly inequivalent environments, with dispersions of up to 7.8 ppm, depending on how the protonated sites are distributed along the chain. The multiple backbone resonances observed for the PEI-IPA·HCl sample, spanning ≈ 8 ppm, are therefore likely to reflect a combination of two distinct contributions. On the one hand, the heterogeneous distribution of protonation environments, which alone accounts for dispersions of up to 7.8 ppm; on the other, the counterion-induced folding described above, which can add up to 4 ppm. Pure interchain packing, accounting for less than 1 ppm, is by contrast a minor contribution.

The correlation between calculated dispersion and protonation state is consistent with the number of signals observed experimentally. The unprotonated free base and the fully protonated forms, which show the smallest calculated dispersions, correspond to the samples displaying essentially a single backbone resonance. The intermediate protonation states show the largest calculated dispersions, in line with the partially protonated PEI-IPA·HCl sample, where multiple backbone resonances are observed. The origin is not strictly identical in the two cases. In a single model chain, the dispersion arises from inequivalent sites along the chain, whereas in the real material it reflects the coexistence of domains with different protonation degrees. However, in both cases, the same underlying cause applies: a heterogeneous distribution of protonation gives rise to multiple, inequivalent carbon environments.

The GIAO calculations were also extended to ^15^N nuclei of the hexamer and dimer models, at the same protonation degrees and levels of theory used for ^13^C (Tables S8–S10). For the unprotonated and fully protonated systems, where the four internal nitrogen atoms are chemically equivalent, the calculated shift reproduced experimentally observed downfield displacement upon protonation for chloride. For the partially protonated models (25% and 50%), each internal nitrogen atom was close to either the free-base or the fully protonated value rather than to an intermediate one. This bimodal behavior, consistent with the two resolved ^15^N signals observed for the partially protonated PEI-IPA·HCl sample, further supports the coexistence of nitrogen sites in discrete protonation states rather than a continuum of intermediate environments along a single chain.

In contrast to the ^13^C chemical shifts where interchain packing contributed less than 1 ppm, the dimeric chloride models showed a substantially larger packing-induced dispersion among nominally equivalent atoms (6 to 8 ppm for the free base, 16 to 17 ppm for the fully protonated form). This is consistent with the higher intrinsic sensitivity of ^15^N, relative to ^13^C, to the hydrogen-bonding and packing environment [55]. Experimentally, the trifluoroacetate and sulfate resonances are all close to that of PEI·FB, whereas the PEI·HCl value differs appreciably (Figure 7 and Figure S4). Steric or conformational arguments specific to trifluoroacetate cannot be extended to the sulfate observations, since sulfate is a small, compactly charged anion that acts as a bidentate bridge between protonated chains. Differences in ion pairing, hydrogen bonding and local solid-state organization are possible contributors, as the ion-pair and hydrogen-bond structures of alkylammonium salts are strongly anion-dependent [56]. It is worth mentioninig that no sulfate model was calculated, and gas-phase models used here do not reproduce the complete experimental counterion dependence.

### 2.3. Molecular Dynamics in PEI Polymers

Dipolar refocusing experiments, such as the magic sandwich echo (MSE) *ss*-^1^H NMR, are widely used in polymer and material characterization [57,58,59,60,61,62]. Specifically, this sequence employs a pulse train that reverses dipolar interaction, thereby preventing the signal loss from the more crystalline or rigid domains during acquisition, a common issue caused by instrument dead time.

Beyond the structural and protonation-state differences described above, low-field time-domain NMR experiments using the MSE sequence were performed to further investigate proton mobility and dynamic heterogeneity in the different PEI materials. The MSE-FID analysis revealed the coexistence of three dynamically distinct proton populations, which can be assigned to rigid (associated with crystalline regions), interfacial, and mobile domains (associated with amorphous regions) (Figure 9 and Table 2). The domains reported in Table 2 and Figure 9 correspond to single measurements per material. Error bars represent the standard error of each fitted amplitude coefficient, reflecting the precision of the fit given the model and the signal-to-noise ratio of each individual decay curve; as only one measurement was performed per sample, these do not capture inter-preparation reproducibility, which is acknowledged as a limitation of the present study. The quantitative analysis of the samples revealed a matrix that was structurally dominated by rigidity, where the combined rigid and interfacial fraction accounted for approximately 60% to 83% of the total proton signal in the three-component samples; for the two-component sulfate-containing samples, the rigid fraction alone accounted for 70–72% (Table 2), indicating that most protons experienced strongly constrained environments. The high proportion of constrained protons observed in both PEI·FB and PEI·HCl samples was consistent with a dense intermolecular packing and a highly interconnected hydrogen-bonding network, resulting in strong effective ^1^H-^1^H dipolar couplings (Table 2). Lyophilization and crystallization of PEI·HCl materials further modified the relative proportions of the rigid, interfacial, and mobile populations, reflecting a protonation-dependent reorganization rather than a uniform increase in matrix rigidity.

**Table 2:** Estimation of the rigid, interfacial and mobile domains for the indicated PEI polymer materials using TD-NMR (MSE), obtained from fitting Equation (S2) (see Supplementary Information). Uncertainties are propagated from the standard errors of the fitted amplitude coefficients.

Material	Rigid (%)	Interfacial (%)	Mobile (%)
11 kDa PEI·FB	55 ± 5	16 ± 6	30 ± 3
22 kDa PEI·FB	51 ± 4	23 ± 5	26 ± 3
87 kDa PEI·FB	55 ± 5	17 ± 4	28 ± 5
217 kDa PEI·FB	57 ± 5	18 ± 6	25 ± 3
11 kDa PEI·HCl	53 ± 3	29 ± 4	18 ± 1
22 kDa PEI·HCl	54 ± 2	21 ± 2	25 ± 1
87 kDa PEI·HCl	47 ± 2	24 ± 2	29 ± 2
217 kDa PEI·HCl	50 ± 3	27 ± 3	23 ± 2
87 kDa Deuterated PEI·HCl	49 ± 6	34 ± 6	17 ± 2
87 kDa Lyophilized PEI·HCl	40 ± 4	41 ± 4	19 ± 2
87 kDa PEI·TFA	31 ± 2	29 ± 2	40 ± 1
87 kDa PEI·Na_2_SO_4_^(1)^	72 ± 1	—	28 ± 1
87 kDa PEI·H_2_SO_4_ ^(1)^	70 ± 2	—	30 ± 2

^(1)^ Two-component model (Abragam + 1 exponential); interfacial and mobile fractions were not resolved as separate populations for this sample and are reported as a single combined mobile-like fraction (Mobile column).

The Abragam function parameters [63] corresponding to the rigid component (T_a_, w; Table 3) remained essentially unchanged with molecular weight and protonation state (a = 18–23 µs, b = 135–168 kHz across PEI·FB, PEI·HCl, PEI·Na_2_SO_4_ and PEI·H_2_SO_4_ samples), indicating a similar local dipolar coupling network within rigid domains for these materials. This suggests that, despite variations in rigidity, the local organization and dynamic characteristics of the rigid domains was similar in all samples. In contrast, the interfacial component showed an increase in the spin–spin relaxation time (*T_2_*) in PEI·HCl samples, indicating a change in segmental mobility in regions connecting the more rigid and mobile domains. The most significant effect was observed in the mobile fraction. While the PEI·FB samples exhibited relatively short *T_2_* values (~0.07–0.1 ms), the PEI·HCl systems showed an increase in these values, reaching up to ~1.3 ms. This increase in *T_2_* values was consistent with weaker effective ^1^H-^1^H dipolar couplings and enhanced motional averaging in the non-rigid domains. This behavior may reflect reduced local constraints and/or changes in hydration and chain packing.

The lyophilized PEI·HCl sample showed a different behavior: even when the rigid component fraction was reduced, the increase in *T*_2_ values was less significant when compared to the crystallized PEI·HCl sample (Table 2 and Table 3). This finding indicates that the decrease in the rigid fraction did not directly translate into enhanced mobility, evidencing a decoupling between structural order and molecular dynamics. Considering the mobility regimes exhibited by the PEI·HCl and PEI·FB samples, it was observed that freeze-drying affected both the mobile and rigid domains (Table 2 and Table 3), resulting in an increase in the interfacial region for PEI·HCl. In this regard, the precipitation/crystallization of PEI·HCl and PEI·FB polymers during the synthetic procedures appears to provide a controlled environment for PEI chain ordering. Conversely, freezing PEI·HCl solutions and subsequently sublimating the water molecules induces stress in the material, leading to a tendency toward a certain degree of ordering, as evidenced by interfacial and rigid fractions of 41% and 40%, respectively (Table 2). Relative to the non-deuterated 87 kDa PEI·HCl sample, deuteration was accompanied by a marked decrease in the mobile-component *T*_2_ (1.338 to 0.381 ms) and a smaller decrease in the interfacial *T*_2_ (0.032 to 0.024 ms), reflecting the change in the observed proton population and dipolar couplings upon deuteration, rather than a moderate increase in either parameter. Overall, these results demonstrate that molecular mobility in these systems was not solely governed by the number of rigid domains. Instead, specific interactions arising from different protonation states played a dominant role in modulating polymer dynamics, particularly in the mobile and interfacial regions (Figure 9).

In contrast, the PEI·TFA polymer exhibited the most significant changes among the polymers treated with monodentate anions, with a decrease from approximately 24 to 16 percentage points in the rigid fractions, as compared to PEI·FB and PEI·HCl, respectively (Table 2). In addition, the PEI·TFA samples shows markedly higher values of the Abragam function parameters (a = 27.7 ± 4.1 µs, b = 203.4 ± 6.2 kHz) than the other materials (Table 3), suggesting that the trifluoroacetate anion locally altered the packing/dipolar coupling network of the rigid domains—consistent with the pronounced disorder evidenced by XRD experiments (Figure 4).

However, the sulfate-containing materials showed the greatest impact on the mobility of the PEI chains, with rigid proton fractions of 72% for PEI·Na_2_SO_4_ and 70% for PEI·H_2_SO_4_ (Table 2). Their bulk S:N ratios of approximately 0.49, together with the enrichment relative to the sulfate added (Supplementary Materials), provide independent compositional support for the formation of sulfate-rich ionic associations in the isolated materials. Combined with the relaxation behavior, these observations are consistent with ionic crosslinking by the bidentate sulfate anion restricting PEI segmental motion. However, near-stoichiometric charge compensation does not establish that every sulfate ion forms an interchain bridge, and the TD-NMR rigid fraction should not be interpreted as a percentage of crosslinked repeat units.

### 2.4. SAXS Studies in PEI Materials

The chain long-range ordering of PEI·FB and PEI·HCl dry samples was assessed by SAXS (Figure 10). Two common features were observed in all samples. These features were the presence of a steep slope at the low *q* regime and a correlation peak at the high *q* range. Therefore, in order to model the SAXS profiles, two equations were combined. For the low *q* regime, a power-law model was chosen since the profiles follow a characteristic scaling behavior. For the correlation peak, due to peak shape, a Lorentzian peak fitting model was chosen. The adjustment parameters are shown in Table 4. First, at low *q*, the steep slope represented by the power-law exponent α varied between 3.5 and 4, which is related to the fractal dimension, D (Table 4 and Table S11). For mass fractals regimes, α = Dm, varying between 1 and 3. For surface fractals, α is equal to 6−Ds and varies between 3 and 4, giving values for Ds between 3 for rough surfaces and 2 for smooth interfaces [64,65]. This implies that at the higher nanometer range, the nature of the scattering particles is related to surface fractals [66]. Second, at high *q*, a peak corresponding to a highly ordered structure was observed. This feature was observed between 0.08 and 0.1 Å^−1^ for most samples except for 22 and 217 kDa PEI·FB, which showed a subtle bump for which the maximum is not clear, probably indicating a low proportion of this structure in these materials. This peak corresponds to PEI long period with *d* = 2π/*q* in the range of 56–76 Å. A similar range for hydrated PEI samples was found by Hashida et al. [67]. This finding indicated that both free bases and hydrochlorides shared a similar nanoscale structure regarding their lamellar stacking and surface fractal structures. Nevertheless, in the case of the hydrochlorides, this peak appeared at a *q* value that was lower than that corresponding to the free bases. It can be assumed that the protonation state and/or counterion of PEI influences its nanoscale structure. This phenomenon was not evidenced when comparing the peak *q* value among PEI of different molecular weights. In the latter case, no trend could be observed.

The relevance of the protonation state and/or counterion effect was more evident when comparing SAXS profiles of the 87 kDa PEI polymer prepared under different experimental conditions (Figure 11). To carry out a proper comparison, these samples were also modeled using an equation that combines a power-law and a Lorentzian peak equation. From the comparison of the profiles, it was clearly observed that the influence of the counterions on the fractal dimension of the aggregates was negligible, as when molecular weights were compared, their α being between 3.5 and 4 values (Ds from 1.9 to 2.5) (Table 5 and Table S12). In contrast, the correlation peak *q* and the proportion of this structure in the bulk material were found to be highly affected by the different PEI treatments. Seemingly, the hydrochlorides presented the least prominent correlation peaks, either as HCl, lyophilized HCl (PEI·HCl-L), or obtained from IPA·HCl solutions.

In order to analyze the effect of counterions other than TFA, PEI·H_2_SO_4_ and PEI·Na_2_SO_4_ materials were synthesized from the corresponding PEI·HCl aqueous solution and the addition of sulfuric acid or sodium sulfate solutions, respectively (Figure 11 and Figure S8). Under these different preparation conditions, sulfate ions are expected to interact ionically with protonated PEI chains, potentially acting as ionic crosslinkers between polymer segments. Although the amount of sulfate added during the synthesis corresponds to only approximately 0.28 sulfate ions per PEI repeat unit, the isolated solids contain approximately 0.49 sulfate ions per repeat unit (Table S14). This enrichment was consistent with the selective precipitation of a sulfate-rich polymer fraction, so that the composition of the isolated materials differs from that of the initial reaction mixture. Interestingly, PEI·H_2_SO_4_ and PEI·Na_2_SO_4_ exhibited similar XRD patterns and ^1^H/^13^C *ss*-NMR spectra (Figure S4), suggesting the existence of comparable local chemical environments and a crystalline arrangement [12]. Remarkably, sulfate- and TFA-containing samples presented more intense peaks than PEI·HCl. These differences may arise from variations in the relative abundance of the correlated domains, their degree of organization, and the electron-density contrast introduced by the different counterions. In particular, PEI·TFA exhibited a broad and intense correlation peak that was shifted toward the lowest *q* and higher *d*. The assumption of the effect of the counterion was further supported by the observation that both samples obtained with sulfates showed two correlation peaks with different *d* corresponding to the first- and second-order reflections of a lamellar stacking (Figure 11).

### 2.5. HRMAS NMR Studies in PEI-Gel Materials

In general, NMR is the gold standard spectroscopic technique for characterizing a wide range of materials at the atomic scale. Regarding gel or soft materials, the HRMAS NMR methodology allows the study of these structures in the swollen state using deuterated solvents at very low MAS rates (4–5 kHz). In this sense, the experiments render solution-state-like NMR spectra similar to those observed for dissolved molecules. Complementing the solid-state characterization presented above, HRMAS NMR was used to examine how the local chemical environments of PEI persist and evolve upon swelling in gels and PEI-containing films. As a reference for signal assignment, the EGDE–IM gel (synthesized from ethylene glycol diglycidyl ether = EGDE and imidazole = IM) [22] model compound was analyzed first, followed by the PEI-EGDE gels, the starch-PEI films, and the effect of sulfate-induced crosslinking and swelling. Previously, we have reported the use of HRMAS NMR experiments to study the chemical structure of hydrogels from PEI·HCl polymers and the chemical crosslinking with EGDE due to their relevance in the adsorption of copper ions and the removal of emerging contaminants from water [25,26,27]. In this work, we will address the use of this technique to study the synthesis of these PEI hydrogel structures in acetonitrile, their swelling capacity in organic solvents and the inclusion of PEI polymers in starch films as well as the crosslinking with sodium sulfate.

First, the ^1^H HRMAS NMR spectrum is shown for the EGDE-IM hydrogel [22] swollen in D_2_O to indicate where the proton resonance signals corresponding to the crosslinking segments at δ^1^H = 3.2–4.5 ppm are observed (Figure 12). In addition, the signals associated with the mono and disubstituted imidazole rings and moieties of EGDE bounded to the imidazole ring are shown at δ^1^H = 6.5–8 and 4–4.5 ppm, respectively. When PEI-EGDE hydrogels are analyzed, the regions associated with the EGDE resonate around 3.25–4.20 ppm while the PEI segments are present at 2.25–3.25 ppm. The complete NMR analysis of the PEI-EGDE hydrogels synthesized in water was reported previously [25]. In this work, the PEI-EGDE gel was synthesized by replacing water with acetonitrile. In this regard, the ^1^H HRMAS NMR spectra for the PEI-EGDE gels synthesized in water or acetonitrile were similar, indicating comparable local chemical environments in both cases. Thus, chemical crosslinking can be carried out in both solvents, with water being more appropriate to reduce environmental impact during the synthetic procedure. Interestingly, the reaction time for the synthetic procedure was reduced to 40 min in acetonitrile, in contrast with 120 min in water. This can be associated with the higher solubility of EGDE in acetonitrile than in water. Moreover, the PEI-EGDE hydrogel shows an interesting swelling in CDCl_3_ (swelling capacity (S%) = 980 ± 67) which allowed its study by HRMAS NMR. Again, the ^1^H HRMAS NMR spectrum in CDCl_3_ was similar to those in D_2_O, with a slight decrease in the segment corresponding to the methylene moieties of the PEI structure (δ^1^H = 2.25–3.25 ppm). This effect can be attributed to the lower swelling of the protonated PEI segments within the gel when CDCl_3_ was used. However, the EGDE regions may contribute to the high swelling in the organic solvent.

Then, ^13^C HRMAS NMR experiments were performed in different PEI-related structures: starch-PEI film (10% PEI) [15] and PEI-EGDE gel synthesized in water (Figure 13) [25]. In both systems the protonated PEI moieties are shown around 43–44 ppm with the rest of the characteristic carbon resonance signals corresponding to the starch or crosslinker (EGDE) at chemical shift values higher than 60 ppm. Particularly, two different ^13^C environments can be observed at 43.3 and 43.7 ppm for the PEI segments in comparison with the starch-PEI film (δ^13^C PEI = 44.1 ppm), where the polymeric structure is dispersed in the starch structure and not covalently bounded. In the PEI-EGDE gel, the polymeric structure can be confined in different regions within the hydrogel as the result of the chemical crosslinking in the gel structure.

Finally, the ^13^C HRMAS NMR spectrum for the PEI-EGDE hydrogel swollen in 100 mM of Na_2_SO_4_ in D_2_O was studied (Figure 13). The spectrum shows a new ^13^C resonance signal at 46.6 ppm, consistent with an additional chemical environment arising from the ionic interaction between protonated PEI amine groups and sulfate ions. It is important to remark that the *ss*-NMR results did not detect differences in the ^13^C chemical shift values between PEI·H_2_SO_4_ and PEI·Na_2_SO_4_ in comparison with the PEI·HCl material (Figure S4). In the gel state, crosslinking with sulfate ions of PEI-EGDE gel may give rise to different environments where the carbons of the protonated PEI structures (δ^13^C = 43.3 and 43.7 ppm) were shifted to higher frequency values (δ^13^C = 46.6 ppm) due to the interaction with sulfate ions. Unfortunately, the PEI·H_2_SO_4_ and PEI·Na_2_SO_4_ materials did not present observable HRMAS NMR signals, tentatively attributed to their markedly lower swelling combined with insufficient segmental mobility to narrow the ^1^H linewidths under HRMAS conditions in comparison with PEI-EGDE hydrogels (S% = 4291 ± 281).

The swelling values for the PEI·H_2_SO_4_/PEI·Na_2_SO_4_ were 180 ± 4/185 ± 7, 174 ± 9/167 ± 3 and 136 ± 2/133 ± 3 at pH 4.0, 6.0, and 8.0, respectively. In particular, PEI·H_2_SO_4_ and PEI·Na_2_SO_4_ materials exhibited a decreasing trend in the swelling values with increasing pH. Values at pH 4 and 6 were quite similar, whereas the value at pH 8 was lower than that at pH 4. The decrease in the swelling properties with increasing pH is consistent with reduced protonation of PEI amine groups, whereas the higher positive charge density under acidic conditions may favor hydration and chain expansion, contributing to greater liquid retention.

## 3. Conclusions

In this work, we report a comparative solid-state characterization of linear PEI polymers with molecular weights ranging from 11 to 217 kDa in their free-base and protonated forms, including materials containing chloride, sulfate, and trifluoroacetate counterions, updating the state of the art in the solid state for L-PEI polymers. By combining *solid-state* NMR, XRD, SAXS, TD-NMR, and DFT calculations, this study provides a multiscale description of how molecular weight, protonation state, counterion type, and sample-processing conditions influence the local chemical environments, crystalline organization, nanoscale structure, and molecular mobility of L-PEI materials.

The experimental conditions used to prepare the PEI·FB samples strongly influenced the crystalline phases obtained from the corresponding PEI·HCl polymers. XRD allowed the identification of different hydrated crystalline arrangements in the PEI·FB samples, whereas their ^13^C CP-MAS spectra did not reveal significant differences among the different molecular weight or crystalline phases. These findings indicate that changes in long-range crystalline organization do not necessarily produce substantial differences in the local ^13^C chemical environments of the polymer backbone.

All materials were studied by *ss*-NMR experiments, focusing on the ^1^H, ^13^C, and ^15^N nuclei, which allowed us to characterize the changes produced according to the protonation state (free bases or hydrochlorides), as well as the effect of chloride, sulfate, and trifluoroacetate ions on the chemical shifts. DFT calculations supported the predominant influence of protonation on the calculated ^13^C chemical shifts and helped distinguish their contribution from those of chain conformation and interchain packing. In particular, the presence of proton signals between 5 and 7 ppm in the ^1^H-MAS spectra at a MAS rate of 60 kHz could be associated with non-structural mobile water molecules for PEI materials, as demonstrated by 2D ^1^H-^1^H SQ-DQ spectra, where no interactions were observed in the double coherences between these signals and those of the polymer. The novel PEI·TFA polymer exhibited an XRD spectrum with notable changes, as compared to that of other PEI materials. Likely, these changes were associated with the size of the trifluoroacetate anion, as well as different protonated populations of PEI chains associated with coexisting and interacting polymer regions in the solid, based on the SQ-DQ NMR results.

SAXS results complemented XRD findings, indicating that the characteristic organization at shorter nanometer length scales was more strongly influenced by counterion type and sample treatment than molecular weight. In contrast, the low-q behavior associated with larger polymer aggregates was broadly similar among the samples, suggesting that the large-scale aggregate morphology was predominantly governed by the polymeric framework and was less sensitive to the ionic composition.

TD-NMR revealed the coexistence of rigid, interfacial, and mobile proton populations in the PEI materials. Both the relative contributions of these domains and their relaxation behavior depended on protonation, counterion type, hydration, and preparation conditions. PEI·FB samples exhibited relatively short *T*_2_ values for the mobile fraction (~0.07–0.1 ms), whereas PEI·HCl showed values of up to ~1.3 ms, consistent with greater motional averaging in its non-rigid domains. Sulfate-containing materials exhibited more constrained polymer environments, consistent with ionic crosslinking between protonated PEI chains, whereas PEI·TFA showed a larger mobile fraction combined with a shorter mobile-component *T*_2_ than PEI·HCl.

Particularly, the use of HRMAS NMR experiments were useful to demonstrate both the incorporation of PEI in different types of hydrogels and films, as well as that the synthesis of PEI-EGDE gels can be done in acetonitrile or water. On the other hand, PEI-EGDE gels showed a high swelling in chloroform, which may allow chemical modifications to be made on the amino group for different applications. In addition, the ^13^C HRMAS NMR experiments showed the ionic interaction of PEI moieties with sulfate ions through the new resonance signal at 46.6 ppm as opposed to the protonated PEI regions at 43.4 and 43.7 ppm in PEI-EGDE gels.

Overall, this multitechnique characterization using *ss*-NMR, XRD, SAXS, TD-NMR, DFT and HRMAS NMR shows that the structural organization and molecular dynamics of L-PEI depend collectively on protonation state, counterion type, molecular weight, hydration and processing history, and that these solid-state features persist even in the swollen gels and films derived from PEI. This characterization provides a framework for the rational selection of L-PEI forms in the future development of nanoparticles, responsive gels, films, and gene-delivery systems.

## 4. Materials and Methods

### 4.1. Materials

Poly(2-ethyl-2-oxazoline) (PEOX) (molecular weights of 25, 50, 200 and 500 kDa), sodium hydroxide, hydrochloric acid, sulfuric acid, trifluoroacetic acid, hydrogen chloride solution (1.25 M in 2-propanol = IPA·HCl), sodium sulfate, anhydrous CaCl_2_ (≥96%), deuterium oxide (deuteration degree min. 99.9%), deuterated chloroform (99.8 atom % D) deuterated methanol (deuteration degree min. 99.8%) and anhydrous methanol (≥99.9%) were of analytic grade and used without further purification (Sigma-Aldrich, St. Louis, Missouri, USA). The PEI·HCl polymers with a molecular weight of 11, 22, 87, and 217 kDa as free bases were synthesized as described previously [12]. The EGDE-IM [22,68], PEI-EGDE [25] and starch-PEI [15] materials were described previously. In general, all experimental procedures and characterization techniques were performed at 24 ± 1 °C and relative humidity between 40 and 50%, unless otherwise specified.

### 4.2. Synthesis of PEI Materials

The different synthetic procedures of PEI materials are found in the Supplementary Content (SP).

### 4.3. Characterization Techniques

The different characterization techniques of PEI materials are found in the Supplementary Content (CT).

## Figures and Tables

**Figure 1:**
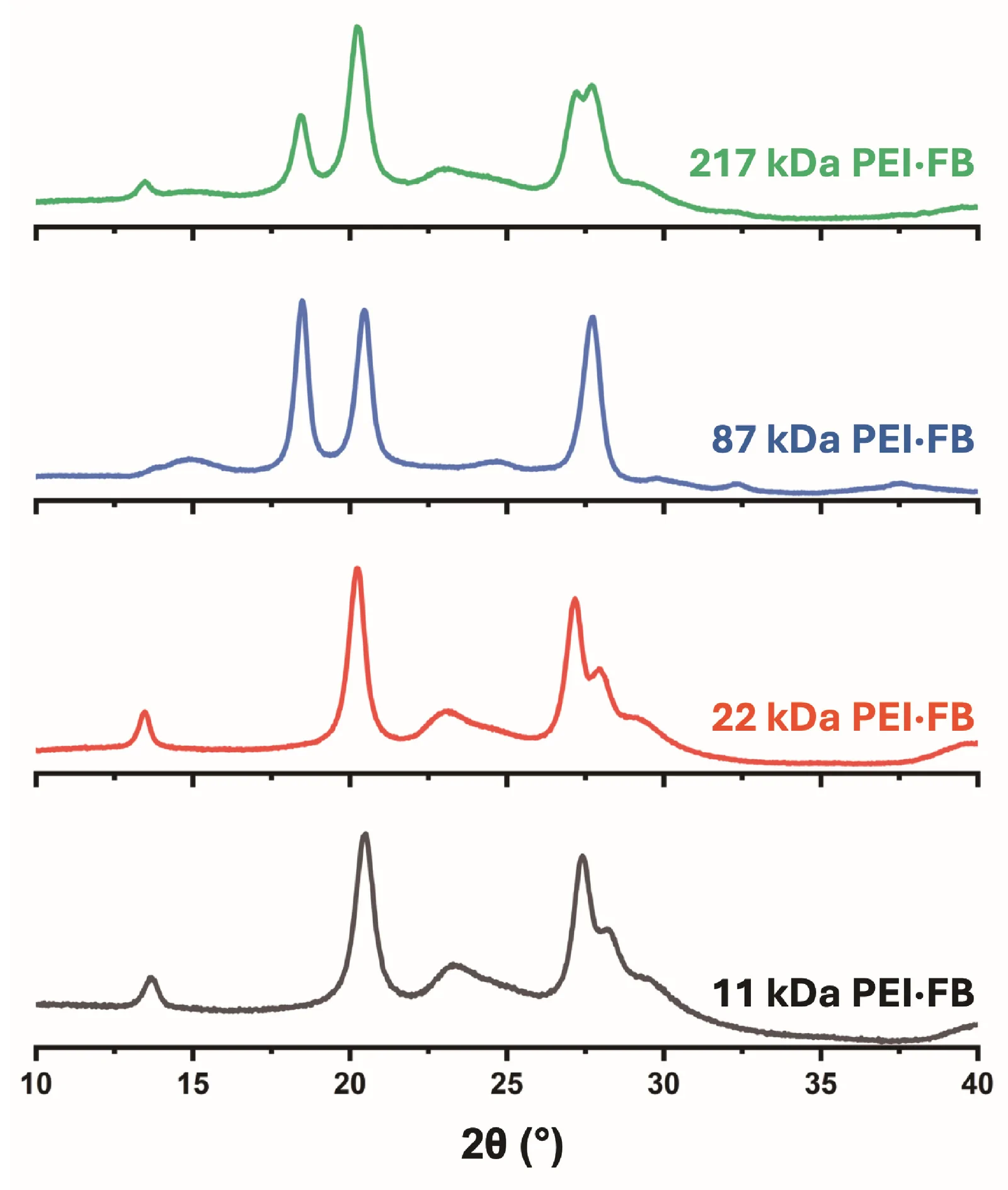
XRD spectra of PEI·FB samples with different molecular weights.

**Figure 2:**
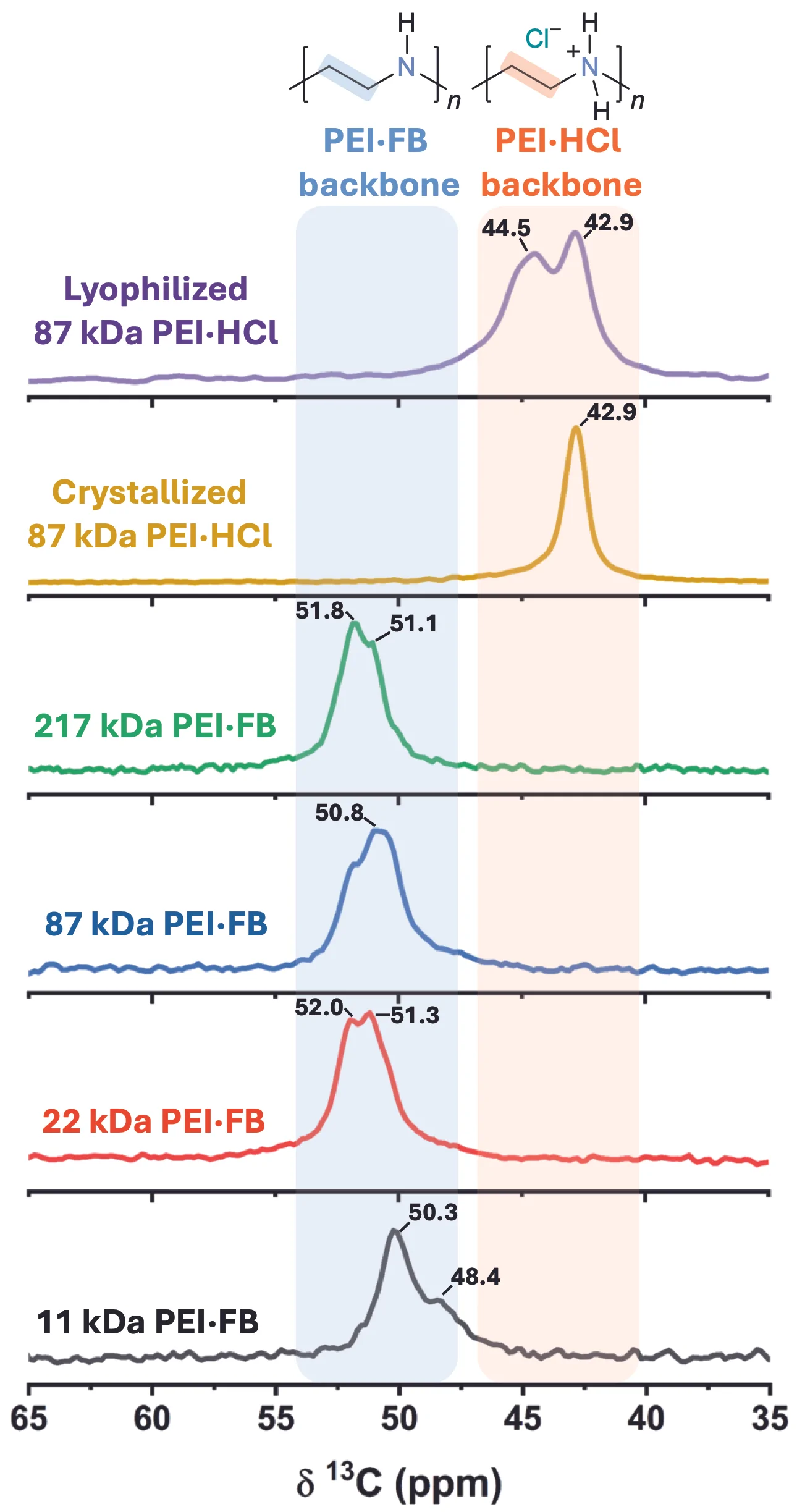
^13^C CP-MAS spectra (MAS rate = 10 kHz, 150.91 MHz) of the PEI·FB and PEI·HCl samples.

**Figure 3:**
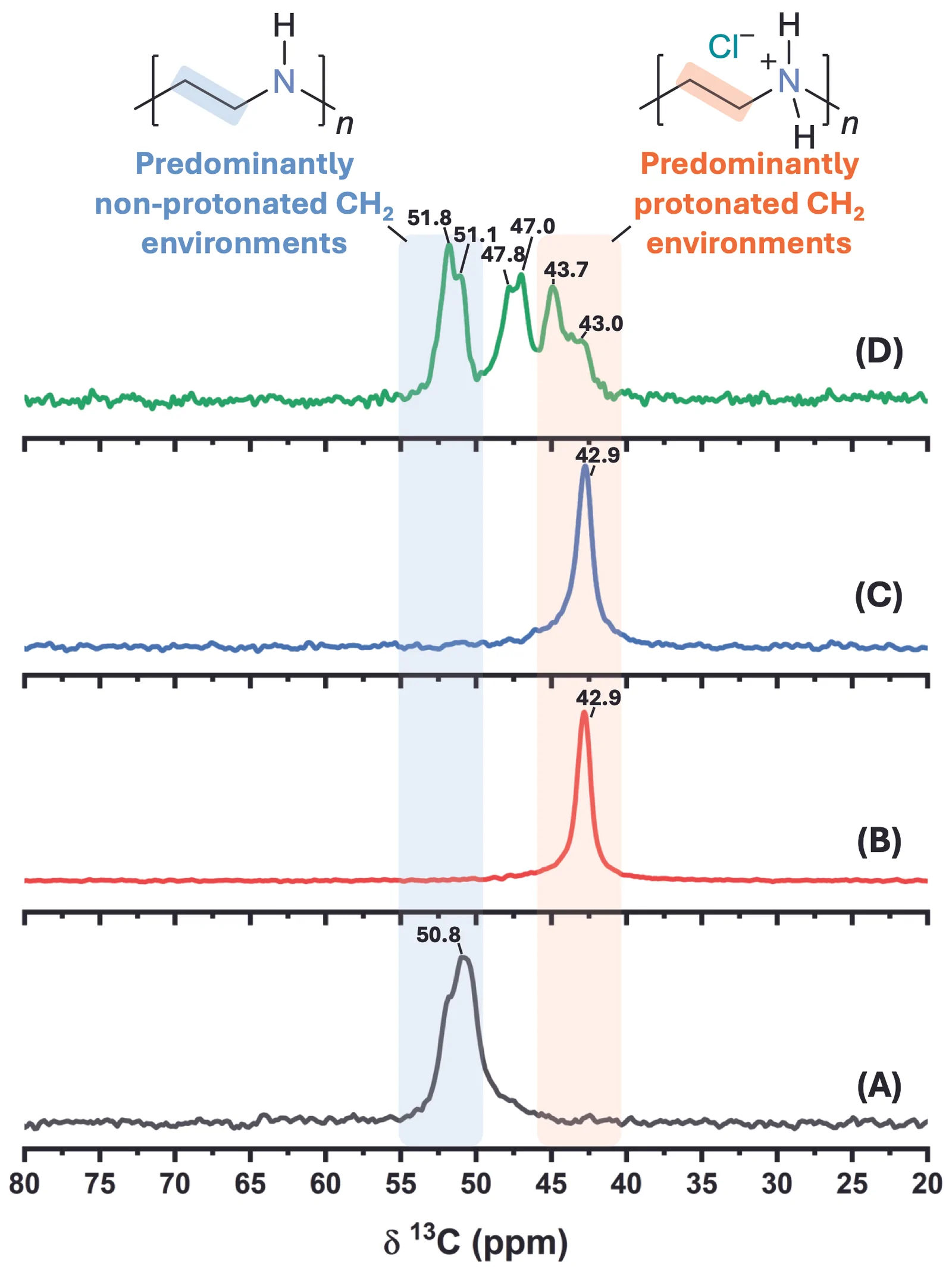
^13^C CP-MAS spectra (MAS rate = 10 kHz, 150.91 MHz) of the PEI·FB (**A**), PEI·HCl as synthesized (**B**), PEI·HCl obtained from the addition of IPA·HCl to a PEI·FB methanolic solution (**C**), and PEI-IPA·HCl (**D**) samples. All the materials were synthesized from the corresponding 87 kDa PEI·FB materials.

**Figure 4:**
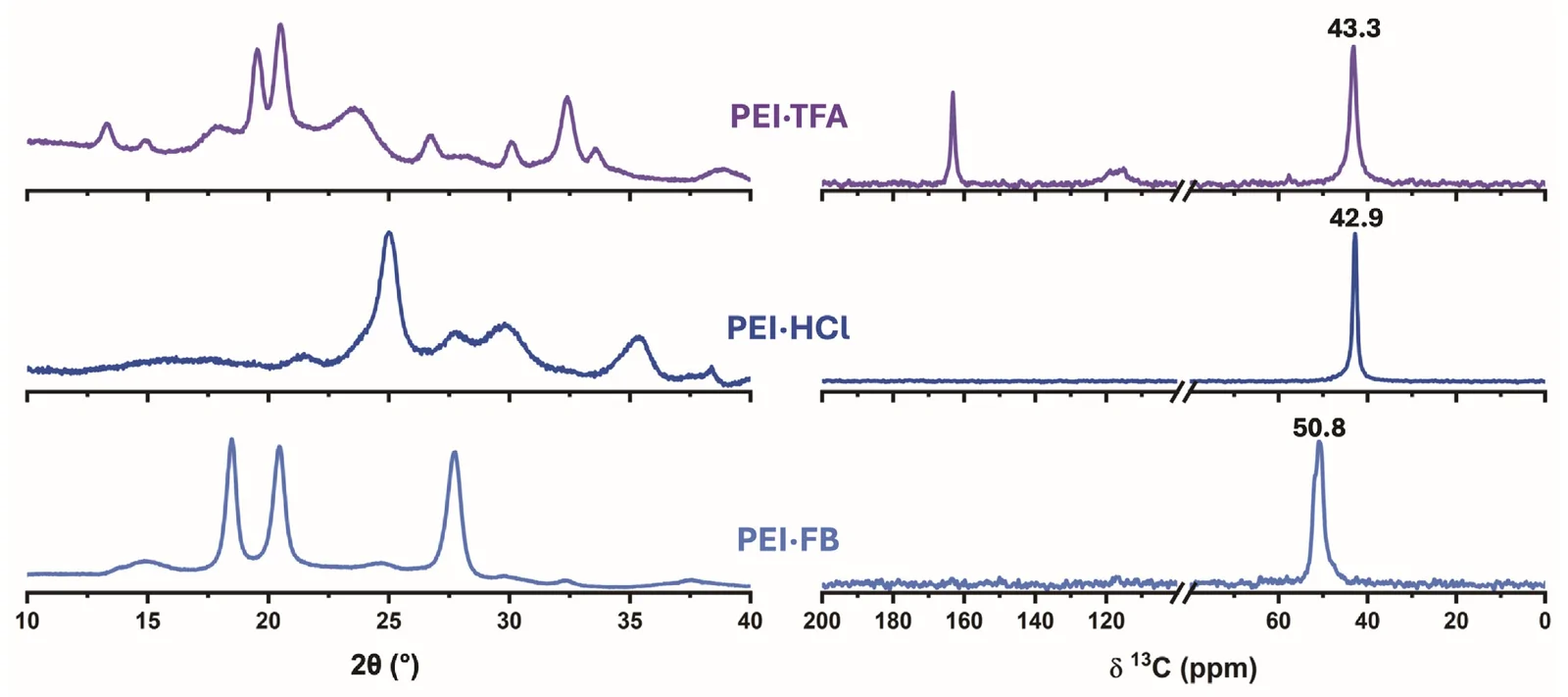
XRD spectra (**left**) and ^13^C CP-MAS spectra (MAS rate = 10 kHz, 150.91 MHz) (**right**) of the 87 kDa PEI materials.

**Figure 5:**
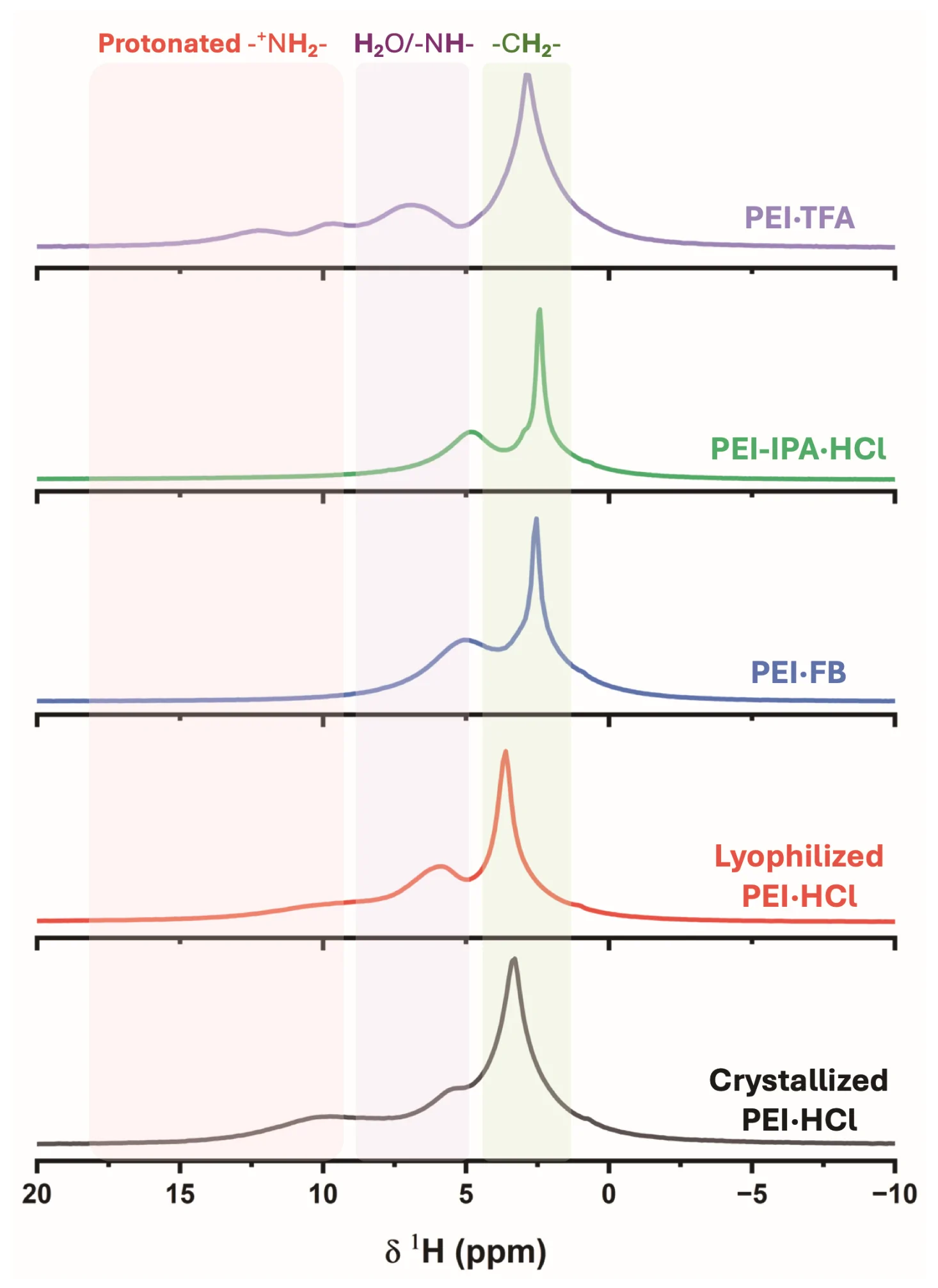
^1^H *ss*-NMR spectra (MAS rate = 60 kHz, 499.70 MHz) of the 87 kDa PEI materials.

**Figure 6:**
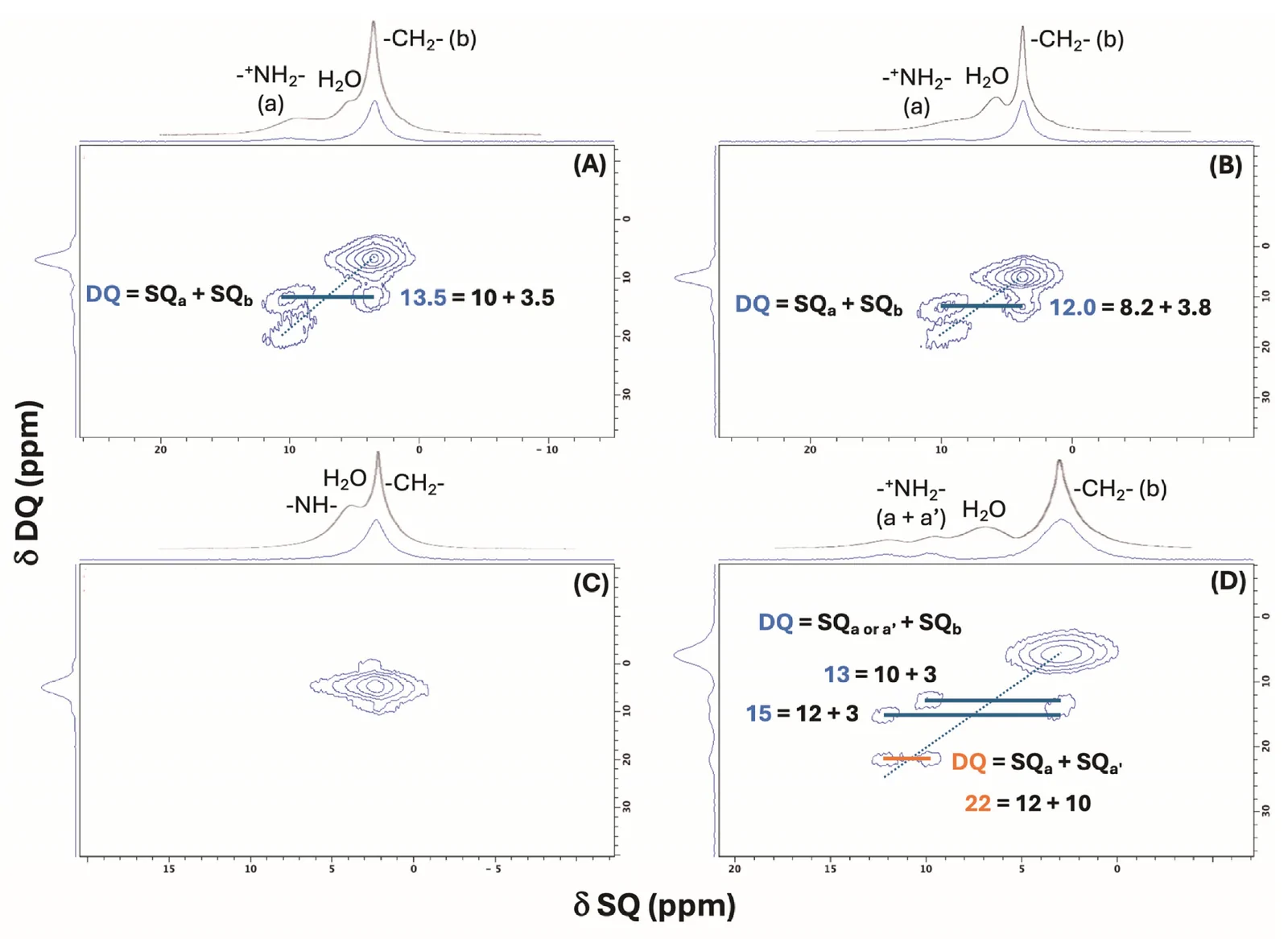
2D SQ-DQ *ss*-NMR spectra (MAS rate = 60 kHz, 499.70 MHz) with two rotor-synchronized times of crystalline (**A**) and lyophilized PEI·HCl (**B**), PEI·FB (**C**) and PEI·TFA (**D**) materials. In all spectra, the ^1^H-MAS spectrum (black line) is superimposed on the SQ dimension from the 2D experiment.

**Figure 7:**
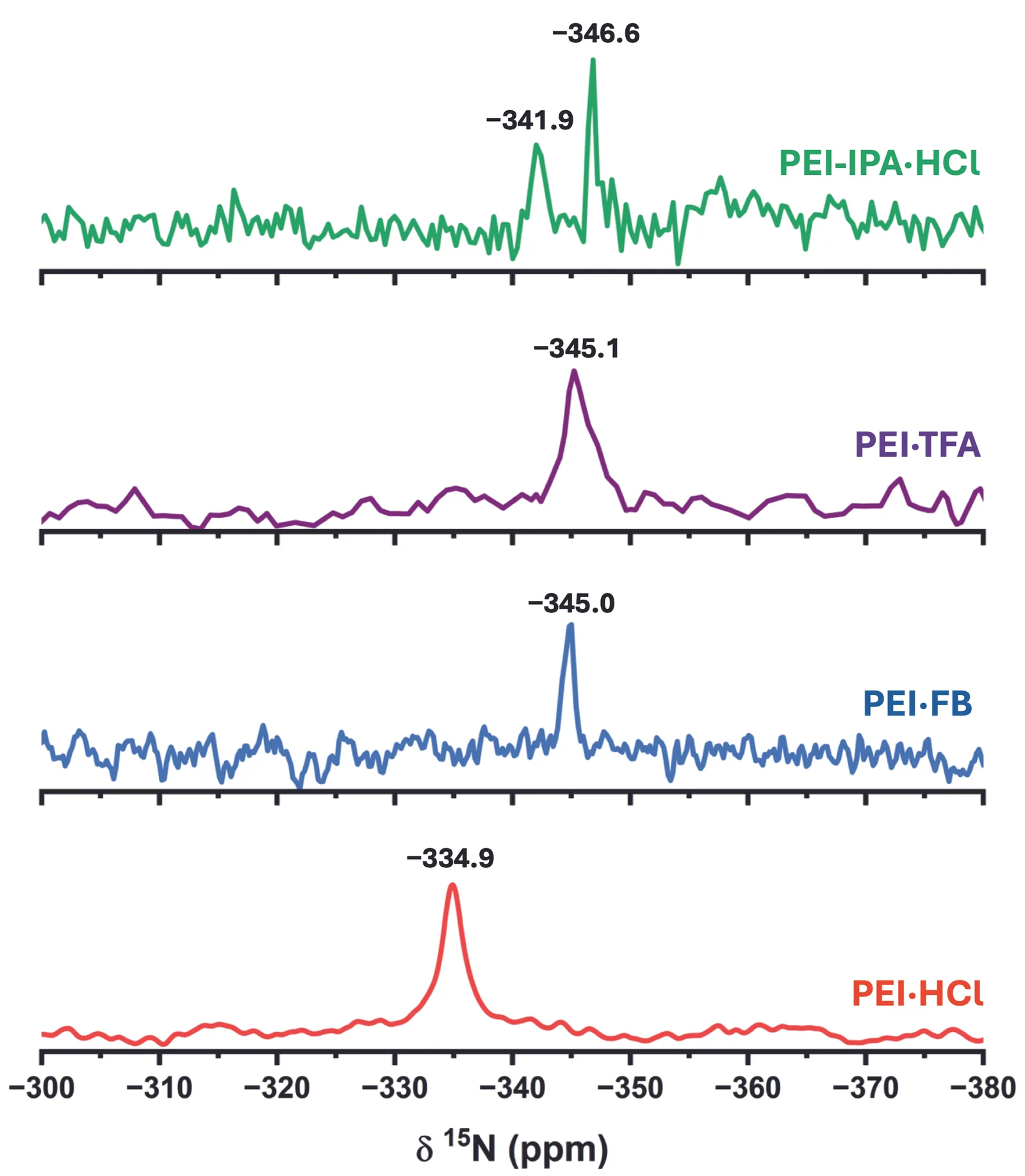
^15^N CP-MAS spectra (MAS rate = 10 kHz, 60.80 MHz) of the 87 kDa PEI materials.

**Figure 8:**
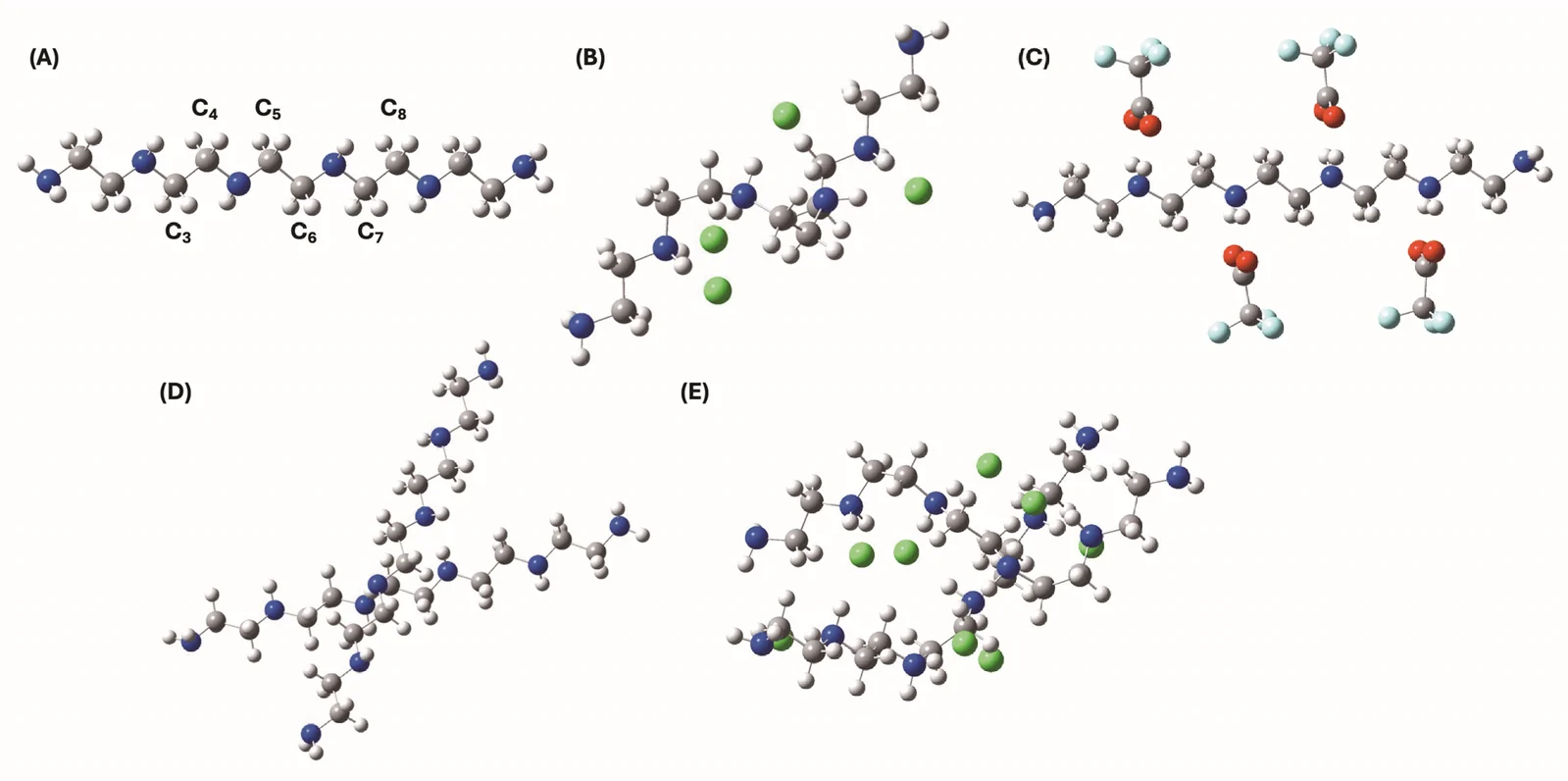
Optimized geometries of the unprotonated linear PEI hexamer (free base, 0% protonation) showing the numbering of the backbone carbon atoms (**A**), the fully protonated PEI·HCl (**B**), the fully protonated PEI·TFA (**C**), and the dimeric PEI models of unprotonated (0%) (**D**) and fully protonated (100%) (**E**).

**Figure 9:**
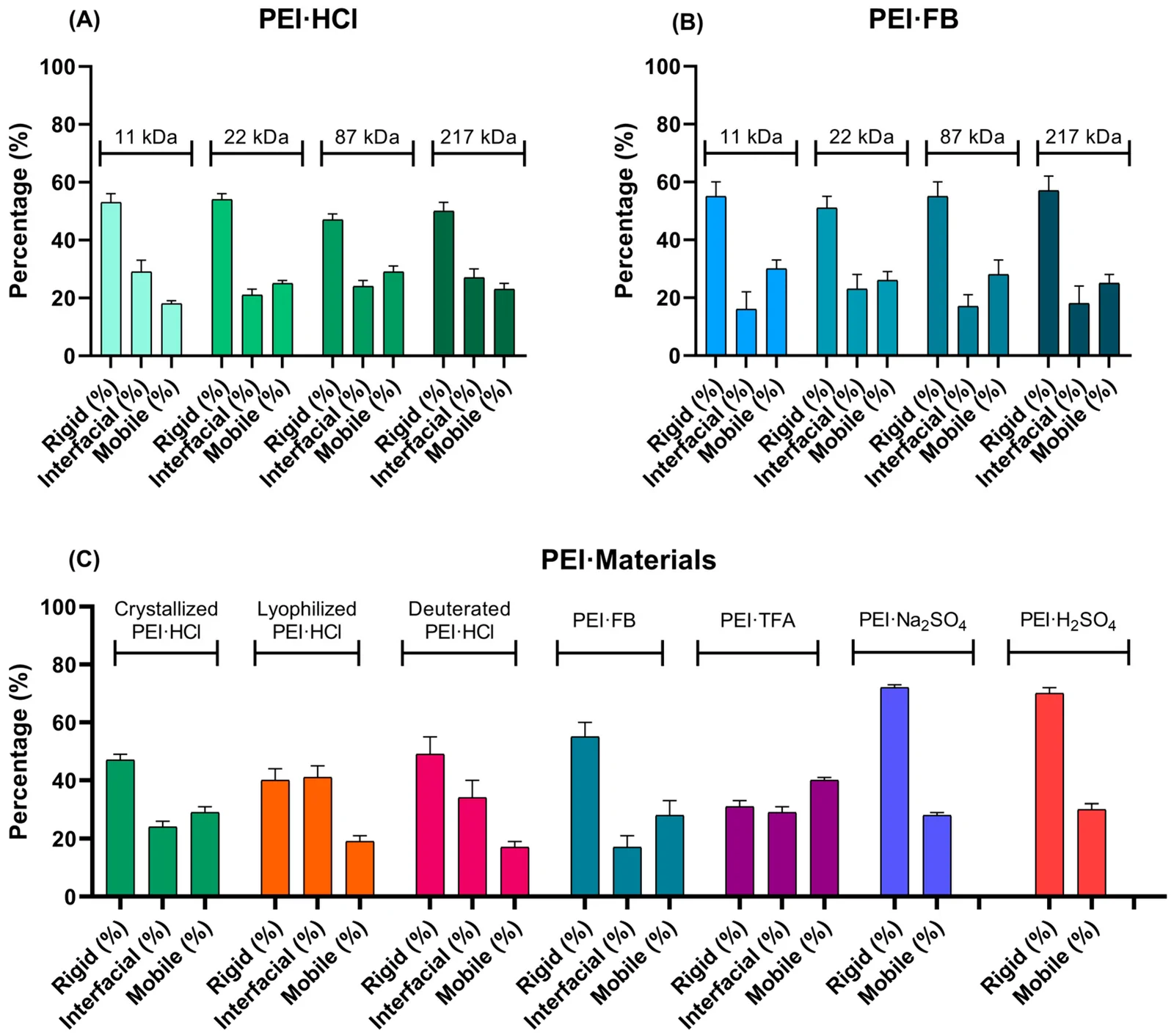
Estimation of the rigid, interfacial and mobile domains for the indicated PEI polymer materials using time-domain NMR studies for the PEI·HCl (**A**) and PEI·FB (**B**) materials with different molecular weights and different 87 kDa PEI materials (**C**).

**Figure 10:**
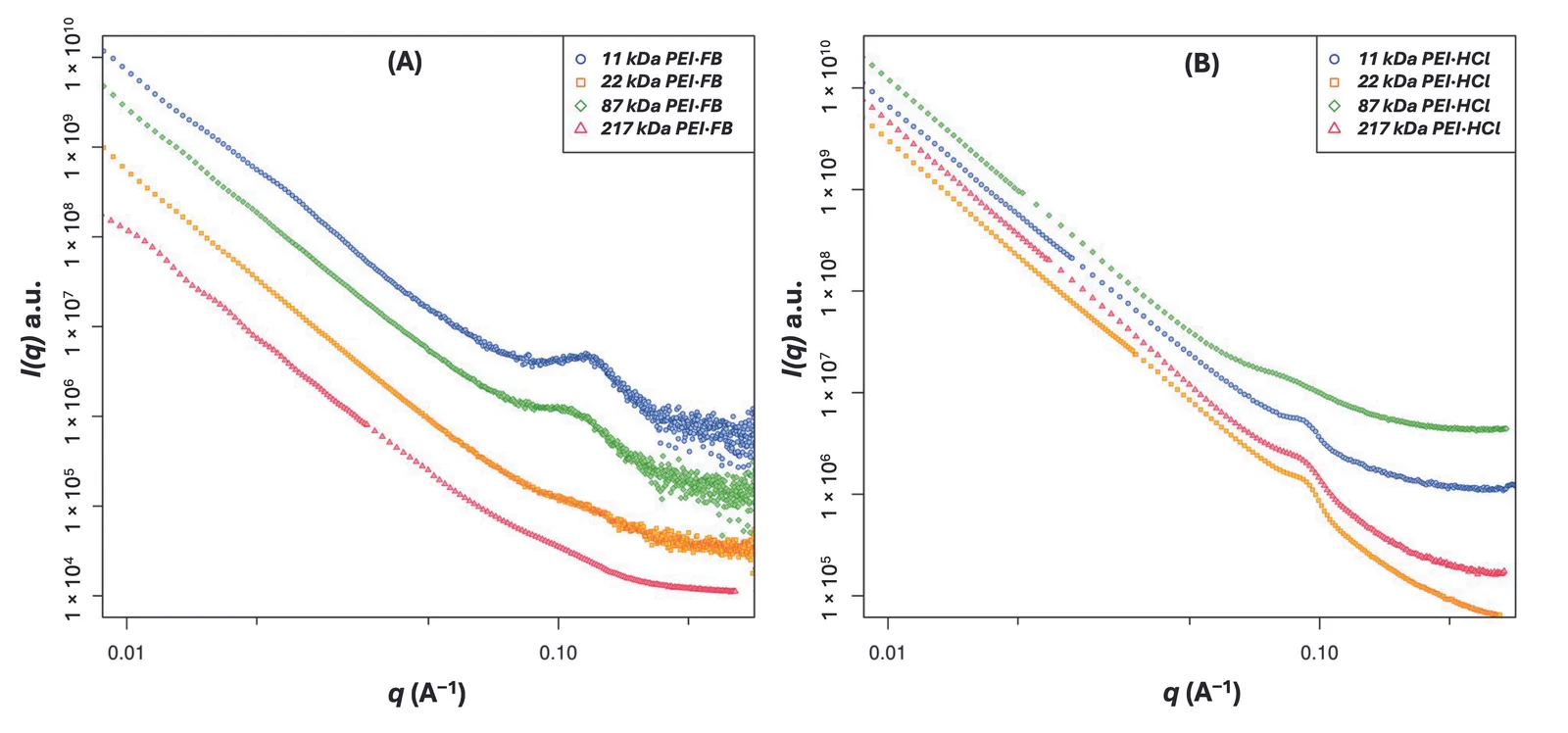
SAXS profiles for PEI·FB (**A**) and PEI·HCl (**B**) samples.

**Figure 11:**
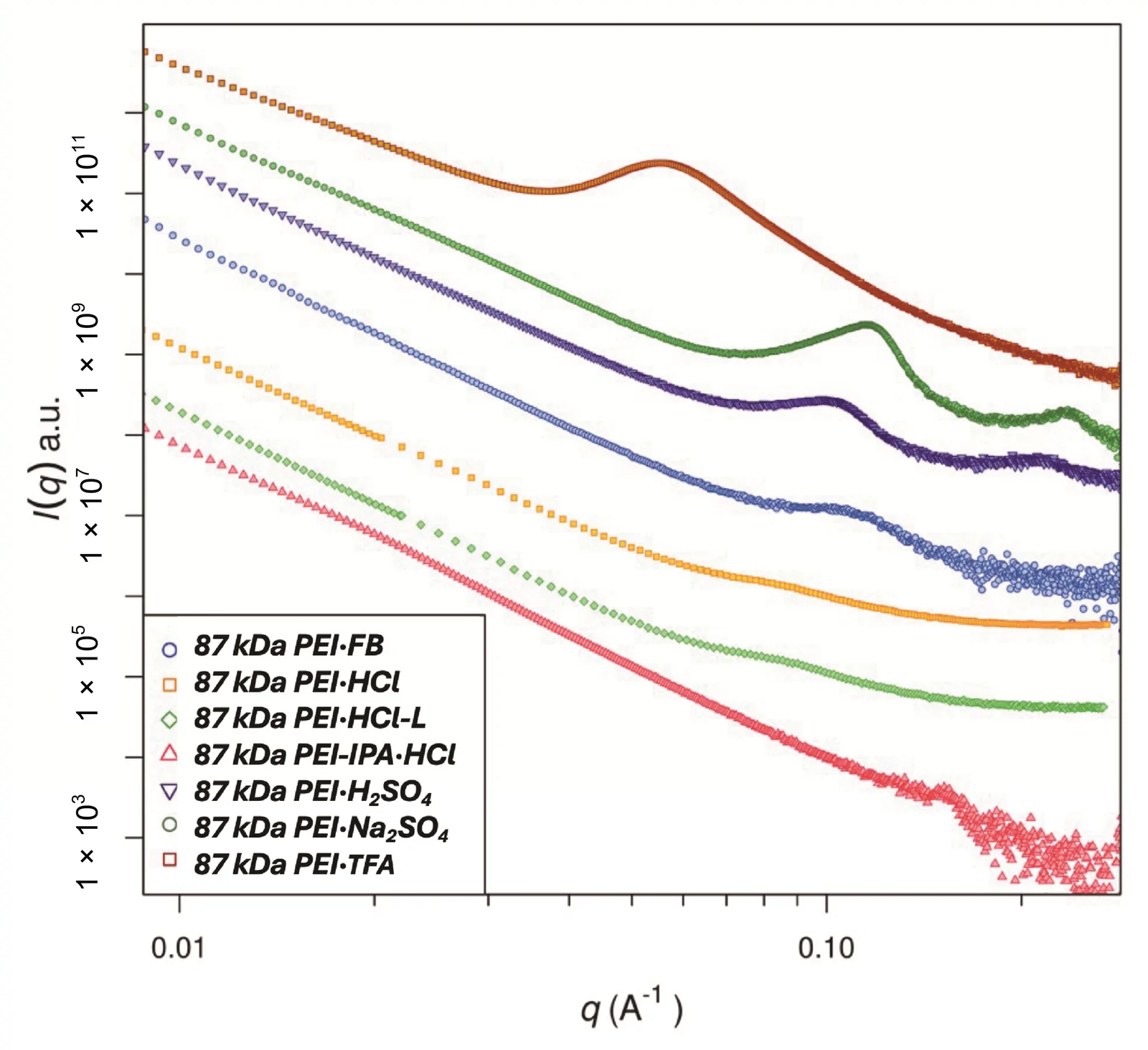
SAXS profiles for PEI polymers with different counterions (Cl^−^, SO_4_^2−^, F_3_CCO_2_^−^) and protonation degrees.

**Figure 12:**
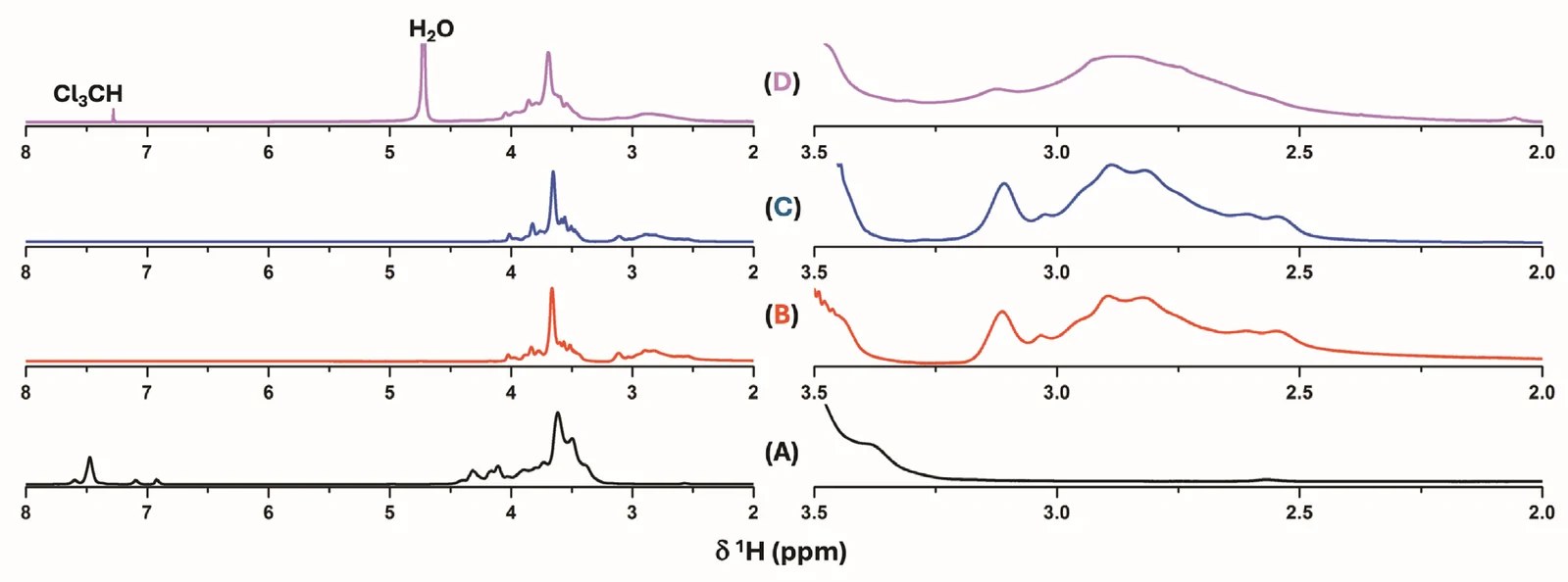
Full (**left)** and partial (**right**) ^1^H HRMAS NMR spectra (MAS rate = 4 kHz, 600.09 MHz) for the following gel materials: EGDE-IM (**A**) and PEI-EGDE synthesized in water (**B**) or acetonitrile (**C**) swollen in D_2_O; and the PEI-EGDE synthesized in water and swollen in CDCl_3_ (**D**).

**Figure 13:**
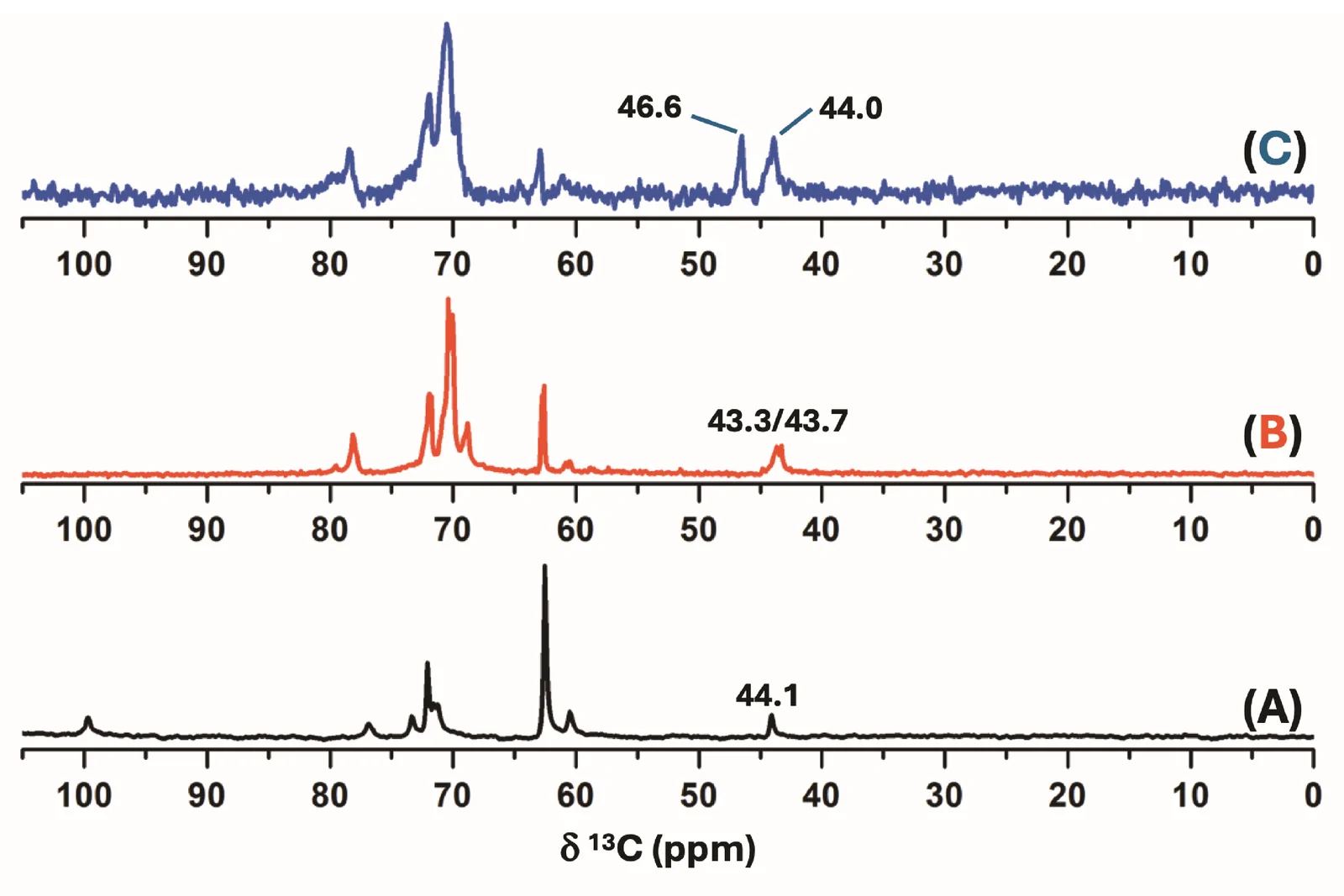
^13^C HRMAS NMR spectra (MAS rate = 4 kHz, 150.91 MHz) for the starch-PEI film (**A**) and PEI-EGDE gel swollen in D_2_O (**B**) or 100 mM of Na_2_SO_4_ in D_2_O (**C**).

**Scheme 1:**
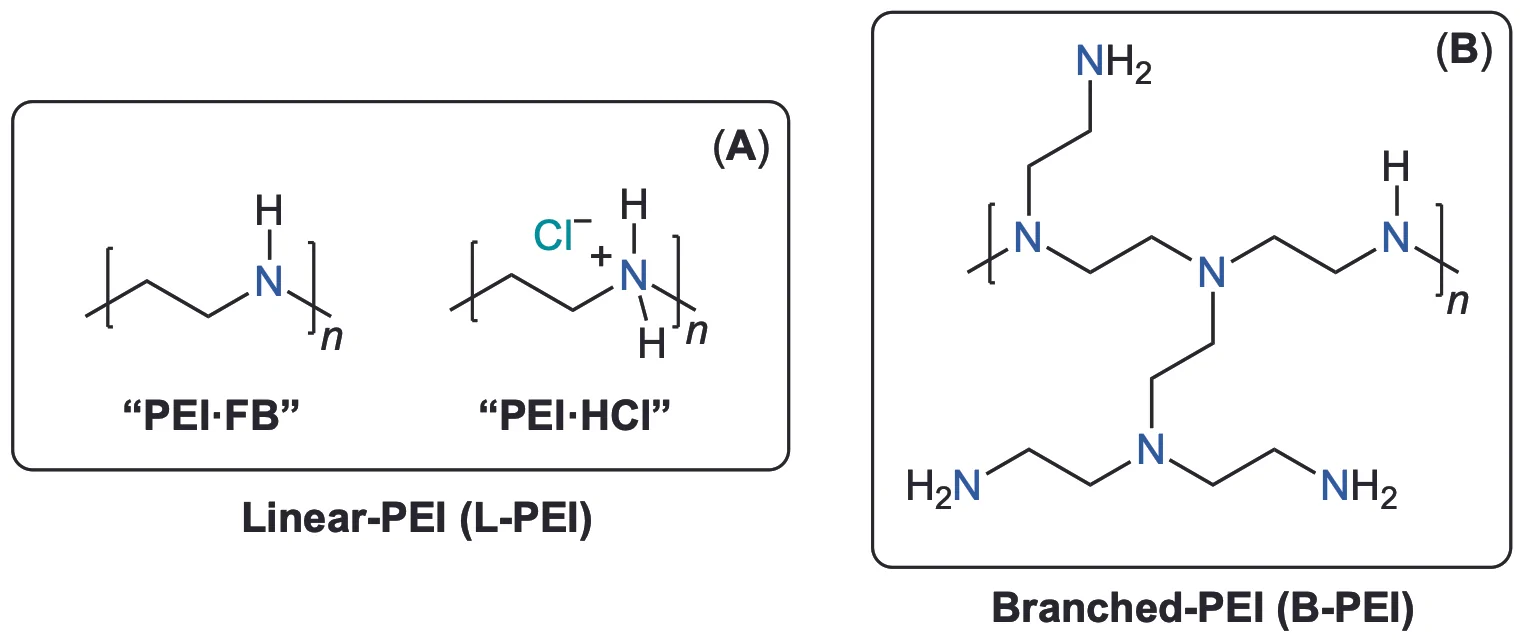
Chemical structures of the L-PEI (**A**) and B-PEI (**B**) polymers.

**Table 1:** Calculated isotropic ^13^C chemical shifts of the internal backbone carbons of the PEI model systems as a function of the protonation degree, the counterion and the packing model.

System	Counterion	Protonation (%)	δ Center (ppm)	δ Range (ppm)	Dispersion (ppm)
Hexamer	-	0	57.2	57.1–57.2	0.2
Hexamer	Cl^−^	25	53.6	51.0–57.0	6.0
Hexamer	Cl^−^	50	52.0	50.8–53.1	2.3
Hexamer	Cl^−^	100	47.9	44.9–49.1	4.3
Hexamer	CF_3_CO_2_^−^	25	53.3	49.5–56.8	7.3
Hexamer	CF_3_CO_2_^−^	50 (*trans*)	49.6	45.7–52.8	7.1
Hexamer	CF_3_CO_2_^−^	50 (*cis*)	51.2	46.7–54.5	7.8
Hexamer	CF_3_CO_2_^−^	100	45.7	45.0–46.7	1.7
Dimer	-	0	56.7	55.9–57.3	1.4
Dimer	Cl^−^	100	48.5	46.4–50.3	3.9

**Table 3:** Abragam function and exponential decay (*T_2_*) parameters obtained from TD-NMR (MSE) fitting for the indicated PEI polymer materials. The a and b are the Gaussian decay time and damping/oscillation frequency of the Abragam (rigid-domain) term, respectively (Equation (S2), see Supplementary Information).

Material	a (ms)	b (kHz)	T_2_ Interfacial (ms)	T_2_ Mobile (ms)
11 kDa PEI·FB	0.022 ± 0.003	161.5 ± 8.7	0.018 ± 0.006	0.072 ± 0.004
22 kDa PEI·FB	0.023 ± 0.003	168.3 ± 8.3	0.020 ± 0.005	0.077 ± 0.005
87 kDa PEI·FB	0.018 ± 0.002	145.6 ± 14.9	0.035 ± 0.016	0.097 ± 0.010
217 kDa PEI·FB	0.022 ± 0.002	162.6 ± 7.8	0.016 ± 0.004	0.070 ± 0.004
11 kDa PEI·HCl	0.022 ± 0.003	156.6 ± 9.2	0.025 ± 0.003	0.494 ± 0.030
22 kDa PEI·HCl	0.022 ± 0.002	160.9 ± 6.9	0.034 ± 0.004	0.694 ± 0.053
87 kDa PEI·HCl	0.023 ± 0.002	161.8 ± 6.5	0.032 ± 0.003	1.338 ± 0.205
217 kDa PEI·HCl	0.023 ± 0.003	158.3 ± 8.1	0.025 ± 0.003	0.781 ± 0.064
87 kDa Deuterated PEI·HCl	0.020 ± 0.004	134.8 ± 21.5	0.024 ± 0.004	0.381 ± 0.028
87 kDa Lyophilized PEI·HCl	0.022 ± 0.004	151.0 ± 13.2	0.024 ± 0.002	0.870 ± 0.107
87 kDa PEI·TFA	0.028 ± 0.004	203.4 ± 6.2	0.027 ± 0.003	0.167 ± 0.004
87 kDa PEI·Na_2_SO_4_ ^(1)^	0.021 ± 0.001	138.7 ± 4.1	—	0.057 ± 0.002
87 kDa PEI·H_2_SO_4_ ^(1)^	0.023 ± 0.001	135.5 ± 3.9	—	0.055 ± 0.002

^(1)^ For 87 kDa PEI·Na_2_SO_4_ and PEI·H_2_SO_4_, interfacial and mobile *T*_2_ were not resolved separately; the single value shown corresponds to the combined mobile-like component from the two-component model [38,39,40,64].

**Table 4:** SAXS power-law, Lorentzian peak model parameters for PEI free bases and hydrochlorides.

PEI Material	α	Ds	Peak *q* (Å^−1^)	Peak *d* (Å)	χ^2^ ^(1)^
11 kDa PEI·FB	3.877 ± 0.002	2.123 ± 0.002	0.1124 ± 0.0002	55.90 ± 0.10	12.226
22 kDa PEI·FB	3.992 ± 0.002	2.008 ± 0.002	0.0933 ± 0.001	n.d. ^(2)^	1.9554
87 kDa PEI·FB	3.893 ± 0.002	2.107 ± 0.002	0.1003 ± 0.0002	62.6 ± 0.1	2.6112
217 kDa PEI·FB	3.944 ± 0.001	2.056 ± 0.001	0.0673 ± 0.0005	n.d. ^(2)^	79.837
11 kDa PEI·HCl	3.5234 ± 0.0009	2.4766 ± 0.0009	0.0894 ± 0.0001	70.28 ± 0.08	17.105
22 kDa PEI·HCl	3.6471 ± 0.0006	2.3529 ± 0.0006	0.08905 ± 3 × 10^−5^	70.56 ± 0.02	157.64
87 kDa PEI·HCl	3.645 ± 0.002	2.355 ± 0.002	0.0824 ± 0.0003	76.3 ± 0.3	1.6831
217 kDa PEI·HCl	3.7115 ± 0.0008	2.2885 ± 0.0008	0.08851 ± 7 × 10^−5^	70.99 ± 0.06	16.624

^(1)^ Chi-squared goodness-of-fit for the combined model. ^(2)^ Not determined due to diffuse maximum.

**Table 5:** SAXS power-law, Lorentzian peak model parameters of 87 kDa PEI samples.

PEI Material	α	Ds	Peak *q* (Å^−1^)	Peak *d* (Å)	χ^2 (1)^	2nd Peak q (Å^−1^)	2nd Peak *d* (Å)	χ^2 (2)^
PEI·FB	3.893 ± 0.002	2.107 ± 0.002	0.1003 ± 0.0002	62.64 ± 0.12	2.6			
PEI·HCl	3.645 ± 0.002	2.355 ± 0.002	0.0824 ± 0.0003	76.25 ± 0.28	1.7			
PEI·HCl-L ^(3)^	3.809 ± 0.002	2.191 ± 0.002	0.0813 ± 0.0003	77.28 ± 0.29	1.2			
PEI-IPA·HCl	4.058 ± 0.001	1.942 ± 0.001	0.112 ± 0.001	56.10 ± 0.50	10.7			
PEI·H_2_SO_4_	3.695 ± 0.002	2.305 ± 0.002	0.09700 ± 0.00005	64.78 ± 0.03	9.1	0.2035 ± 0.0006	30.88 ± 0.09	1.3
PEI·Na_2_SO_4_	3.614 ± 0.002	2.386 ± 0.002	0.11081 ± 0.00002	56.70 ± 0.01	120.8	0.2209 ± 0.0004	28.44 ± 0.05	4.1
PEI·TFA	3.454 ± 0.002	2.546 ± 0.002	0.055929 ± 0.000004	112.34 ± 0.01	190.4			

^(1)^ Chi-squared goodness-of-fit for the combined model. ^(2)^ Chi-squared goodness-of-fit for the higher *q* Lorentzian peak model. ^(3)^ PEI·HCl-L refers to the lyophilized sample of the PEI·HCl.

## Data Availability

The original contributions presented in this study are included in the article/Supplementary Materials.

## Supplementary Materials

The following supporting information can be downloaded at: https://www.mdpi.com/article/10.3390/gels12100912/s1. Figure S1: XRD spectra for the dihydrate (A) or monohydrate (B) forms for the 22 kDa PEI·FB material and hemihydrate (C) or monohydrate (D) forms for the 87 kDa PEI·FB material. The asterisks indicate the signals corresponding to sodium bicarbonate within the sample. Figure S2: ^1^H- (A) and ^13^C-NMR spectra (B) for the 87 kDa PEI·FB sample dissolved in methanol-*d*_4_. Figure S3: ATR-FTIR spectra for the 87 PEI kDa samples: PEI·TFA (A), PEI·HCl (B) and PEI·FB (C). Figure S4: (I) XRD spectra for the PEI·H_2_SO_4_ and PEI·Na_2_SO_4_ samples, (II): *solid-state* NMR results for the PEI·H_2_SO_4_ and PEI·Na_2_SO_4_ samples, ^13^C CP-MAS (@10 kHz) (A), ^1^H-MAS (@60 kHz) and ^15^N CP-MAS (@10 kHz) (C) spectra. Figure S5: Optimized geometries of the linear PEI hexamer models with trifluoroacetate as counterion, at increasing protonation degrees: 25% (A), 50% (*trans*) (B), 50% (*cis*) (C), and 100% (D). Figure S6: Optimized geometries of the L-PEI hexamer models with chloride as counterion, at increasing protonation degrees: 25% (A), 50% (*trans*) (B), 50% (*cis*) (C) and 100% (D). Figure S7: Fitting of SAXS results for PEI·FB and PEI·HCl samples. Figure S8: Fitting of SAXS results for the 87 kDa PEI materials. Figure S9: SAXS fitting errors (Residual weights). Figure S10: *Solution-state* ^1^H-NMR spectra (600.09 MHz) of PEOX 50 kDa (D_2_O), PEI·HCl 22 kDa (D_2_O) and PEI·FB 22 kDa (CD^3^OD). Synthetic procedures (SP): Synthesis of PEI·FB, PEI·TFA, crystallized PEI·HCl, PEI-IPA·HCl, PEI·Na_2_SO_4_, PEI·H_2_SO_4_, deuterated PEI·HCl and lyophilized PEI·HCl samples. Figure S12: Panoramic XPS spectra for the PEI·HCl (A), PEI·Na_2_SO_4_ (B) and PEI·H_2_SO_4_ (C) samples. Figure S13: ^1^H-MAS solid-state NMR spectra (MAS rate = 60 kHz, 499.70 MHz) for the 87 kDa PEI·HCl and deuterated 87 kDa PEI·HCl samples. Characterization Techniques (CT): General procedures, XRD, NMR, computational methodology, TD-NMR, SAXS and swelling studies. Table S1: Comparison between the experimental XRD (Figure 1) and the theoretical XRD according to Chatani et al. [38,39,40]. Table S2: Calculated ^13^C values (relative to TMS) for free base and hydrochloride hexamers with 25% protonation state; Table S3: Calculated ^13^C values (relative to TMS) for hydrochloride hexamers with 50% protonation state (*cis* and *trans*); Table S4: Calculated ^13^C values (relative to TMS) for hydrochloride hexamers with 100% protonation state; Table S5: Calculated ^13^C values (relative to TMS) for trifluoroacetate hexamers with 25% and 100% protonation state; Table S6: Calculated ^13^C values (relative to TMS) for trifluoroacetate hexamers with 50% protonation state (*cis* and *trans*); Table S7: Calculated ^13^C values (relative to TMS) for dimers with 0% and 100% protonation state; Table S8: Calculated (relative to nitromethane) and experimental ^15^N chemical shifts of the internal backbone nitrogen atoms for the free base and the fully protonated PEI salts; Table S9: Calculated ^15^N values (relative to nitromethane) for trifluoroacetate hexamers with 50% protonation state (*cis* and *trans*); Table S10: Calculated ^15^N values (relative to nitromethane) for hydrochloride hexamers with 50% protonation state (*cis* and *trans*); (DFT calculations): XYZ coordinates of PEI derivatives (DFT calculations); TD-NMR fittings. Table S11: SAXS power-law, Lorentzian peak model parameters for PEI free bases and hydrochlorides. Table S12: SAXS power-law, Lorentzian peak model parameters of 87 kDa PEI samples. Table S13: Organic elemental analysis in PEI·TFA sample. Figure S11: *Solution-state* ^19^F-NMR (564.69 MHz) of PEI·TFA sample with the addition of NaF in D^2^O. Table S14: Organic elemental analysis in PEI·sulfate samples. References [25,38,39,40,63,69,70,71,72].
