# Supplementary material for: Insights into the Protonation State and Counterion Interactions of Linear Polyethyleneimine Materials
Source: Gels. 2026 Oct 10;12(10):912. doi: 10.3390/gels12100912 (PMC13704144; doi:10.3390/gels12100912)
Supplement: Supplementary file 1 [file gels-12-00912-s001.zip › gels-4520396-supplementary.pdf]

-Supplementary Material-

Insights into the protonation state and counterion interactions of linear polyethyleneimine materials

Agustín J. Byrne [1,2], Daniel Vladimir [1,3,4], Yamila Garro-Linck [5,6], Guillermo J. Copello [1,3], Ayelén F. Crespi [1,3], Daniel Vega [7], Mariela Bollini [4], Paula G. Franco [1,2] and Juan M. Lázaro-Martínez [1,3,*]

[1]Universidad de Buenos Aires, Facultad de Farmacia y Bioquímica, Ciudad Autónoma de Buenos Aires 1113. Argentina.

[2]CONICET – Universidad de Buenos Aires, Instituto de Química y Fisicoquímica Biológicas (IQUIFIB), Ciudad Autónoma de Buenos Aires 1113, Argentina.

[3]CONICET – Universidad de Buenos Aires, Instituto de Química y Metabolismo del Fármaco (IQUIMEFA-UBA-CONICET), Ciudad Autónoma de Buenos Aires 1113, Argentina.

[4]Centro de Investigaciones en Bionanociencias (CIBION – CONICET), Polo Científico Tecnológico, Ciudad Autónoma de Buenos Aires 1425, Argentina.

[5]Universidad Nacional de Córdoba, Facultad de Matemática, Astronomía, Física y Computación, Córdoba 5000, Argentina.

[6]Consejo Nacional de Investigaciones Científicas y Técnicas (CONICET), Instituto de Física Enrique Gaviola (IFEG), Córdoba 5000, Argentina.

[7]Comisión Nacional de Energía Atómica, San Martín, Buenos Aires 1650, Argentina.

*Correspondence: lazarojm@ffyb.uba.ar (J.M.L.-M.); Tel.: +54-11-5287-4323

Table of Content	*Page*
Figure S1 (XRD)	3
Table S1 (XRD)	4
Figure S2 (solution-state NMR)	6
Figure S3 (ATR-FTIR)	7
Figure S4 (XRD and solid-state NMR)	8
Figure S5 (DFT calculations)	9
Figure S6 (DFT calculations)	10
Tables S2-S10 (DFT Calculations)	11
XYZ coordinates of PEI materials	15
TD-NMR Fittings	31
Figure S7 (SAXS)	34
Figure S8 (SAXS)	35
Tables S11-S12 (SAXS)	36
SAXS Fitting Errors (Residual weights) (Figure S9)	37
Synthetic Procedures (SP)	39
Synthesis of PEI free bases (PEI·FB) (Figure S10)	39
Synthesis of PEI trifluoroacetate (PEI·TFA) (Table S13 and Figure S11)	40
Synthesis of PEI·HCl from PEI·FB (crystallized PEI·HCl)	40
Synthesis of PEI-IPA·HCl from PEI·FB	40
Synthesis of PEI·Na_2SO_4 and PEI·H_2SO_4 (Table S14)	41
Panoramic XPS spectra of PEI·Na_2SO_4 and PEI·H_2SO_4 (Figure S12)	42
Synthesis of deuterated PEI·HCl (Figure S13)	42
Synthesis of lyophilized 87 kDa PEI·HCl	43
Characterization Techniques (CT)	44
General characterization techniques	44
X-ray powder diffraction (XRD) studies	44
NMR studies	44
Computational methodology	45
Time-domain NMR studies	46
Small Angle X-ray Scattering studies	46
Swelling studies	47

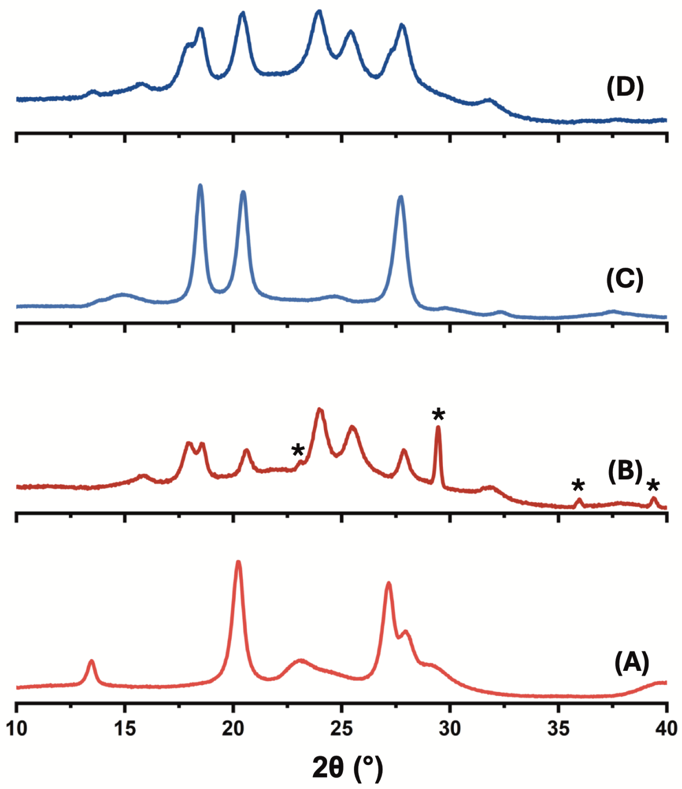

Figure S1. XRD spectra for the dihydrate (A) or monohydrate (B) forms for the 22 kDa PEI·FB material, and hemihydrate (C) or monohydrate (D) forms for the 87 kDa PEI·FB material. The asterisks indicate the signals corresponding to sodium bicarbonate within the sample.

Experimental procedures:

-Figure S1A: The sample of 22 kDa PEI·FB was obtained following the procedure in "Synthesis of PEI·FB materials".

-Figure S1B: The sample of 22 kDa PEI·FB was obtained following the procedure in "Synthesis of PEI·FB materials", but when the water was not freshly distilled (pH < 6.0) during the neutralization process the presence of the bicarbonate ion was detected by XRD.

-Figure S1C: The sample of 87 kDa PEI·FB was obtained following the procedure in "Synthesis of PEI·FB materials".

-Figure S1D: The sample of 87 kDa PEI·FB was obtained following the procedure in "Synthesis of PEI·FB materials", but the NaOH solution was added at once during the neutralization process.

Table S1. Comparison between the experimental XRD (Figure 1) and the theoretical XRD according to Chatani et al. [1–3].

**11 kDa PEI·FB**									
Reference phase: dihydrate									
**Peak**	**2θ exp. (°)**	**2θ theor. (°)**	**d exp. (Å)**	**d theor. (Å)**	**Δ2θ (°)**	**Δd (Å)**	**Candidate *hkl***		
							**h**	**k**	**l**
**1**	13.66	13.61	6.48	6.51	0.06	-0.03	2	0	0
**2**	20.46	20.44	4.34	4.35	0.02	-0.01	1	1	0
**3**	23.52	23.20	3.78	3.83	0.31	-0.05	1	1	-1
**4**	27.40	27.41	3.26	3.25	-0.01	0.00	4	0	0
**5**	28.22	28.25	3.16	3.16	-0.03	0.00	3	1	0
**6**	29.40	29.24	3.04	3.06	0.16	-0.02	3	1	-1

**22 kDa PEI·FB**									
Reference phase: dihydrate									
**Peak**	**2θ exp. (°)**	**2θ theor. (°)**	**d exp. (Å)**	**d theor. (Å)**	**Δ2θ (°)**	**Δd (Å)**	**Candidate *hkl***		
							**h**	**k**	**l**
1	13.45	13.61	6.58	6.51	-0.156	0.075	2	0	0
2	20.22	20.44	4.39	4.35	-0.217	0.046	1	1	0
3	23.23	23.20	3.83	3.83	0.029	-0.005	1	1	-1
4	27.15	27.41	3.28	3.25	-0.258	0.030	4	0	0
5	27.97	28.25	3.19	3.16	-0.275	0.030	3	1	0
6	29.14	29.24	3.07	3.06	-0.100	0.010	3	1	-1

**87 kDa PEI·FB**									
Reference phase: hemihydrate									
**Peak**	**2θ exp. (°)**	**2θ theor. (°)**	**d exp. (Å)**	**d theor. (Å)**	**Δ2θ (°)**	**Δd (Å)**	**Candidate *hkl***		
							**h**	**k**	**l**
1	14.83	15.31	5.97	5.79	-0.482	0.187	1	1	-1
2	18.47	18.64	4.80	4.76	-0.173	0.044	0	2	0
3	20.44	20.59	4.35	4.31	-0.146	0.031	2	0	0
4	24.69	24.43	3.61	3.64	0.261	-0.038	2	0	-2
5	27.70	27.91	3.22	3.20	-0.213	0.024	2	2	0
6	32.34	32.52	2.77	2.75	-0.178	0.015	3	1	0
7	37.85	37.82	2.38	2.38	0.028	-0.002	0	4	0

**217 kDa PEI·FB**

Peak	2θ exp. (°)	2θ theor. (°)	d exp. (Å)	d theor. (Å)	Δ2θ (°)	Δd (Å)	Candidate *hkl*		
							h	k	l
Reference phase: Hemihydrate									
1	13.48	13.85	6.57	6.39	-0.376	0.177	1	1	0
2	18.44	18.64	4.81	4.76	-0.204	0.052	0	2	0
3	20.26	20.59	4.38	4.31	-0.329	0.069	2	0	0
4	23.42	24.20	3.80	3.68	-0.778	0.120	0	2	1
5	27.14	26.51	3.29	3.36	0.621	-0.076	3	1	-1
6	27.76	27.91	3.21	3.20	-0.148	0.017	2	2	0
7	29.26	30.00	3.05	2.98	-0.743	0.074	1	3	0
Reference phase: Dihydrate									
1	13.48	13.61	6.57	6.51	-0.130	0.062	2	0	0
2	20.26	20.44	4.38	4.35	-0.178	0.038	1	1	0
3	23.42	23.20	3.80	3.83	0.220	-0.035	1	1	-1
4	27.14	27.41	3.29	3.25	-0.273	0.032	4	0	0
5	27.76	27.41	3.21	3.25	0.356	-0.041	4	0	0
6	29.26	29.24	3.05	3.06	0.026	-0.003	3	1	-1
7	39.44	39.64	2.29	2.27	-0.204	0.011	5	1	-1

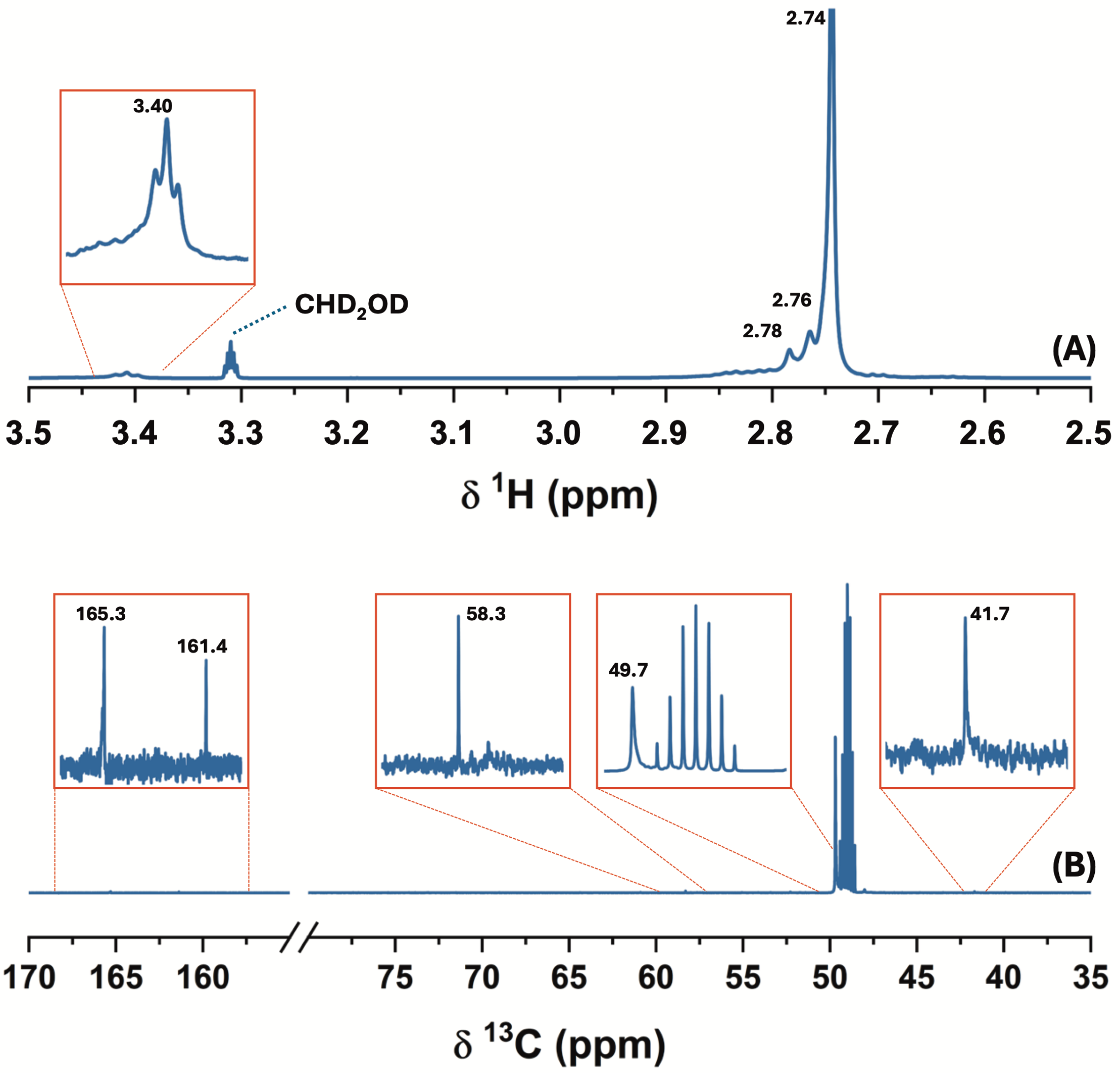

Figure S2. ^{1}H- (600.09 MHz) (A) and ^{13}C-NMR spectra (150.91 MHz) (B) for the 87 kDa PEI·FB sample dissolved in methanol-d_4.

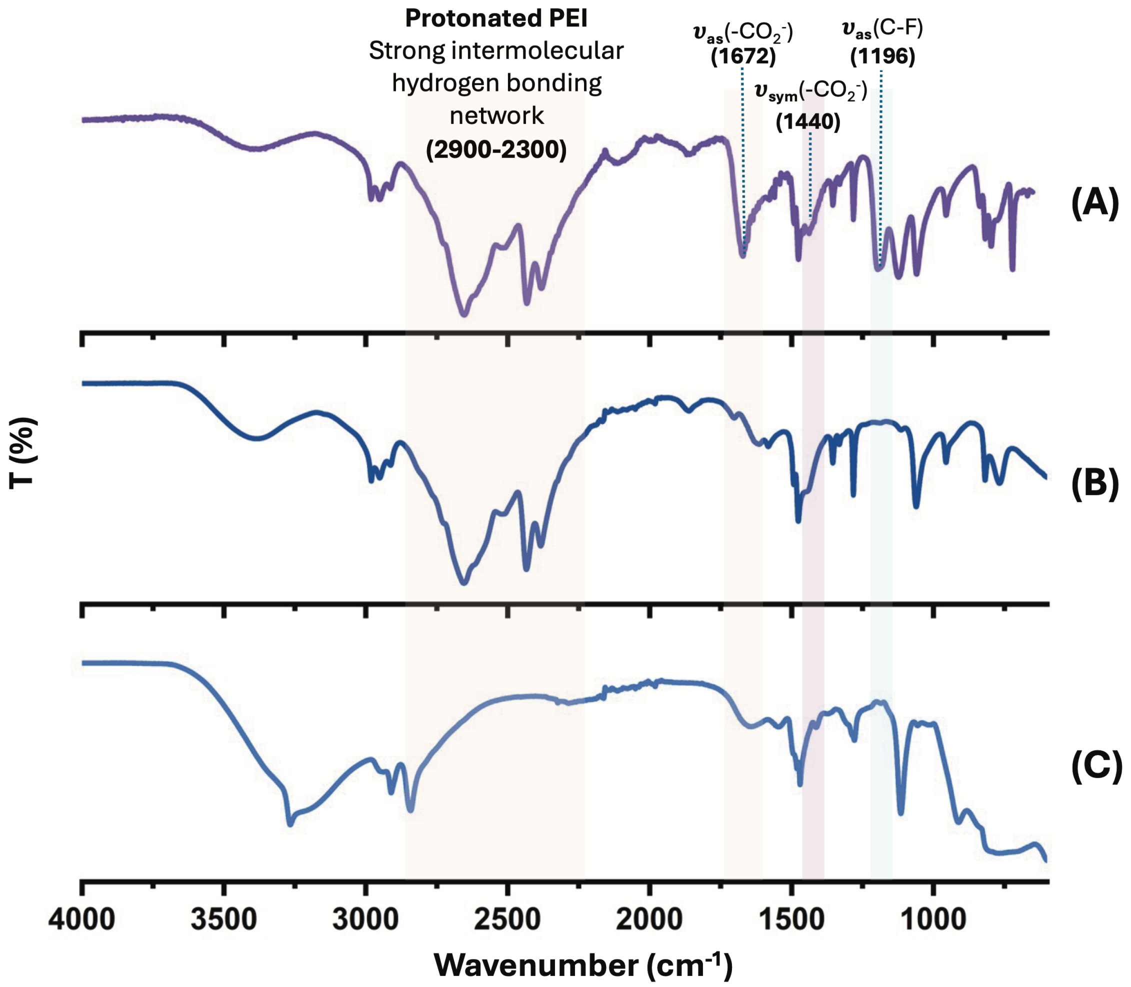

Figure S3. ATR-FTIR spectra for the 87 PEI kDa samples: PEI·TFA (A), PEI·HCl (B) and PEI·FB (C).

-Figure S4-

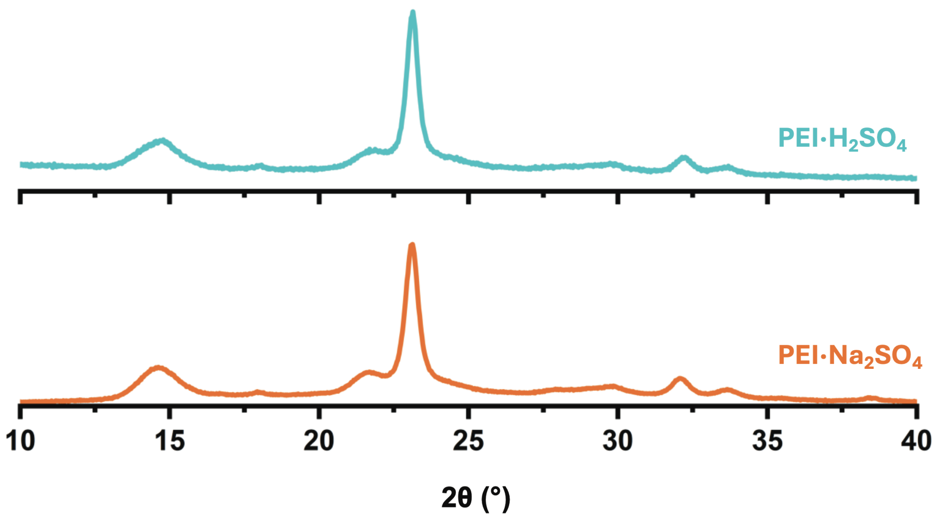

Figure S4 (I). XRD spectra for the PEI·H_2SO_4 and PEI·Na_2SO_4 samples.

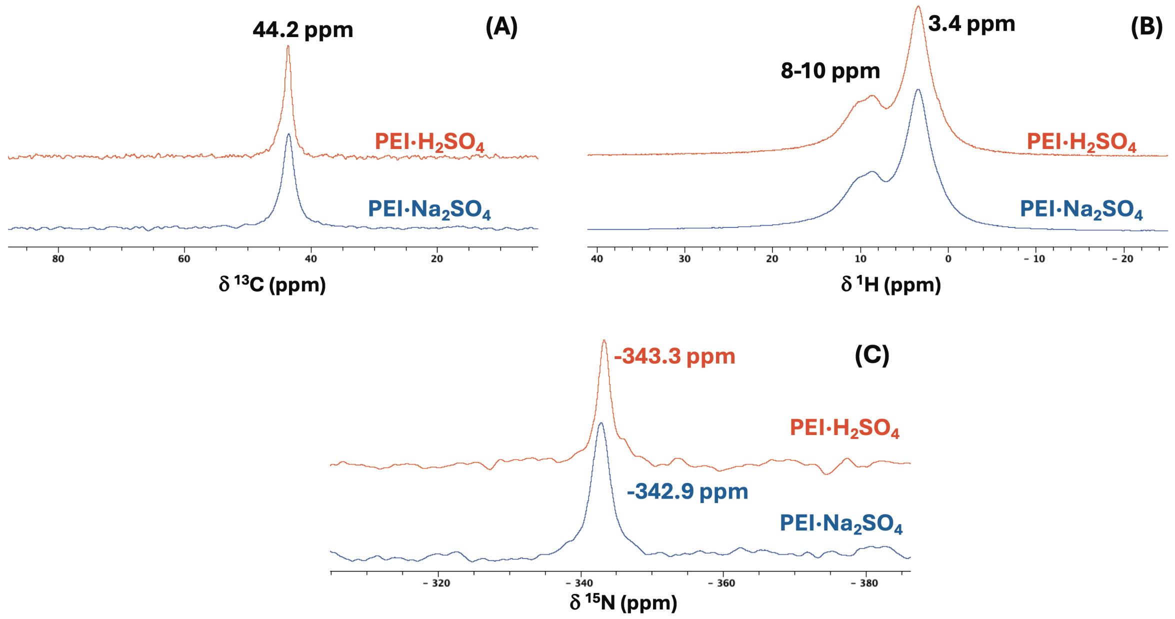

Figure S4 (II). Solid-state NMR results for the PEI·H_2SO_4 and PEI·Na_2SO_4 samples. ^{13}C CP-MAS (@10 kHz, 150.91 MHz) (A), ^{1}H-MAS (@60 kHz, 499.70 MHz) and ^{15}N CP-MAS (@10 kHz, 60.80 MHz) (C) spectra.

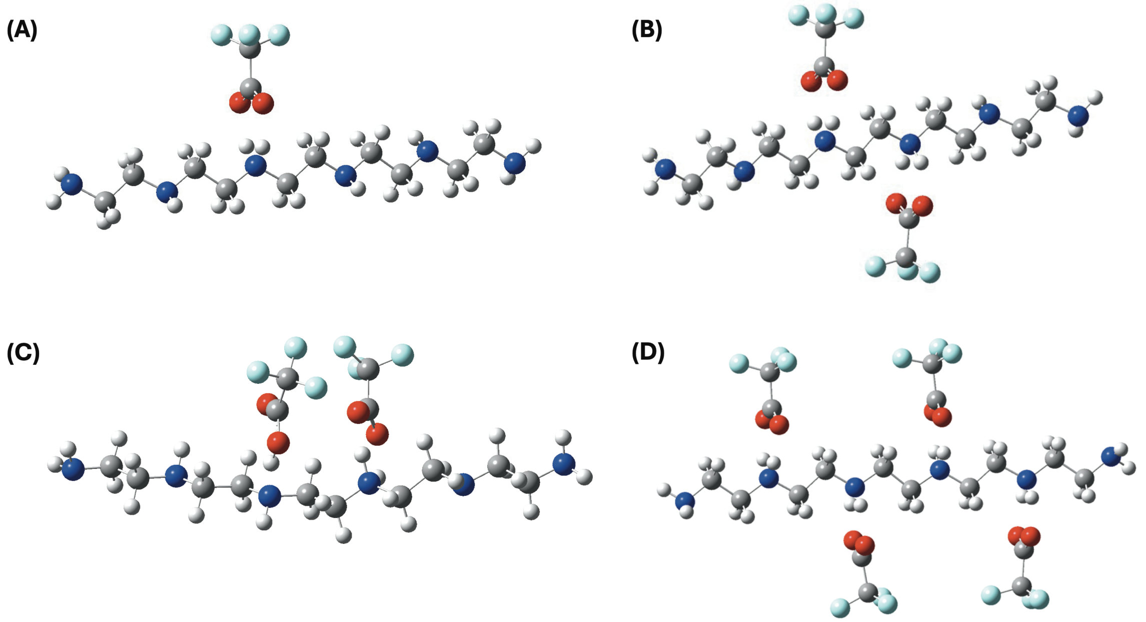

Figure S5. Optimized geometries of the L-PEI hexamer models with trifluoroacetate as counterion at increasing protonation degrees: 25% (A), 50% (*trans*) (B), 50% (*cis*) (C), and 100% (D).

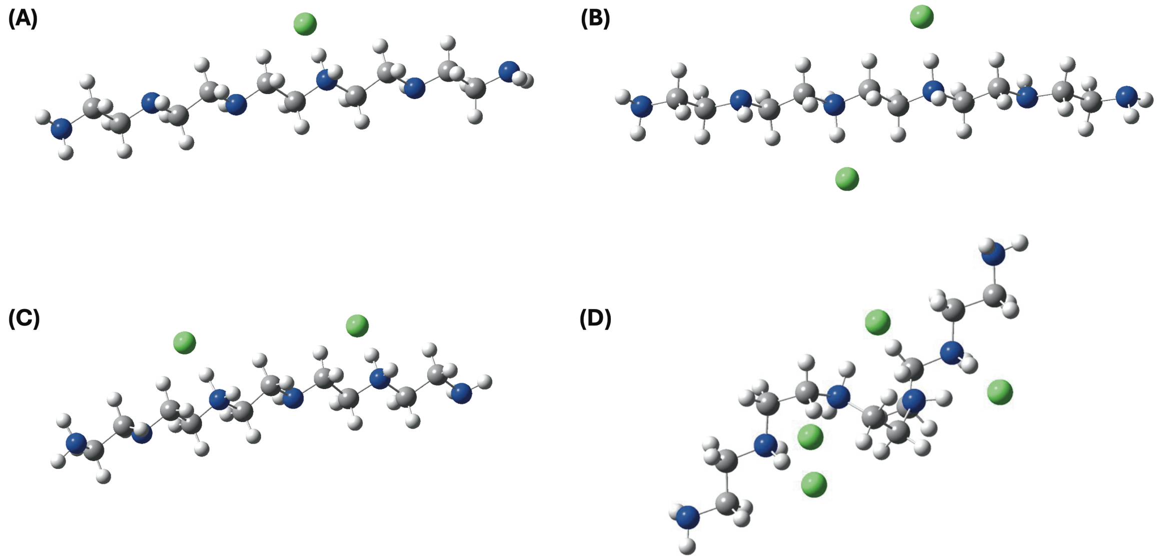

Figure S6. Optimized geometries of the L-PEI hexamer models with chloride as counterion at increasing protonation degrees: 25% (A), 50% (*trans*) (B), 50% (*cis*) (C) and 100% (D).

Table S2. Calculated ^{13}C values (relative to TMS) for free base and hydrochloride hexamers with 25% protonation state.

Atom	0% protonation state			25% protonation state		
	B3LYP	B3LYP-D3	ωB97X-D	B3LYP	B3LYP-D3	ωB97X-D
2	49.1757	49.1188	46.5432	48.6434	48.5961	45.8744
3	58.8237	58.8250	55.9730	58.6433	58.6582	56.0324
5	57.2053	57.1788	54.4401	50.9791	50.9465	48.5931
6	57.1961	57.1673	54.4296	52.9491	52.6042	50.4419
8	57.1959	57.1674	54.5263	52.9466	52.5952	50.4142
9	57.2418	57.2161	54.3551	51.0366	50.9913	48.7793
11	57.2063	57.1768	54.4582	56.9700	56.9320	54.2239
12	57.0536	57.0393	54.2998	56.6195	56.5983	54.0356
14	58.7920	58.8136	55.8665	58.9576	58.9483	56.0441
15	49.2549	49.2261	46.4811	48.9848	48.9537	46.4804

Table S3. Calculated ^{13}C values (relative to TMS) for hydrochloride hexamers with 50% protonation state (*cis* and *trans*).

Atom	50% protonation state (*cis*)			50% protonation state (*trans*)		
	B3LYP	B3LYP-D3	ωB97X-D	B3LYP	B3LYP-D3	ωB97X-D
2	43.6496	43.5372	41.4973	43.6496	43.5372	41.4973
3	54.4630	54.1257	51.7124	54.4629	54.1257	51.7124
5	52.7570	52.4599	50.3228	52.7570	52.4599	50.3228
6	51.1518	51.0863	48.8925	51.1518	51.0863	48.8925
8	51.4382	51.3495	49.1011	51.4382	51.3495	49.1011
9	52.8049	52.4594	50.2612	52.8049	52.4594	50.2612
11	53.1103	52.7576	50.5740	53.1103	52.7576	50.5740
12	50.8065	50.7806	48.4249	50.8066	50.7806	48.4249
14	58.5858	58.5811	55.9334	58.5859	58.5811	55.9334
15	48.7077	48.6504	46.0200	48.7077	48.6504	46.0200

Table S4. Calculated ^{13}C values (relative to TMS) for hydrochloride hexamers with 100% protonation state.

Atom	100% protonation state		
	B3LYP	B3LYP-D3	ωB97X-D
2	43.3392	43.2660	40.9636
3	56.0207	55.6713	53.8341
5	49.1494	48.6382	46.5221
6	48.6104	48.0639	46.1910
8	47.0504	46.5594	44.7385
9	48.8959	48.4870	46.6828
11	44.8527	44.4089	42.8689
12	48.6794	48.0547	45.3688
14	54.5744	54.0454	51.8282
15	42.3641	42.3611	40.5973

Table S5. Calculated ^{13}C values (relative to TMS) for trifluoroacetate hexamers with 25% and 100% protonation state.

Atom	25% protonation state			100% protonation state		
	B3LYP	B3LYP-D3	ωB97X-D	B3LYP	B3LYP-D3	ωB97X-D
2	49.0201	48.9293	46.6818	43.1399	43.2671	40.9190
3	58.3872	58.3549	55.2684	54.4749	53.9018	51.8328
5	49.6183	49.6848	47.2390	46.4439	45.5095	43.7878
6	53.4996	52.9232	50.8964	45.3687	44.9115	43.4653
8	53.4436	52.8498	50.6505	44.9399	45.1770	43.6306
9	49.4254	49.3930	47.3778	45.2789	44.8298	43.3834
11	56.6605	56.6260	54.0343	45.4022	45.2617	43.7431
12	56.7322	56.6978	53.8645	46.6297	45.1837	43.5413
14	58.8646	58.8508	55.5226	54.4876	53.8588	51.7984
15	49.1240	49.0885	46.7743	43.0682	43.1435	40.8992

Table S6. Calculated ^{13}C values (relative to TMS) for trifluoroacetate hexamers with 50% protonation state (*cis* and *trans*).

Atom	50% protonation state (*cis*)			50% protonation state (*trans*)		
	B3LYP	B3LYP-D3	ωB97X-D	B3LYP	B3LYP-D3	ωB97X-D
2	48.7946	48.8225	46.8719	48.6871	48.6576	45.9731
3	58.1841	58.2119	55.4137	58.3413	58.4281	55.4627
5	49.2981	49.3260	47.3042	50.0887	50.2917	47.9536
6	53.2585	53.2864	51.7024	52.7457	52.1992	50.1618
8	50.7811	50.8090	49.0112	45.6467	44.9763	43.2505
9	46.7298	46.7576	45.2438	45.7117	44.9758	43.2519
11	52.7717	52.7997	51.0001	52.7891	52.1990	50.1630
12	54.5133	54.5412	52.3397	50.1130	50.2923	47.9548
14	58.6633	58.6912	55.9652	58.3432	58.4281	55.4631
15	48.8790	48.9070	46.7622	48.6693	48.6577	45.9732

Table S7. Calculated ^{13}C values (relative to TMS) for dimers with 0% and 100% protonation state.

Atom	Chain	0% protonation state	100% protonation state

		B3LYP	B3LYP-D3	ωB97X-D	B3LYP	B3LYP-D3	ωB97X-D
2	A	49.2600	49.07993	46.7569	42.0642	42.0921	40.6589
3	A	58.8145	58.86773	56.269	54.8451	54.8729	53.3191
5	A	57.2791	57.80493	55.3819	47.7070	47.7349	46.2459
6	A	57.0561	57.07433	54.4273	47.2201	47.2480	46.4551
8	A	55.9188	55.84733	54.1649	48.5919	48.6199	47.2342
9	A	56.6744	55.77763	53.5279	50.2542	50.2821	49.5190
11	A	56.8620	56.24533	53.3717	50.2155	50.2435	49.5084
12	A	56.5209	56.60363	54.5414	46.8213	46.8492	45.2570
14	A	58.7858	58.77073	56.0889	55.0987	55.1265	53.1655
15	A	49.1148	49.06833	46.651	41.7630	41.7910	40.4020
46	B	49.2600	49.07993	46.7569	42.0604	42.0883	40.7268
47	B	58.8145	58.86773	56.269	54.1332	54.1611	52.4051
49	B	57.2791	57.80493	55.3819	49.5787	49.6066	48.3934
50	B	57.0561	57.07433	54.4273	49.7460	49.7739	48.7685
52	B	55.9188	55.84733	54.1649	50.1713	50.1992	49.5249
53	B	56.6744	55.77763	53.5279	46.3668	46.3948	45.4784
55	B	56.8620	56.24533	53.3717	47.7351	47.7630	46.4151
56	B	56.5209	56.60363	54.5414	47.7452	47.7732	46.1220
58	B	58.7858	58.77073	56.0889	56.2000	56.2280	54.0313
59	B	49.1148	49.06833	46.651	45.7029	45.7308	44.4773

Table S8. Calculated (relative to nitromethane) and experimental ^{15}N chemical shifts of the internal backbone nitrogen atoms for the free base and the fully protonated PEI salts.

	Counterion	Protonation (%)	δ calc (ppm)	δ exp (ppm)
Hexamer	-	0	-355.5	-345.0
Hexamer	Cl^-	100	-344.2	-333.4
Hexamer	$CF_3CO_2^-$	100	-337.5	-345.1

Table S9. Calculated ^{15}N values (relative to nitromethane) for trifluoroacetate hexamers with 50% protonation state (*cis* and *trans*).

Atom	50% protonation state (*cis*)	50% protonation state (*trans*)

	B3LYP	B3LYP-D3	ωB97X-D	B3LYP	B3LYP-D3	ωB97X-D
1	-382.2563	-382.1853	-382.7229	-382.6500	-382.5573	-384.3231
4	-358.1122	-358.0413	-361.4864	-357.9959	-358.0247	-362.4881
7	-337.1639	-337.0929	-338.0477	-337.4879	-337.9799	-340.4603
10	-348.9513	-348.8803	-352.2200	-337.5217	-337.9835	-340.4627
13	-357.7074	-357.6365	-361.0484	-358.0015	-358.0234	-362.4870
16	-382.4963	-382.4251	-382.9381	-382.6823	-382.5569	-384.3231

Table S10. Calculated ^{15}N values (relative to nitromethane) for hydrochloride hexamers with 50% protonation state (*cis* and *trans*).

Atom	50% protonation state (*cis*)			50% protonation state (*trans*)		
	B3LYP	B3LYP-D3	ωB97X-D	B3LYP	B3LYP-D3	ωB97X-D
1	-386.9684	-386.8638	-388.2050	-386.9683	-386.8638	-388.205
4	-336.8249	-337.2742	-340.3086	-336.8249	-337.2742	-340.3086
7	-362.8272	-363.0696	-367.2430	-362.8272	-363.0696	-367.243
10	-336.4186	-336.9705	-340.4885	-336.4186	-336.9705	-340.4885
13	-359.4011	-359.4321	-364.0145	-359.4011	-359.4321	-364.0145
16	-382.4326	-382.3466	-383.7473	-382.4324	-382.3466	-383.7473

XYZ coordinates of PEI derivatives

Unprotonated dimeric PEI (0%)

N	7.16037400	-4.48007600	-3.93504900
C	6.22296500	-3.36207000	-4.09556300
C	5.61213800	-2.98616700	-2.74525500
N	4.67190700	-1.87733700	-2.88810900
C	4.02966300	-1.49273200	-1.63255400
C	3.04866800	-0.34043700	-1.85157700
N	2.41284600	0.04567800	-0.59489200
C	1.49317600	1.16878100	-0.74007600
C	0.99150400	1.63166200	0.63222100
N	-0.26779600	2.37691100	0.51913200
C	-0.69189300	2.97481700	1.78721600
C	-2.01170200	3.73057600	1.63126700
N	-2.41284600	4.33533400	2.89842100
C	-3.63669300	5.12710500	2.80426000
C	-3.99532800	5.74105800	4.15717600
N	-5.21012200	6.55291400	4.03641500
H	6.64067900	-5.32681000	-3.70511800
H	7.62134000	-4.67104400	-4.82294900
H	6.79326400	-2.50601000	-4.48209200
H	5.40261300	-3.54516900	-4.81049300
H	5.05383200	-3.84795900	-2.35086200
H	6.42925300	-2.78889600	-2.02711600
H	5.17798200	-1.06930000	-3.25226600
H	3.48023800	-2.36719800	-1.25644600
H	4.74527600	-1.20672200	-0.83948800
H	3.59793500	0.53258200	-2.23583500
H	2.33072100	-0.62944900	-2.64185200
H	1.87943000	-0.75395300	-0.23825400
H	2.02947700	1.99159800	-1.23423000
H	0.61915100	0.94006700	-1.37923800
H	0.80175900	0.75184300	1.25955300
H	1.78540100	2.20740900	1.13996200
H	-0.14274900	3.12545100	-0.16398700
H	-0.82302600	2.15684100	2.51006100
H	0.06244500	3.65674700	2.21767600
H	-1.87962900	4.54348500	0.90259300
H	-2.77286200	3.04897900	1.20781200
H	-2.55623600	3.59165100	3.58223300
H	-3.47388800	5.93883800	2.08562400
H	-4.50071800	4.54285600	2.42560000
H	-4.06567900	4.93366400	4.91253000
H	-3.17331400	6.39298800	4.47232000
H	-6.01402900	5.94727000	3.87453200
H	-5.39305200	7.03281000	4.91561300
N	-7.16037400	4.48007600	-3.93504900
C	-6.22296500	3.36207000	-4.09556300

C	-5.61213800	2.98616700	-2.74525500
N	-4.67190700	1.87733700	-2.88810900
C	-4.02966300	1.49273200	-1.63255400
C	-3.04866800	0.34043700	-1.85157700
N	-2.41284600	-0.04567800	-0.59489200
C	-1.49317600	-1.16878100	-0.74007600
C	-0.99150400	-1.63166200	0.63222100
N	0.26779600	-2.37691100	0.51913200
C	0.69189300	-2.97481700	1.78721600
C	2.01170200	-3.73057600	1.63126700
N	2.41284600	-4.33533400	2.89842100
C	3.63669300	-5.12710500	2.80426000
C	3.99532800	-5.74105800	4.15717600
N	5.21012200	-6.55291400	4.03641500
H	-6.64067900	5.32681000	-3.70511800
H	-7.62134000	4.67104400	-4.82294900
H	-6.79326400	2.50601000	-4.48209200
H	-5.40261300	3.54516900	-4.81049300
H	-5.05383200	3.84795900	-2.35086200
H	-6.42925300	2.78889600	-2.02711600
H	-5.17798200	1.06930000	-3.25226600
H	-3.48023800	2.36719800	-1.25644600
H	-4.74527600	1.20672200	-0.83948800
H	-3.59793500	-0.53258200	-2.23583500
H	-2.33072100	0.62944900	-2.64185200
H	-1.87943000	0.75395300	-0.23825400
H	-2.02947700	-1.99159800	-1.23423000
H	-0.61915100	-0.94006700	-1.37923800
H	-0.80175900	-0.75184300	1.25955300
H	-1.78540100	-2.20740900	1.13996200
H	0.14274900	-3.12545100	-0.16398700
H	0.82302600	-2.15684100	2.51006100
H	-0.06244500	-3.65674700	2.21767600
H	1.87962900	-4.54348500	0.90259300
H	2.77286200	-3.04897900	1.20781200
H	2.55623600	-3.59165100	3.58223300
H	3.47388800	-5.93883800	2.08562400
H	4.50071800	-4.54285600	2.42560000
H	4.06567900	-4.93366400	4.91253000
H	3.17331400	-6.39298800	4.47232000
H	6.01402900	-5.94727000	3.87453200
H	5.39305200	-7.03281000	4.91561300

Protonated dimeric PEI (100%):

N	6.49532300	-4.07368200	-2.50078500
C	5.64430400	-3.05407900	-1.88185200

C	4.54911300	-3.77306700	-1.08845300
N	3.61174100	-2.78811900	-0.46318300
C	2.49983600	-3.40009200	0.32851400
C	1.82332900	-2.30315000	1.15239000
N	0.48324800	-2.72416800	1.62963400
C	-0.20475000	-1.62852500	2.35834700
C	-1.61445500	-2.05715500	2.77815500
N	-2.47161300	-0.82329600	3.00565100
C	-3.42005600	-0.50580500	1.87549600
C	-4.21985200	0.75641600	2.21520400
N	-5.11058300	1.09771100	1.07677000
C	-6.06429400	2.21253400	1.36029300
C	-6.99521800	2.43844400	0.16636200
N	-7.88669900	3.55618600	0.48810600
H	6.07029000	-4.40420400	-3.36553300
H	7.38905400	-3.66512800	-2.76274000
H	6.25117600	-2.46396400	-1.18671300
H	5.17902200	-2.34856900	-2.58764900
H	3.95301800	-4.42030500	-1.74024900
H	4.98875400	-4.38456200	-0.29693700
H	4.14786300	-2.10453600	0.13262400
H	1.80097300	-3.84598400	-0.38581800
H	2.90411400	-4.18657300	0.97028300
H	2.43114100	-2.03820100	2.02020900
H	1.69711500	-1.41692500	0.52750700
H	-0.09847600	-2.95885000	0.80840100
H	0.40072300	-1.37818700	3.23216500
H	-0.26022100	-0.75900900	1.70377100
H	-2.09958600	-2.64348600	1.99539300
H	-1.58183900	-2.65079000	3.69332200
H	-3.00531200	-0.94022900	3.87137400
H	-2.80545800	-0.36508200	0.98334300
H	-4.08761700	-1.35770300	1.71602900
H	-4.85972000	0.58383500	3.08797500
H	-3.55181500	1.59979800	2.41278500
H	-4.52180400	1.36339000	0.25239000
H	-6.65128100	1.94369400	2.24197300
H	-5.47814100	3.11019300	1.58484400
H	-6.39189900	2.58240600	-0.74438500
H	-7.59466900	1.53371900	0.02287000
H	-7.42473200	4.44138600	0.28696000
H	-8.71076800	3.52263700	-0.10683900
H	3.19421000	-2.18883700	-1.22820500
Cl	2.47227100	-1.00066600	-2.76064700
H	0.56619400	-3.59504100	2.36726600
Cl	0.63563100	-4.65690800	3.74371200

H	-1.85342700	0.03881900	3.13152600
Cl	-0.83387200	1.76429200	2.89596800
H	-5.67839700	0.20484500	0.81139900
Cl	-6.56472100	-1.43460100	0.51937800
N	8.15068900	3.52034600	-0.10341400
C	6.80216900	2.99402400	-0.31792300
C	5.99309600	3.22389600	0.96070700
N	4.62194800	2.63844000	0.82810500
C	3.72907400	2.91350900	1.99587100
C	2.44894400	2.08386900	1.98000800
N	1.46933000	2.44724000	0.88209300
C	1.29158100	1.37473800	-0.14628300
C	0.39425100	1.85701700	-1.28845100
N	0.02214200	0.68199300	-2.14271200
C	-0.72423100	1.07480100	-3.38570500
C	-1.59694000	-0.04496500	-3.95753800
N	-2.73590500	-0.39934800	-3.05212000
C	-3.92225200	-1.07094900	-3.68326200
C	-4.85793200	-1.54697200	-2.56662600
N	-6.09822100	-2.07086200	-3.13089300
H	8.72257600	2.83455100	0.38611200
H	8.60541400	3.69021400	-0.99680700
H	6.33337800	3.55404400	-1.13430400
H	6.76309500	1.92515700	-0.58611200
H	6.46892100	2.74158900	1.82027400
H	5.89225500	4.29346600	1.15819300
H	4.15240200	3.07592100	-0.01267400
H	4.27855100	2.64581200	2.90234400
H	3.52585700	3.98739700	2.00209700
H	2.69955300	1.02496700	1.88492800
H	1.91473100	2.22554200	2.92160000
H	0.54991900	2.59048000	1.35343300
H	2.27496800	1.07854200	-0.51754700
H	0.83584200	0.52718300	0.36679500
H	-0.54210100	2.29015100	-0.92138500
H	0.92492500	2.58741700	-1.90426900
H	0.89805300	0.12898500	-2.41206500
H	-1.34091500	1.94161800	-3.14004600
H	0.02523200	1.35772300	-4.12939500
H	-2.01617300	0.30949000	-4.90320200
H	-1.02009900	-0.95436900	-4.14859700
H	-2.36640700	-1.03216300	-2.30324000
H	-4.42294700	-0.34698600	-4.33091900
H	-3.55186400	-1.90067500	-4.29479700
H	-4.31618900	-2.25206000	-1.91646100
H	-5.11892000	-0.69283400	-1.93566500

H	-5.97992200	-3.05254800	-3.37749400
H	-6.79263600	-2.05427400	-2.38302500
H	4.71212000	1.59381200	0.71903800
Cl	4.87917300	-0.43126600	1.14698900
H	1.79366000	3.32021700	0.36334500
Cl	2.87621500	4.38459800	-0.96665300
H	-0.52242400	-0.00353500	-1.57605800
Cl	-1.24495400	-1.95368500	-0.85033000
H	-3.05995700	0.46330500	-2.52862300
Cl	-3.26877000	2.06878400	-1.29471700

PEI·FB:

N	9.20289200	0.31919100	-0.01356300
C	7.98181600	-0.49108000	0.06702300
C	6.74677500	0.40113000	-0.06213700
N	5.52082500	-0.39011300	0.02063600
C	4.30324300	0.41273400	-0.06745600
C	3.05983000	-0.46994400	0.04810100
N	1.84269700	0.33376900	-0.03799800
C	0.61624600	-0.45601200	0.04353600
C	-0.61630000	0.44106600	-0.07710200
N	-1.84355400	-0.34737000	0.00648000
C	-3.05910700	0.45896300	-0.07880300
C	-4.30483800	-0.42021900	0.03760700
N	-5.51950100	0.38697200	-0.04167400
C	-6.74768300	-0.39876600	0.03558400
C	-7.97870300	0.50158000	-0.06576400
N	-9.20182700	-0.30438400	-0.00003600
H	9.28515000	0.89182600	0.82614500
H	10.01913900	-0.28987600	-0.01421100
H	8.00064500	-1.20076700	-0.77158000
H	7.88451300	-1.09079900	0.98809800
H	6.73513300	1.12169600	0.76876700
H	6.83143700	0.99523300	-0.99029300
H	5.51914800	-1.06374400	-0.74598400
H	4.31710800	1.12942000	0.76678300
H	4.23256200	1.01191700	-0.99381400
H	3.04492500	-1.18564000	-0.78694400
H	3.13073200	-1.07013700	0.97391300
H	1.84518600	1.00758400	0.72830100
H	0.62606400	-1.17205600	-0.79125900
H	0.53466400	-1.05501200	0.96922500
H	-0.62488700	1.15802200	0.75700800
H	-0.53520000	1.03914800	-1.00334400
H	-1.84813300	-1.02112500	-0.75988400

H	-3.04183300	1.17465500	0.75632200
H	-3.12918600	1.05941400	-1.00439400
H	-4.32486900	-1.13365300	-0.79891200
H	-4.23277100	-1.02321100	0.96187700
H	-5.51296400	1.05964000	0.72558500
H	-6.76371700	-1.10767300	-0.80047100
H	-6.81339600	-1.00736000	0.96169000
H	-7.91472400	1.28765800	0.71229100
H	-7.95486700	1.01643800	-1.03235400
H	-9.31415400	-0.68235500	0.94006000
H	-10.01408900	0.28758200	-0.16308200

PEI·HCl (25% of protonation):

N	8.94899100	-0.80862000	0.38117900
C	7.72780900	-0.86971900	-0.42805000
C	6.52102600	-0.45676400	0.41554500
N	5.28561300	-0.54975500	-0.36720500
C	4.09476500	-0.15703900	0.38054300
C	2.85323600	-0.53017800	-0.43106000
N	1.61604100	-0.05187300	0.24617700
C	0.34243100	-0.44204000	-0.41912500
C	-0.86208400	0.04411300	0.38840300
N	-2.08459100	-0.28820500	-0.33717000
C	-3.30451800	-0.04063800	0.43581200
C	-4.54238700	-0.41692600	-0.37917800
N	-5.75927600	-0.17314800	0.38828700
C	-6.98488100	-0.45300700	-0.35506900
C	-8.21935200	-0.14594500	0.49221200
N	-9.43821900	-0.41750200	-0.27568100
H	8.95360500	-1.57584700	1.05234900
H	9.76398900	-0.95117000	-0.21202900
H	7.84434200	-0.15927300	-1.25754800
H	7.51934400	-1.85495600	-0.87970900
H	6.42645300	-1.14280300	1.26974100
H	6.70472200	0.54747400	0.83398100
H	5.37089700	0.06870300	-1.17455400
H	4.09170500	-0.71720100	1.32649600
H	4.05340500	0.91589700	0.63267900
H	2.87158100	-0.03078800	-1.40490400
H	2.79962400	-1.61171900	-0.58984600
H	1.60967400	-0.35086300	1.22492600
H	0.36002600	0.03872500	-1.40238600
H	0.30896500	-1.52719300	-0.55770400
H	-0.88940000	-0.48374100	1.35255100
H	-0.74184400	1.11900200	0.60451000

H	-2.11968500	0.28594500	-1.18021300
H	-3.25854300	-0.66758800	1.33822100
H	-3.40356700	1.00351700	0.77879100
H	-4.58661500	0.21197500	-1.27981000
H	-4.43791500	-1.46065000	-0.73053300
H	-5.74221500	-0.75670000	1.22508100
H	-7.00670700	0.18482100	-1.24627200
H	-7.03963900	-1.50010700	-0.71902700
H	-8.15259600	-0.70699900	1.44524700
H	-8.20490900	0.91739500	0.75446400
H	-9.55033100	-1.42290400	-0.40092700
H	-10.25346100	-0.10714400	0.24933000
H	1.65851700	1.10280400	0.21463900
Cl	1.72437600	2.79199800	-0.12452200

PEI.HCl (50% of protonation) *cis:*

N	8.65446200	-1.70596900	-0.10329100
C	7.52942600	-1.04195800	0.56160500
C	6.25943700	-1.33004200	-0.24145100
N	5.08968800	-0.61903200	0.34555900
C	3.79721000	-0.85400200	-0.35208500
C	2.64876300	-0.15233300	0.37618200
N	1.41735200	-0.35482600	-0.38922900
C	0.19929200	0.00817000	0.33702300
C	-1.01148600	-0.46521900	-0.47010500
N	-2.27947400	-0.11777400	0.22444300
C	-3.51879500	-0.56702900	-0.46893600
C	-4.75489400	-0.24551100	0.37221700
N	-5.95061800	-0.60507100	-0.38314200
C	-7.17686900	-0.55425500	0.41599700
C	-8.39307400	-0.92187200	-0.43331700
N	-9.60368700	-0.89775600	0.39191100
H	8.94130300	-1.15459600	-0.91134500
H	9.45832600	-1.72095300	0.52077300
H	7.42741600	-1.47465600	1.56601400
H	7.63415000	0.04758300	0.67655100
H	6.36406500	-0.94642200	-1.26128800
H	6.06248400	-2.40571400	-0.28866100
H	5.00364700	-0.84386800	1.34026400
H	3.91959200	-0.43814900	-1.35724100
H	3.60521300	-1.92836000	-0.43670400
H	2.51080100	-0.61350500	1.36411500
H	2.90985300	0.90561000	0.53690700
H	1.47008400	0.21953200	-1.23092100
H	0.21701300	-0.51557800	1.30323400

H	0.09914100	1.08516600	0.54520600
H	-1.04761900	0.04661500	-1.43716300
H	-0.96248500	-1.54481200	-0.64519500
H	-2.26542900	-0.47003900	1.18515800
H	-3.54828300	-0.01583200	-1.41428600
H	-3.46119800	-1.63822300	-0.68658100
H	-4.73975800	-0.85473200	1.28711700
H	-4.71703700	0.81305000	0.68031700
H	-6.04493400	0.04823300	-1.16141100
H	-7.09127100	-1.27839400	1.23444600
H	-7.34040100	0.43588500	0.88473100
H	-8.43354400	-0.25320400	-1.31523600
H	-8.25560800	-1.93874100	-0.81715600
H	-9.83608300	0.06440000	0.63547700
H	-10.39407900	-1.25026600	-0.14426800
H	5.31382000	0.51858400	0.24196600
Cl	5.64513100	2.14082800	-0.18074400
H	-2.33318400	1.04774500	0.25880800
Cl	-2.42360800	2.73569800	0.04308700

PEI·HCl (50% of protonation) *trans*:

N	9.19708700	-0.49743400	0.35460900
C	7.97566600	-0.55245200	-0.45422800
C	6.76850300	-0.16139300	0.39921800
N	5.53254000	-0.24011900	-0.38480100
C	4.33836700	0.11499000	0.37286300
C	3.10263400	-0.26905500	-0.44280700
N	1.85791300	0.16705700	0.25634000
C	0.59517100	-0.24508700	-0.41367200
C	-0.59589200	0.35915700	0.33646500
C	-3.10401000	0.35996600	0.38491300
C	-4.34197600	-0.08950900	-0.39299200
N	-5.53246800	0.25973900	0.37215100
C	-6.77921700	0.08442600	-0.37596400
C	-7.98403900	0.46383400	0.48462900
N	-9.21827100	0.30325500	-0.28795600
H	9.20944700	-1.27814600	1.00993900
H	10.01248900	-0.62076100	-0.24227900
H	8.08652300	0.17310900	-1.27142600
H	7.77214500	-1.53074400	-0.92244300
H	6.67573300	-0.86651100	1.23757200
H	6.94970000	0.83398600	0.83934700
H	5.61236400	0.39722400	-1.17760200
H	4.34857400	-0.46334300	1.30726600
H	4.27743000	1.18194300	0.64924000
H	3.10191000	0.25136900	-1.40562900

H	3.06903200	-1.34745000	-0.62137500
H	1.86583900	-0.16637100	1.22446700
H	0.64878700	0.15611700	-1.43086400
H	0.51466200	-1.33607100	-0.44673900
H	-0.63606400	-0.02710000	1.36005800
H	-0.52670700	1.45108200	0.35478400
H	-3.07704100	-0.11707900	1.36962600
H	-3.09139800	1.44555400	0.51573900
H	-4.38560000	0.45038600	-1.34899000
H	-4.25418300	-1.16492600	-0.62757700
H	-5.56673400	-0.33041200	1.20380900
H	-6.75640900	0.74118600	-1.25306700
H	-6.90762300	-0.94811300	-0.75580000
H	-7.95787000	-0.12379800	1.42310900
H	-7.88899100	1.51669700	0.77154300
H	-9.41463000	-0.68738600	-0.42556800
H	-10.00627600	0.67627000	0.23751300
H	1.84956600	1.31290900	0.25219300
Cl	1.67720800	3.01863800	-0.06843200
N	-1.86026400	-0.08032600	-0.31368200
H	-1.88033600	0.22717900	-1.29018600
H	-1.84040700	-1.22274100	-0.27965800
Cl	-1.64726200	-2.92208100	0.09053900

PEI·HCl (100% of protonation):

N	7.06037200	-1.26242800	-1.21889300
C	6.36085700	-0.62007200	-0.10529500
C	4.96955500	-1.24767800	-0.00784700
N	4.18854600	-0.65524100	1.13441000
C	2.78507800	-1.20505000	1.27389300
C	1.73597400	-0.16030100	1.64784600
N	1.50238600	0.81934600	0.53238400
C	0.19413200	1.57267600	0.61370800
C	-0.59662300	1.55848900	-0.69035900
N	-1.00891000	0.16248100	-1.07697700
C	-2.13597100	0.09289700	-2.07710800
C	-3.22813400	-0.89788000	-1.67174800
N	-3.93496900	-0.48054900	-0.42301400
C	-5.27195800	-1.11164100	-0.22294900
C	-5.90822400	-0.61204400	1.07736000
N	-7.20462400	-1.27373600	1.23959000
H	6.69964200	-0.90739100	-2.10340700
H	8.04670600	-1.01388600	-1.19465600
H	6.92306600	-0.83326800	0.81391000
H	6.26245400	0.47444300	-0.17931500

H	4.37680600	-1.05766100	-0.90815200
H	5.04077900	-2.32848000	0.14026100
H	4.70148000	-0.80040800	2.00859200
H	2.51995100	-1.66091200	0.31652400
H	2.80027800	-1.98368200	2.04012300
H	0.79425800	-0.68903200	1.83254800
H	2.01090700	0.41536600	2.53630600
H	2.30955800	1.50633300	0.55454700
H	-0.40361300	1.13297000	1.41510100
H	0.42558600	2.60832100	0.87374900
H	-1.51349400	2.14406800	-0.54448400
H	-0.02382400	1.97231300	-1.52499800
H	-0.16320400	-0.32185700	-1.48295200
H	-2.57054200	1.09100000	-2.17102700
H	-1.70658500	-0.21632000	-3.03334800
H	-3.96383800	-0.92941700	-2.48018600
H	-2.83278500	-1.90633300	-1.51657600
H	-3.31848200	-0.72819400	0.38696000
H	-5.90610200	-0.84781400	-1.07297600
H	-5.13753600	-2.19911200	-0.20724500
H	-5.20601600	-0.77068200	1.91279700
H	-6.06632700	0.46781600	0.99113600
H	-7.08153800	-2.18952300	1.66777600
H	-7.78932400	-0.73704200	1.87529300
H	4.17619700	0.42672600	1.02887300
Cl	4.08436300	2.33447400	1.02051500
H	1.58898200	0.30714100	-0.39213900
Cl	1.73146000	-0.81359400	-2.07674800
H	-1.24499400	-0.38283600	-0.19823800
Cl	-1.64143000	-1.42772600	1.48774300
H	-4.04097600	0.60596800	-0.42003400
Cl	-4.01881500	2.49307600	-0.50811400

PEI·TFA (25% of protonation):

N	-8.50909900	-1.82167300	-0.57532800
C	-7.23271900	-2.46705100	-0.25439100
C	-6.11824600	-1.42158600	-0.19722100
N	-4.83550100	-2.05051100	0.13028400
C	-3.71626400	-1.11480100	0.15018300
C	-2.40945600	-1.90683900	0.25026700
N	-1.23016700	-0.99881500	0.24984400
C	0.09676200	-1.66956700	0.32104800
C	1.23461100	-0.64527900	0.29521900
N	2.51030400	-1.35229300	0.33012800
C	3.66412100	-0.48358500	0.08110100

C	4.96033600	-1.29484400	0.08546900
N	6.11009800	-0.42655400	-0.14340200
C	7.39119100	-1.12633900	-0.10330100
C	8.55022400	-0.15218600	-0.31418700
N	9.82844700	-0.86765500	-0.25289800
H	-8.49882200	-1.50556600	-1.54446700
H	-9.26518500	-2.50046900	-0.51091700
H	-7.33799600	-2.93365100	0.73491400
H	-6.92717900	-3.26646400	-0.95130000
H	-6.01350900	-0.94904700	-1.18372400
H	-6.41178900	-0.62379900	0.50638500
H	-4.91756500	-2.48501500	1.04995100
H	-3.71819500	-0.55112600	-0.78988000
H	-3.76370300	-0.36677000	0.96110200
H	-2.37567000	-2.49847800	1.17280400
H	-2.32205200	-2.59431900	-0.59570300
H	-1.27077000	-0.31784900	-0.56751900
H	0.12141200	-2.26546200	1.24115100
H	0.18865700	-2.35338300	-0.52745100
H	1.17797900	-0.07705900	-0.64040200
H	1.09881400	0.08627900	1.11128000
H	2.62496300	-1.78076600	1.24908600
H	3.52416500	-0.02412300	-0.90724300
H	3.75611800	0.34411700	0.80536100
H	5.09080900	-1.76516000	1.07096500
H	4.87041400	-2.12005800	-0.64570600
H	6.00767500	0.02095400	-1.05448800
H	7.50409900	-1.59393700	0.88178200
H	7.45947600	-1.94640100	-0.84801900
H	8.38858700	0.40208200	-1.25947900
H	8.52995000	0.59047500	0.49057700
H	9.93420800	-1.45540100	-1.07905800
H	10.59790400	-0.20151900	-0.28263900
H	-1.32272500	-0.24082400	0.98975600
F	-0.27312700	3.95868400	-0.14480500
C	-1.52948500	3.47028200	-0.01125900
C	-1.47956200	1.92188100	0.08707600
O	-1.48094600	1.44298500	1.25169900
O	-1.40066800	1.31721800	-1.01606800
F	-2.07115300	4.03439000	1.08217900
F	-2.24069700	3.87949500	-1.07877300

PEI·TFA (50% of protonation) *cis:*

N	8.96880800	-1.07222500	0.24464000
C	7.76160300	-1.75272100	0.71811500

C	6.52235200	-1.10803900	0.09784900
N	5.30667900	-1.79957000	0.53423000
C	4.08221500	-1.22612900	-0.01342200
C	2.92669300	-2.19854800	0.24124800
N	1.64593300	-1.66093800	-0.29453500
C	0.44960200	-2.51845400	-0.07210000
C	-0.75420700	-2.01452200	-0.89205200
N	-1.98841600	-2.07832800	-0.09255800
C	-3.18007500	-1.63128400	-0.84259800
C	-4.42599700	-1.67663900	0.04162200
N	-5.58112600	-1.19497600	-0.70446500
C	-6.83403900	-1.27433300	0.04419900
C	-8.00321800	-0.76418200	-0.79770500
N	-9.25277800	-0.87048800	-0.03828500
H	9.02120900	-0.13729100	0.64704400
H	9.79646700	-1.56713100	0.57061300
H	7.80659100	-2.79962900	0.39824900
H	7.64982500	-1.76324400	1.82050800
H	6.50920000	-0.02712400	0.33866200
H	6.59566200	-1.18533200	-0.99254100
H	3.82775300	-0.22983500	0.38716700
H	4.21239500	-1.10238400	-1.09502600
H	3.13488400	-3.16294300	-0.23156300
H	2.78629500	-2.36852100	1.31481700
H	1.46248800	-0.65253500	0.07246000
H	0.71285600	-3.54969400	-0.32859700
H	0.22460900	-2.47700700	0.99742200
H	-0.60443400	-0.96741100	-1.16840600
H	-0.83847500	-2.58123300	-1.83159200
H	-2.14096600	-3.03531200	0.23056100
H	-2.98772400	-0.59862200	-1.14897300
H	-3.34517800	-2.23523800	-1.74739400
H	-4.62933400	-2.71455300	0.34325600
H	-4.23144000	-1.10977800	0.97179000
H	-5.41489300	-0.22280100	-0.96577900
H	-7.02234700	-2.32281500	0.30344200
H	-6.79836800	-0.71534500	1.00139100
H	-7.77464700	0.26052800	-1.14961600
H	-8.08696800	-1.39089400	-1.69220800
H	-9.25816700	-0.18173100	0.71332200
H	-10.04095500	-0.63534900	-0.63842900
H	1.73755500	-1.37746800	-1.29837000
F	0.64488700	3.33761000	-0.96172400
C	1.31221200	2.55075300	-1.81902000
C	1.38390700	1.07916000	-1.33521400
O	1.56620800	0.21211600	-2.22036700

O	1.29092200	0.91023000	-0.08098900
F	0.71679800	2.64565600	-3.02144500
F	2.56808200	3.04467700	-1.94278500
F	0.38630500	1.64538800	2.71761100
C	-0.77957400	2.02923100	2.17757100
C	-1.37760200	0.95029400	1.25039000
O	-1.21608000	-0.26316300	1.76152700
O	-1.97497400	1.23299000	0.23548300
F	-1.65157600	2.24849900	3.19259700
F	-0.60362100	3.17748400	1.52630500
H	5.26036300	-1.76172200	1.55277800
H	-1.63766600	-0.95065200	1.12825500

PEI·TFA (50% of protonation) *trans:*

N	-9.04941000	-1.73535500	0.39021400
C	-7.69208300	-2.22666400	0.14410300
C	-6.70096800	-1.06345700	0.18677000
N	-5.33448300	-1.54212300	-0.03304400
C	-4.33146600	-0.48687900	-0.00130200
C	-2.94036700	-1.12599800	0.02814100
N	-1.87993400	-0.07975200	0.02574200
C	-0.48338200	-0.59064000	0.04844300
C	0.49608300	0.59234400	0.04234500
N	1.89335700	0.08216800	0.03039100
C	2.95307100	1.12928600	0.01620400
C	4.34491000	0.49124200	0.00187400
N	5.34688300	1.54704000	-0.04366500
C	6.71275800	1.07393600	0.19185400
C	7.70270000	2.23758800	0.13641200
N	9.05903900	1.75177600	0.39825600
H	-9.37974600	-1.20582300	-0.41544300
H	-9.68882400	-2.52046800	0.49397300
H	-7.43049100	-2.93488400	0.93764300
H	-7.57281200	-2.76823900	-0.81503500
H	-7.00644500	-0.29078400	-0.54606000
H	-6.75267700	-0.59537400	1.17587500
H	-4.39704700	0.22803400	-0.84254400
H	-4.47536100	0.09867800	0.91455100
H	-2.82398500	-1.74349700	0.92262900
H	-2.77899100	-1.76352200	-0.84833500
H	-1.99973700	0.61736900	-0.77025600
H	-0.34430400	-1.20796900	0.93998000
H	-0.32529400	-1.22667100	-0.82685200
H	0.34121300	1.21636600	-0.84205800
H	0.35320000	1.22161700	0.92488600

H	2.03081100	-0.62050600	0.80958200
H	2.79274200	1.74935600	-0.87278900
H	2.83416400	1.76376700	0.89843500
H	4.48664200	-0.07865300	0.92795000
H	4.41400700	-0.23749000	-0.82700200
H	5.31057200	2.00151600	-0.95622300
H	6.75915800	0.62198700	1.18869800
H	7.02356900	0.28992400	-0.52650000
H	7.58845200	2.76379900	-0.83184400
H	7.43570500	2.95797800	0.91710300
H	9.39494900	1.21037700	-0.39713700
H	9.69695900	2.53910600	0.49404500
H	-2.01800100	0.64084300	0.79352700
F	-0.91098400	4.86307600	-0.18395000
C	-2.16017500	4.38738800	-0.11266900
C	-2.12062200	2.85205000	-0.05952800
O	-2.08379400	2.33207800	1.10301100
O	-2.06454000	2.26284800	-1.18714900
F	-2.74007100	4.91535800	0.97915900
F	-2.83183700	4.83199900	-1.18872700
F	2.81834800	-4.84772600	-1.18615300
C	2.14385300	-4.39710000	-0.11432700
C	2.12355500	-2.84688400	-0.04962500
O	2.08151700	-2.26160200	-1.16753100
O	2.08988300	-2.34260100	1.10225400
F	0.86999800	-4.85456900	-0.20848500
F	2.69348000	-4.94506400	0.98136900
H	-5.29396100	-2.01297300	-0.93709200
H	2.01524100	-0.63086200	-0.75471300

PEI·TFA (100% of protonation):

N	0	8.44575900	3.79995100	-0.01493800
C	0	7.65514400	2.57002000	-0.00406500
C	0	6.17204000	2.95176800	-0.06113300
N	0	5.31544300	1.73224700	-0.05241800
C	0	3.85236000	1.97797800	-0.10463300
C	0	3.09667200	0.64047100	-0.10069500
N	0	1.64030000	0.92908400	-0.14408700
C	0	0.73223400	-0.24384600	-0.12386900
C	0	-0.72327000	0.25477600	-0.09714800
N	0	-1.62900200	-0.92043300	-0.05357000
C	0	-3.08658000	-0.63845100	-0.05730000
C	0	-3.83349600	-1.98081300	-0.05744500
N	0	-5.29804500	-1.74411900	-0.01937500
C	0	-6.14852300	-2.96807100	-0.02903000

C	0	-7.63390100	-2.59263000	0.01004900
N	0	-8.41890800	-3.82611700	-0.00105900
H	0	8.49171600	4.20091500	0.92001900
H	0	9.40425300	3.59631700	-0.28667600
H	0	7.90424500	1.98699200	-0.89750100
H	0	7.83093600	1.91098900	0.86247600
H	0	5.89070000	3.56592900	0.80140600
H	0	5.96458900	3.52394000	-0.96923700
H	0	3.56818800	2.58296500	0.76195000
H	0	3.61994100	2.55490000	-1.00468300
H	0	3.37386700	0.02917300	-0.96414100
H	0	3.32312800	0.06789500	0.80297400
H	0	1.34677100	1.64261500	0.61308000
H	0	0.91830600	-0.86497000	-1.00430800
H	0	0.95107600	-0.84392800	0.76440400
H	0	-0.90539800	0.88696900	0.77631000
H	0	-0.95006600	0.84253200	-0.99194400
H	0	-1.35069100	-1.63450500	-0.81324500
H	0	-3.34615600	-0.04663500	0.82512200
H	0	-3.33907600	-0.04933200	-0.94315600
H	0	-3.59271300	-2.55847700	-0.95520000
H	0	-3.54992900	-2.58206400	0.81193300
H	0	-5.54267300	-1.08479900	0.78365000
H	0	-5.92908900	-3.54445400	-0.93165200
H	0	-5.87308900	-3.57580100	0.83994900
H	0	-7.82179300	-1.92864100	0.87019700
H	0	-7.87599000	-2.01651500	-0.88974900
H	0	-8.47495600	-4.21991800	0.93638300
H	0	-9.37476500	-3.62865400	-0.28631800
H	0	1.40881600	1.58905200	-0.94059000
F	-1	-1.50646100	4.83946000	-0.03845500
C	-1	-0.18091700	5.02565800	-0.04857700
C	-1	0.50482500	3.65140400	-0.10185400
O	-1	0.86749400	3.19297000	-1.23390300
O	-1	0.67215700	3.02766100	0.99577000
F	-1	0.15731700	5.76424400	-1.11965500
F	-1	0.18692100	5.68535300	1.06327200
F	-1	-0.46851700	-5.90544400	-0.33497900
C	-1	0.40359300	-4.91234700	-0.12279800
C	-1	-0.36946900	-3.58495200	-0.07754900
O	-1	-0.71272800	-3.11610100	1.05628300
O	-1	-0.63933000	-3.01622600	-1.18459900
F	-1	1.04667400	-5.12434600	1.03850500
F	-1	1.30706200	-4.88984000	-1.11775900
H	0	5.54798800	1.07477900	0.75796100
H	0	-1.36689700	-1.57566600	0.74028100

F	0	5.42848500	-3.33789900	0.08151600
C	0	6.53654000	-2.56970500	0.20111000
C	0	6.13566000	-1.07362200	0.11152900
O	0	5.92550400	-0.49128800	1.21160300
O	0	6.01331800	-0.61183100	-1.05391900
F	0	7.11882900	-2.86820800	1.37391500
F	0	7.38492100	-2.91582000	-0.78257700
F	0	-8.12760900	2.39379900	-0.01611300
C	0	-6.78646400	2.47110100	0.14543200
C	0	-6.17806300	1.04496000	0.09301700
O	0	-5.99827000	0.57542200	-1.06279200
O	0	-5.96298200	0.49056600	1.20561900
F	0	-6.29872400	3.25173400	-0.83527400
F	0	-6.54816000	3.08120400	1.31751800
H	0	5.59746200	1.03754000	-0.80450900
H	0	-5.57575900	-1.05315300	-0.77905700

TD-NMR Fittings:

The MSE decays were fitted over the range t = 0–1 ms (n = 2499 points per curve). In addition to the parameters in Equation 2, a constant baseline offset (y_0) was included in the fit. The three-component model (Abragam function + two exponentials, k = 8 free parameters) was compared against a reduced two-component model (Abragam function + one exponential, k = 6; for the 87 kDa PEI·HCl sample the Abragam damping parameter b had to be fixed, as the fit was otherwise numerically unstable and converged toward a pure exponential, yielding k = 5). Model selection was assessed via the reduced χ^2, an extra-sum-of-squares F-test for nested models, and the corrected Akaike Information Criterion (AICc). For all samples, the three-component model was favored (ΔAICc $\geq$ 10); the improvement was decisive for the PEI·HCl sample (ΔAICc = 858.6, $F(3,2491)$ = 343, $p < 0.0001$), where the two-component model additionally required fixing b to achieve convergence and left pronounced systematic structure in the residuals. For the PEI·FB sample the improvement was more modest (ΔAICc = 10.2, $F(2,2491)$ = 7.09, $p < 0.001$), consistent with a smaller interfacial fraction and less-resolved domains in this material. The information has been added in the Supporting Information section.

Sample	***Model***	***k***	***RSS***	***χ^2 reduced***	***AICc***	***ΔAICc***
87 kDa PEI·FB	*Abragam+1exp+y0*	*6*	*1.06556*	*4.274E-4*	*−19380.5*	*10.2*
87 kDa PEI·FB	*Abragam+2exp+y0*	*8*	*1.05953*	*4.253E-4*	*−19390.7*	*0*
87 kDa PEI·HCl	*Abragam+1exp+y0 (w fixed)*	*5*	*1.48864*	*5.969E-4*	*−18547.0*	*858.6*
87 kDa PEI·HCl	*Abragam+2exp+y0*	*8*	*1.05326*	*4.228E-4*	*−19405.6*	*0*

Residuals for 87 kDa PEI·HCl Abragam + 1 exponencial Fit:

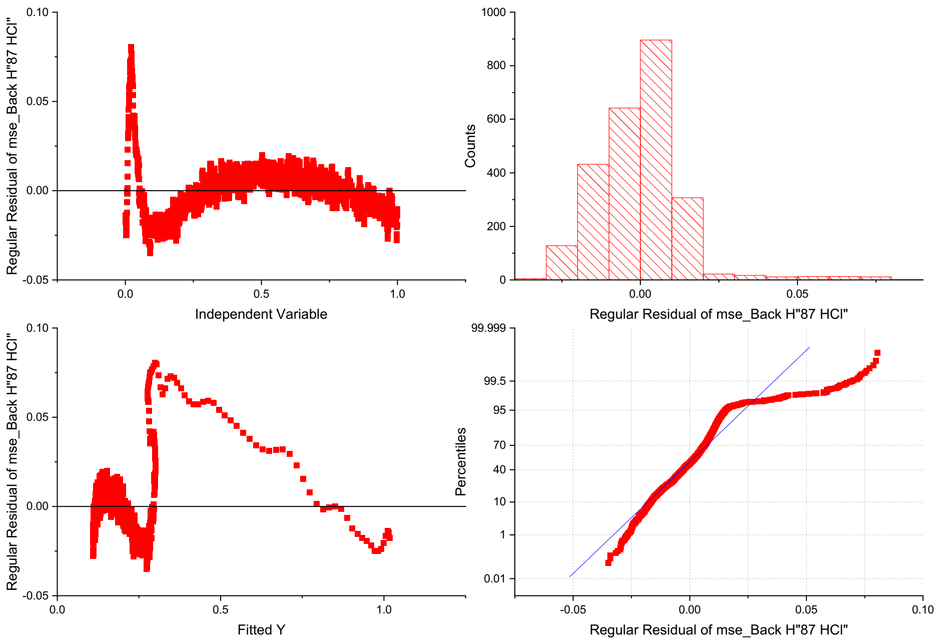

Residuals for 87 kDa PEI·HCl Abragam + 2 exponencial Fit:

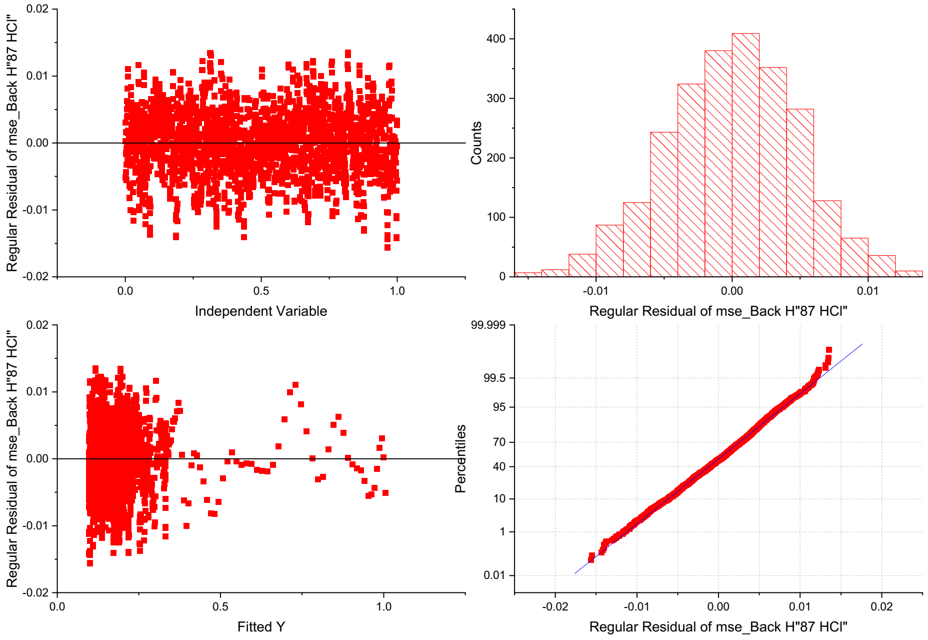

Residuals for 87 kDa PEI·FB Abragam + 1 exponencial Fit:

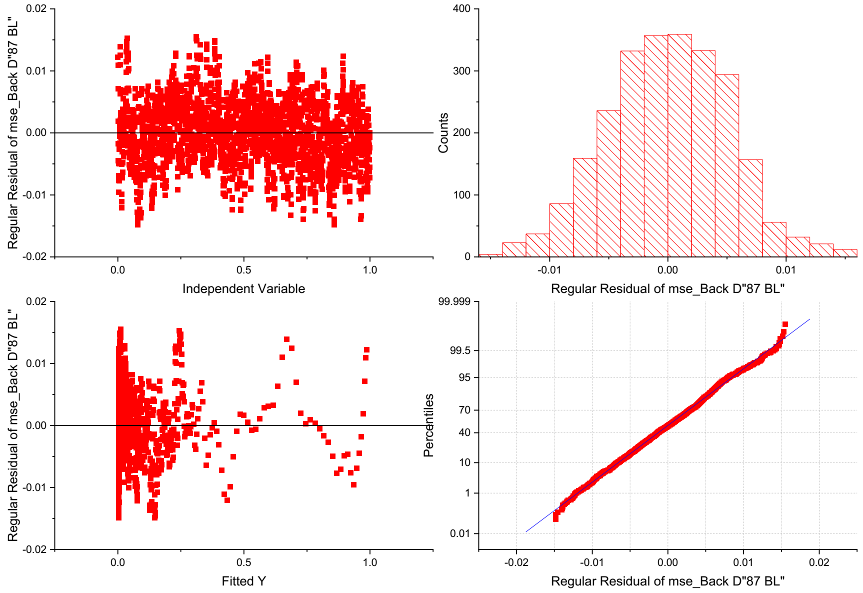

Residuals for 87 kDa PEI·FB Abragam + 2 exponencial Fit:

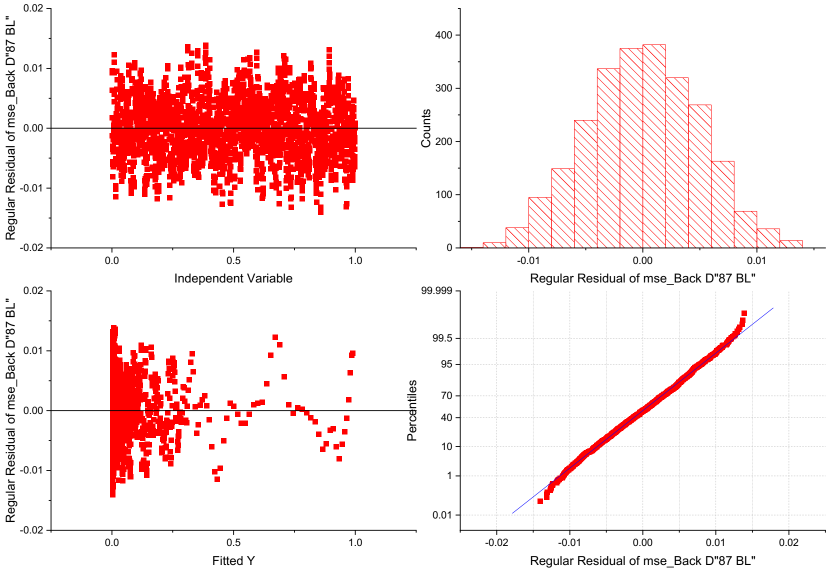

Regular Residual of mse_Back D"87 BL"
Independent Variable
Counts
Regular Residual of mse_Back D"87 BL"
Regular Residual of mse_Back D"87 BL"
Fitted Y
Percentiles
Regular Residual of mse_Back D"87 BL"

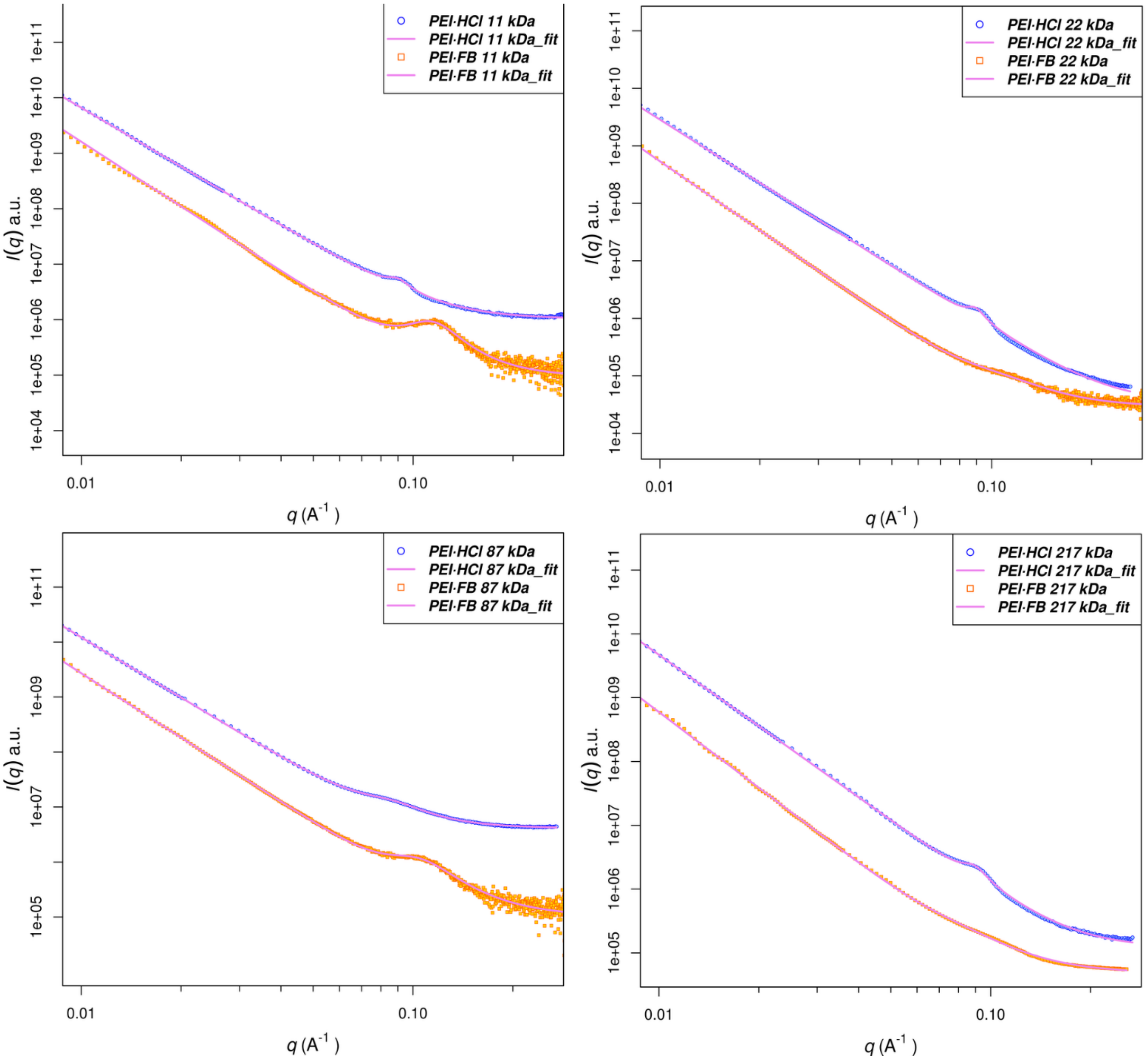

Figure S7. Fitting of SAXS results for PEI·FB and PEI·HCl samples.

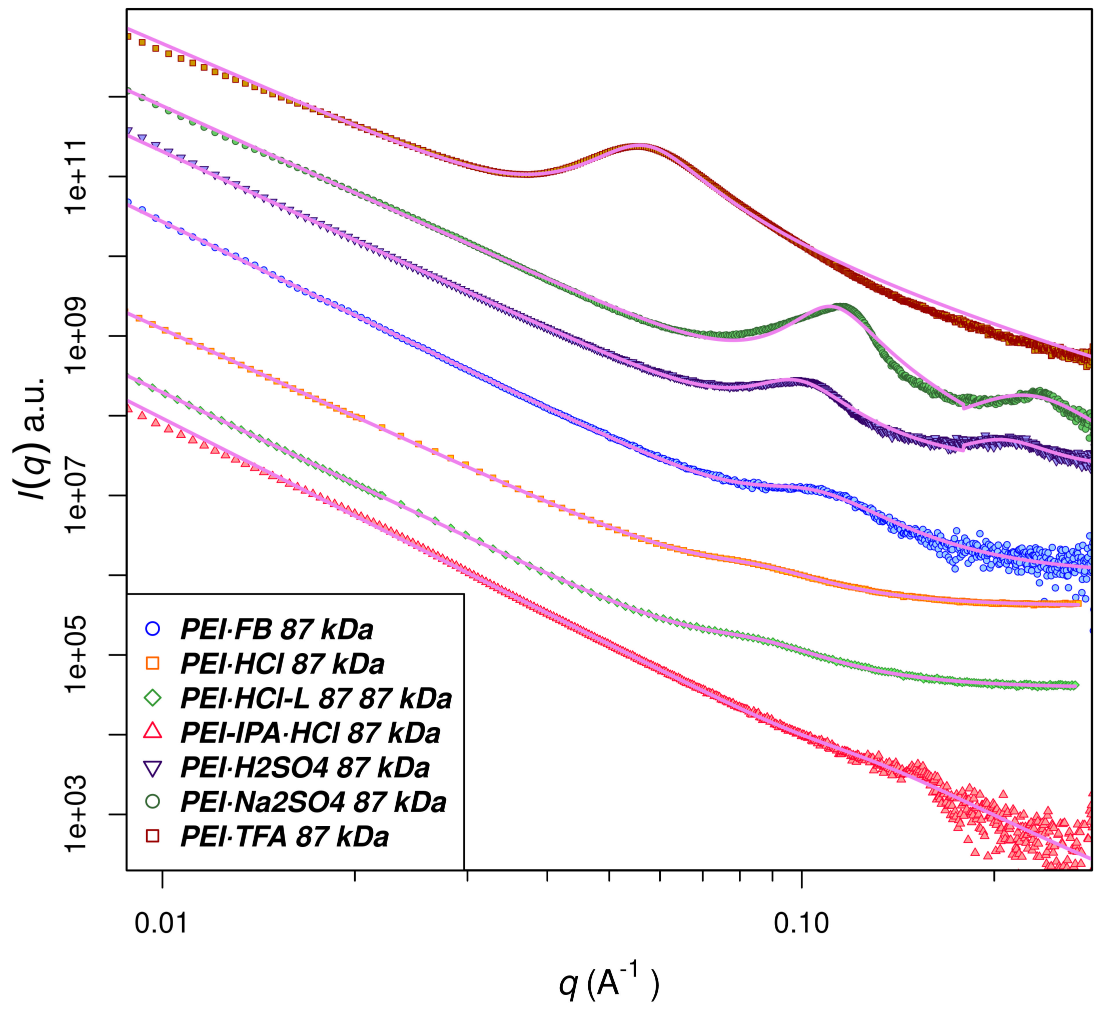

Figure S8. Fitting of SAXS results for the 87 kDa PEI materials.

Table S11. SAXS power-law. Lorentzian peak model parameters for PEI free bases and hydrochlorides.

Sample	*Scale*	Background (cm^{-1})	PL_{scale}	α	$Peak_{scale}$	Peak *q* ($Å^{-1}$)	Peak_HWHM ($Å^{-1}$)	χ^2
**PEI·FB 11 kDa**	0.13391 ± 2.5324	90466 ± 2388.8	211.86 ± 4006.3	3.8769 ± 0.0021942	5.2881×10^6 ± 1×10^8	0.11245 ± 0.00017881	0.023567 ± 0.0003308	12.226
**PEI·FB 22 kDa**	0.086187 ± 17.587	28599 ± 429.38	64.718 ± 13206	3.992 ± 0.0024155	4.9006×10^5 ± 1×10^8	0.092929 ± 0.00098573	0.049805 ± 0.0016567	1.9554
**PEI·FB 87 kDa**	0.19236 ± 4.6577	1.0048×10^5 ± 2535.3	230.26 ± 5575.3	3.8933 ± 0.0019078	4.13×10^6 ± 1×10^8	0.10031 ± 0.00021162	0.028165 ± 0.0003806	2.6112
**PEI·FB 217 kDa**	0.10876 ± 13.299	48134 ± 175.73	70.87 ± 8665.9	3.9439 ± 0.00099761	8.1781×10^5 ± 1×10^8	0.067268 ± 0.0005131	0.04069 ± 0.00045877	79.837
**PEI·HCl 11 kDa**	1.7611 ± 185.38	1.0405×10^6 ± 3273.5	335.98 ± 35366	3.5234 ± 0.0008815	9.5001×10^5 ± 1×10^8	0.089412 ± 0.00013871	0.0082536 ± 0.000211	17.105
**PEI·HCl 22 kDa**	0.43976 ± 36.059	33474 ± 185.87	329.82 ± 27045	3.6471 ± 0.00057415	1.2196×10^6 ± 1×10^8	0.089048 ± 3.3869×10^{-5}	0.0064193 ± 5.0945×10^{-5}	157.64
**PEI·HCl 87 kDa**	2.0338 ± 88.698	4.1014×10^6 ± 10685	307.3 ± 13402	3.645 ± 0.0018395	2.293×10^6 ± 1×10^8	0.082367 ± 0.00029834	0.025487 ± 0.00042123	1.6831
**PEI·HCl 217 kDa**	0.51031 ± 30.2	1.2042×10^5 ± 667.12	347.17 ± 20546	3.7115 ± 0.00080217	1.6897×10^6 ± 1×10^8	0.088511 ± 6.7496×10^{-5}	0.0091764 ± 0.00010218	16.624

Table S12. SAXS power-law. Lorentzian peak model parameters of 87 kDa PEI samples.

Sample	*Normscale*	Background (cm^{-1})	PLscale	α	Peakscale	Peak *q* ($Å^{-1}$)	Peak_HWHM ($Å^{-1}$)	χ^2
**PEI·H_2SO_4**	712.78 ± 2.37×10^5	2.2141×10^7 ± 3.60×10^5	11.763 ± 3916.2	3.6949 ± 0.0020	3.0036×10^5 ± 1×10^8	0.097002 ± 4.88×10^{-5}	0.01767 ± 9.95×10^{-5}	9.092
**PEI·Na_2SO_4**	4791.1 ± 1.04×10^6	0 ± 1.49×10^6	9.4499 ± 2041.5	3.6141 ± 0.0016	4.6286×10^5 ± 9.9995×10^7	0.11081 ± 1.66×10^{-5}	0.016011 ± 3.41×10^{-5}	120.76
**PEI·FB 87**	189.26 ± 4.51×10^5	1.0048×10^6 ± 2.54×10^4	2.3403 ± 5575.2	3.8933 ± 0.0019	4.1976×10^4 ± 9.9999×10^7	0.10031 ± 0.00021	0.028164 ± 0.00038	2.6112
**PEI-IPA·HCl**	0.95604 ± 45927	0 ± 42.687	0.74213 ± 35651	4.0578 ± 0.0015	2081.7 ± 1×10^8	0.11224 ± 0.00136	0.050442 ± 0.00295	10.665
**PEI-L·HCl**	2 ± 6033.3	38450 ± 183.65	2.3525 ± 7096.6	3.8095 ± 0.0016	33149 ± 1×10^8	0.081324 ± 0.00034	0.026447 ± 0.00050	1.1702
**PEI·HCl**	17.362 ± 64642	4.1013×10^5 ± 1068.5	3.5996 ± 13402	3.645 ± 0.0018	26859 ± 1×10^8	0.082367 ± 0.00030	0.025487 ± 0.00042	1.6831
**PEI·TFA 87**	3.8231×10^5 ± 5.30×10^7	0 ± 2.58×10^6	1.4956 ± 207.25	3.4539 ± 0.0021	6.1206×10^5 ± 8.48×10^7	0.055929 ± 3.92×10^{-6}	0.010626 ± 4.53×10^{-6}	190.41

SAXS Fitting Errors (Residual weights) (Figure S9).

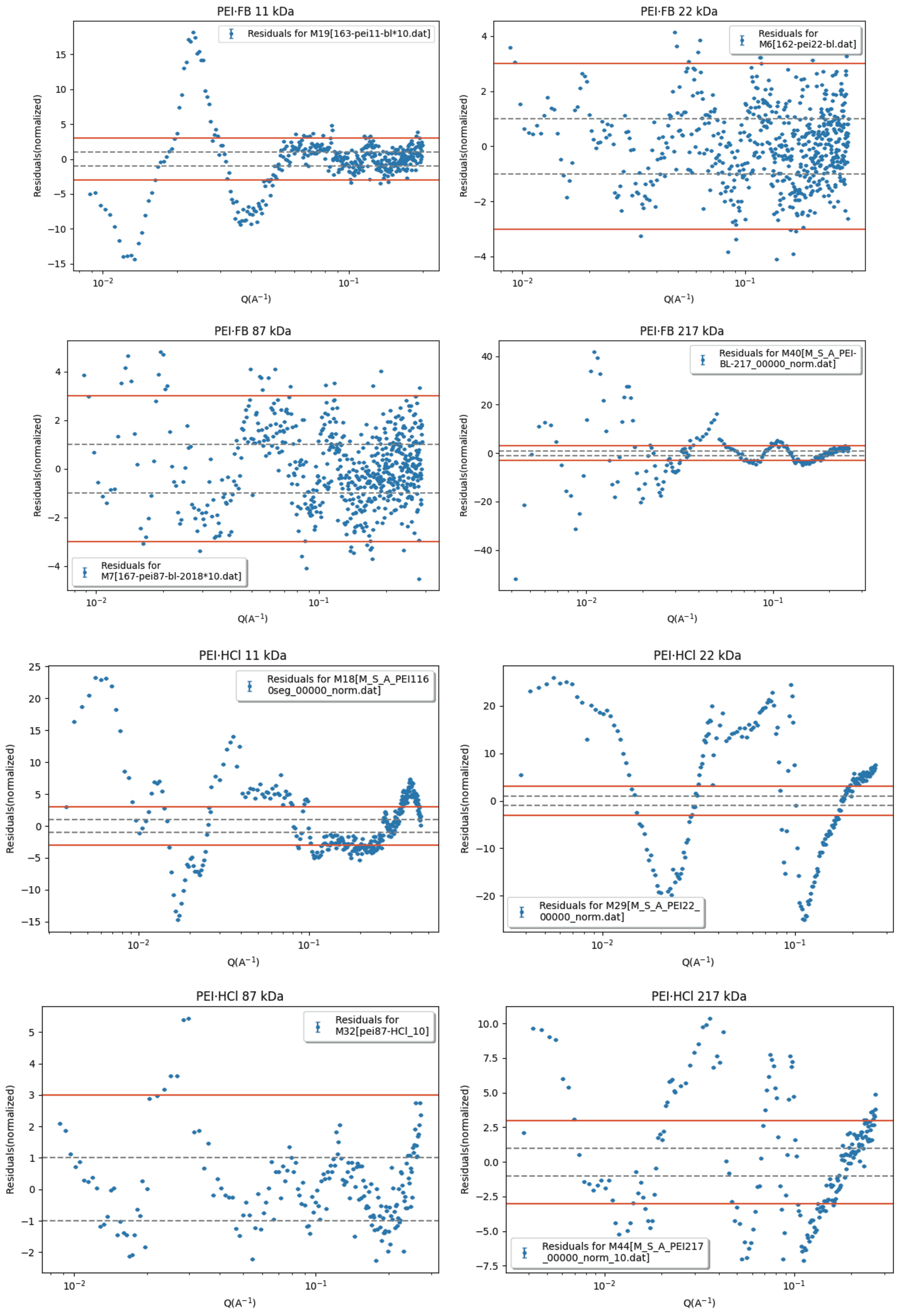

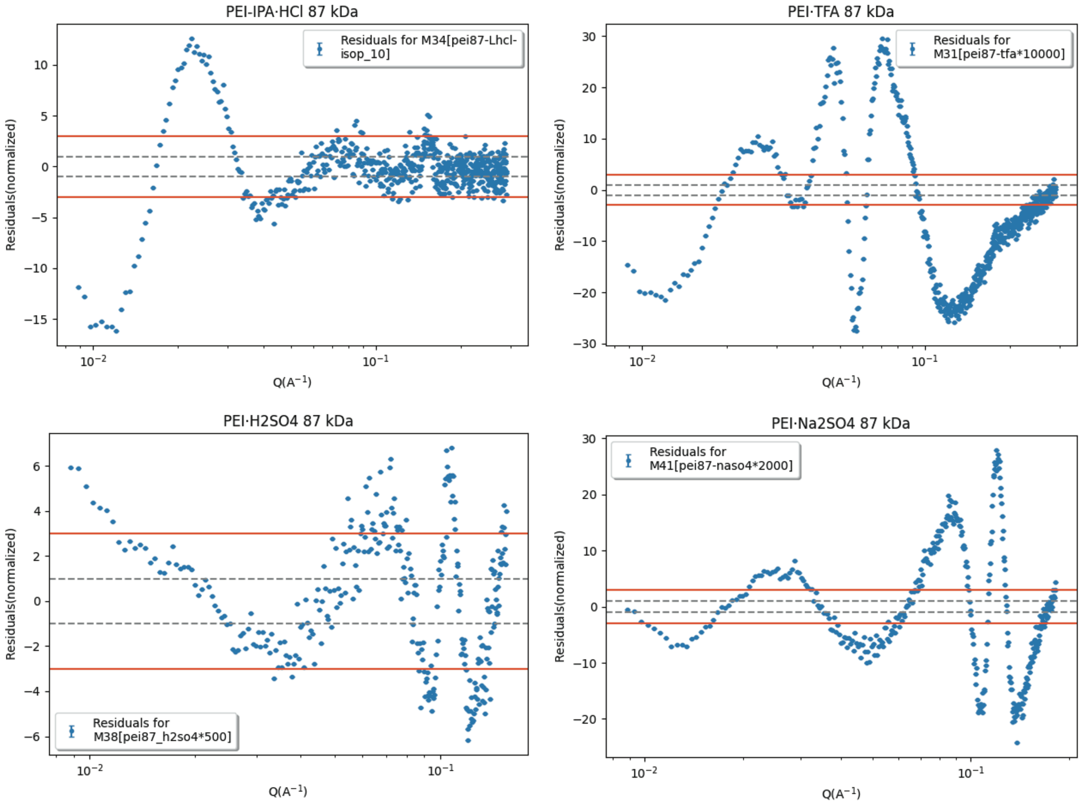
PEI·IPA·HCl 87 kDa
Residuals for M34[pei87-Lhcl-isop_10]
PEI·TFA 87 kDa
Residuals for M31[pei87-tfa*10000]
PEI·H2SO4 87 kDa
Residuals for M38[pei87_h2so4*500]
PEI·Na2SO4 87 kDa
Residuals for M41[pei87-naso4*2000]
Residuals(normalized)
Q(A^{-1})

-Synthetic Procedures (SP)-

Synthesis of PEI free bases (PEI·FB)

The PEI·FB materials were prepared from their corresponding PEI·HCl salts. Briefly, distilled water was previously heated to remove dissolved CO_2 and allowed to cool to room temperature. PEI·HCl was then dissolved under magnetic stirring (200 rpm), yielding an acidic solution with an initial pH of approximately 3. Subsequently, 2 N NaOH was added dropwise under continuous stirring until the formation of a white precipitate corresponding to PEI·FB was observed. The pH of the suspension was then measured and adjusted to approximately 11. The precipitated polymer was collected by vacuum filtration and extensively washed with distilled water until a neutral pH was reached. Finally, the different solids were left in a desiccator for 24 h (using anhydrous $CaCl_2$ as desiccant) and then were fractionated to carry out the different spectroscopic studies within 4 hours. The following table summarizes the mass of PEI·HCl used and the corresponding mass of PEI·FB obtained for each synthesis:

PEI MW (kDa)	PEI·HCl (g)	PEI·FB (g)
11	1.1	0.9
22	1.3	1.2
87	2.1	1.4
217	2.0	1.4

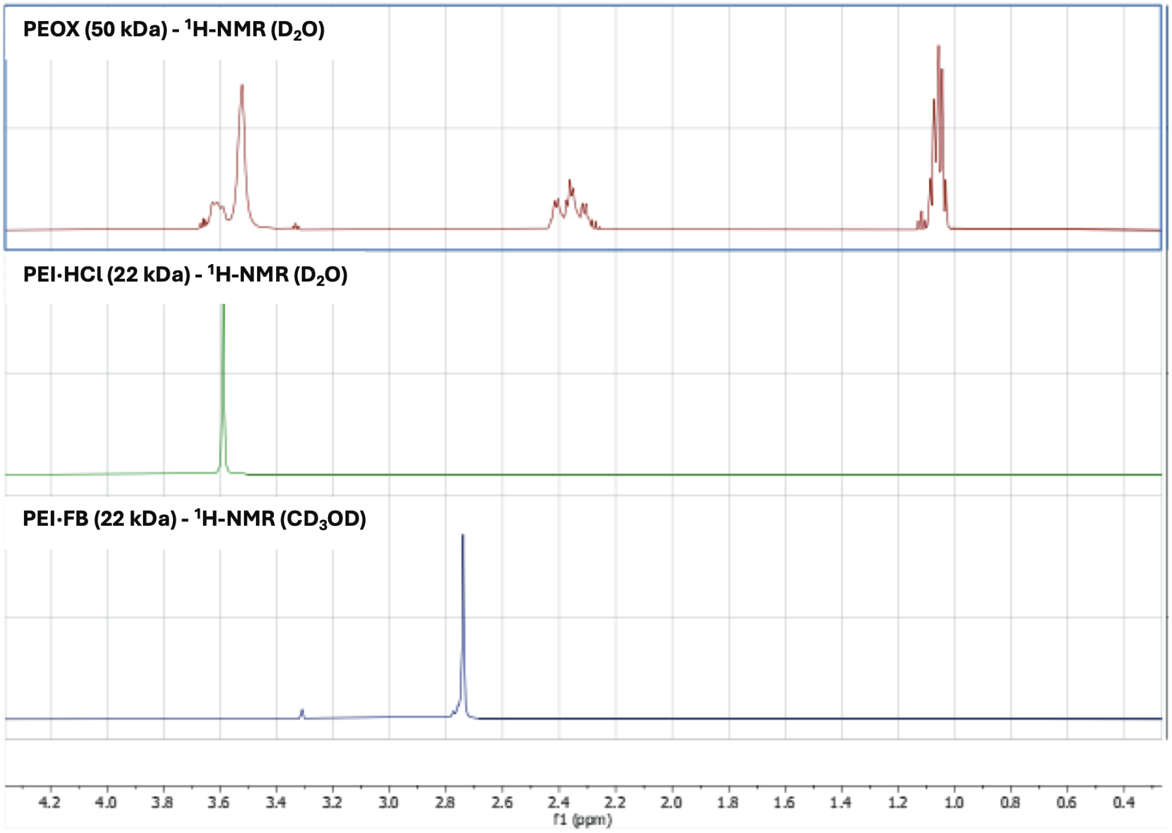

Figure S10. *Solution-state* ^{1}H-NMR spectra (600.09 MHz) of PEOX 50 kDa (D_2O), PEI·HCl 22 kDa (D_2O) and PEI·FB 22 kDa (CD_3OD).

Synthesis of PEI trifluoroacetate (PEI·TFA)

PEI·TFA sample was prepared by acidification of 87 kDa PEI·FB with trifluoroacetic acid (TFA). Briefly, 100 mg of PEI·FB were dissolved in 2 mL of anhydrous methanol. After complete dissolution, 2 mL of TFA were added dropwise under stirring (200 rpm), leading to the formation of a precipitate. The precipitate was collected by vacuum filtration, washed three times with 10 mL of anhydrous methanol, and dried to constant weight. The resulting material (174 mg) was stored at room temperature until further use.

Table S13. Organic elemental analysis in PEI·TFA sample.

Sample	Organic elemental analysis ($wt_{\%}$)				F:N	TFA:N	Monomer:TFA
	C	H	N	F[(a)]			
PEI·TFA	30.1	6.0	7.8	30.8	2.91	0.97	1:0.97

[(a)]The F content was obtained from the ^{19}F-NMR spectrum with the addition of NaF (as internal standard) to a solution of PEI·TFA sample in D_2O (Figure SF2).

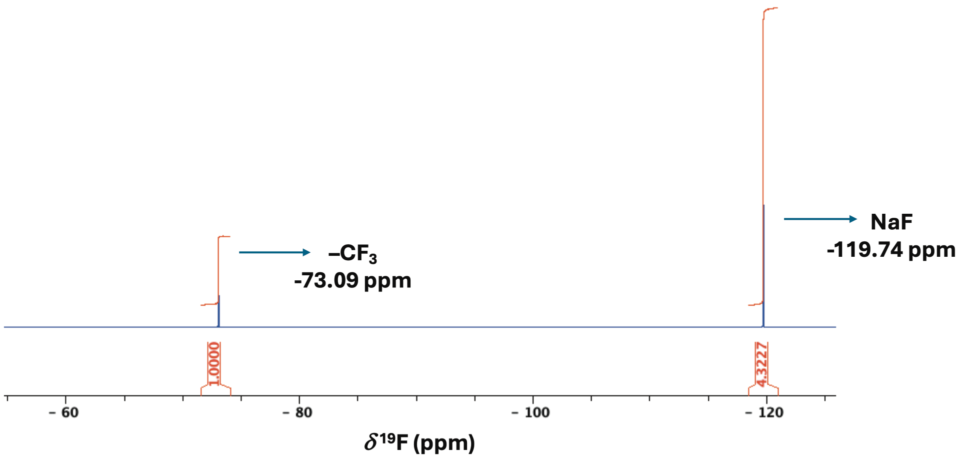

Figure S11. *Solution-state* ^{19}F-NMR (564.69 MHz) of PEI·TFA sample with the addition of NaF in D_2O.

Synthesis of PEI·HCl from PEI·FB (crystallized PEI·HCl)

PEI·HCl sample was prepared by acidification of 87 kDa PEI·FB with hydrogen chloride (1.25 M) in isopropanol (IPA·HCl). Briefly, 100 mg of PEI·FB were dissolved in 2 mL of anhydrous methanol. After complete dissolution, 2 mL of IPA·HCl solution were added under stirring (200 rpm), resulting in the formation of a white precipitate corresponding to the protonated polymer. The precipitate was collected by vacuum filtration, dried at room temperature to constant weight (rendering 96 mg), and stored at room temperature until further use.

Synthesis of PEI-IPA·HCl from PEI·FB

PEI-IPA·HCl sample was prepared by acidification of a solid sample of PEI·FB using IPA·HCl. Briefly, 100 mg of PEI·FB were dispersed in 2 mL of IPA·HCl solution (1.25 M

hydrogen chloride solution) under continuous stirring for 12 h (200 rpm) until complete evaporation of the liquid phase, rendering 69 mg of PEI-IPA·HCl.

Synthesis of PEI·Na_2SO_4 and PEI·H_2SO_4

PEI·Na_2SO_4 and PEI·H_2SO_4 samples were prepared from aqueous PEI·HCl solutions by adding equal amounts of sulphate supplied as sodium sulphate or sulfuric acid, respectively. Briefly, 300 mg of PEI·HCl was dissolved in 7.5 mL of distilled water under continuous stirring. Subsequently, 1.5 mL of either a 10% (w/v) Na_2SO_4 solution or a 6.90% (w/v) H_2SO_4 solution was added. Both solutions provided an equivalent amount of sulphate (1.06 mmol). In both cases, addition of the sulphate-containing solution resulted in the formation of a white precipitate. The resulting suspension was stirred (200 rpm) overnight at room temperature. The precipitate was then collected by centrifugation (2500 × *g*), washed three times with 5 mL of distilled water, and centrifuged again. Finally, the recovered solid was dried at room temperature to constant weight and stored at room temperature until further use, rendering 170 and 161 mg for the PEI·Na_2SO_4 and PEI·H_2SO_4 samples, respectively.

The organic elemental analysis and the content of sulphate in each material are show in Table S14.

Table S14. Organic elemental analysis in PEI·Sulphate samples.

Sample	Organic elemental analysis (OEA) ($wt_{\%}$)				Sulphate content ($wt_{\%}$)[(a)]	Monomer: Sulphate	Sodium content ($wt_{\%}$)[(b)]	Chlorine content ($wt_{\%}$)[(b)]
	C	H	N	S				
PEI·H_2SO_4	25.9	8.9	14.8	16.5	49.4	1:0.49	<0.01	<0.01
PEI·Na_2SO_4	27.1	9.3	15.3	17.2	51.5	1:0.49	<0.01	<0.01

[(a)]obtained from the OEA.
[(b)]obtained from the XPS analysis

Based on PEI·HCl repeat-unit basis (79.53 g mol^{-1}), the initial 300 mg corresponds to 3.77 mmol of repeat units. Each sulphate source supplies approximately 1.06 mmol, giving a ratio of approximately 0.280 mol SO_4^{2-} per mol of repeat units, sufficient to balance at most ~56% of the protonated nitrogen atoms. This ratio must be distinguished from the composition of the isolated solids where the S:N ratios are 0.491 for PEI·Na_2SO_4 and 0.487 for PEI·H_2SO_4 (Table S14), corresponding to near-stoichiometric charge compensation and an approximately 1.75-fold enrichment relative to the sulphate added. Combining the recovered masses with the measured nitrogen contents (15.3 and 14.8 wt%, respectively) gives repeat-unit recoveries of 49.2 and 45.1%. For the measured S:N ratios, conservation of the supplied sulphate sets maximum repeat-unit recoveries of approximately 57.0% and 57.4%, respectively.

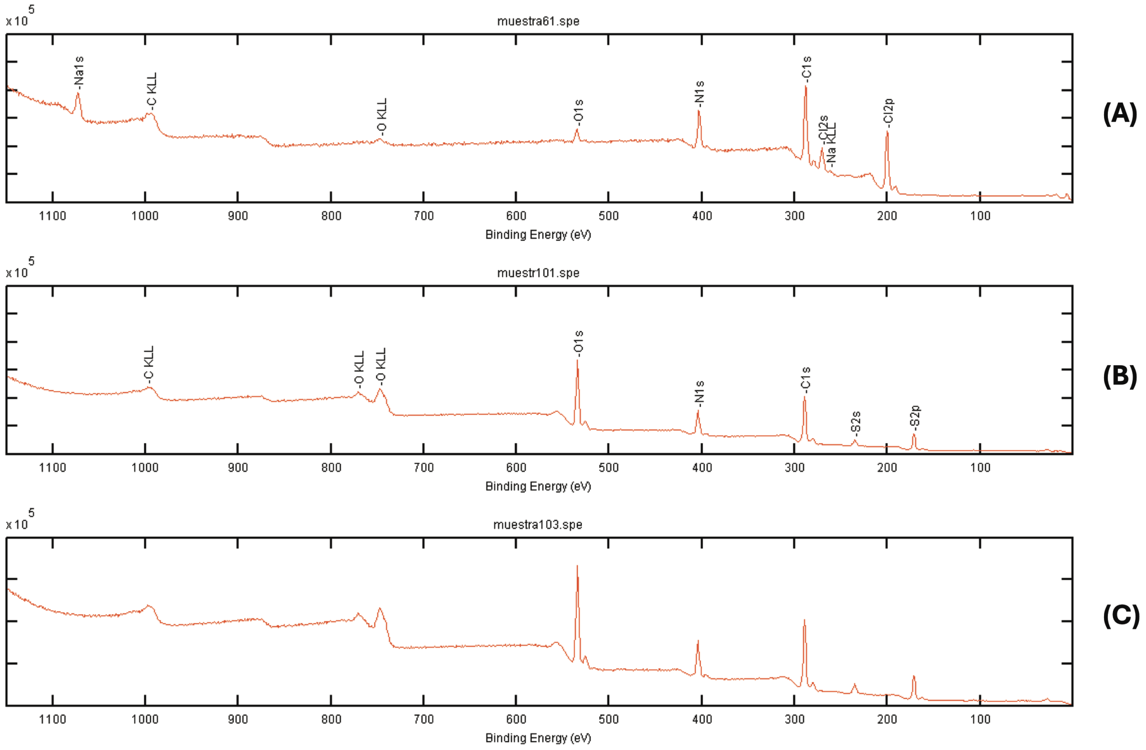

Figure S12. Panoramic XPS spectra for the PEI·HCl (A), PEI·Na_2SO_4 (B) and PEI·H_2SO_4 (C) samples.

Synthesis of deuterated PEI·HCl

The 87 kDa PEI·HCl was dissolved in deuterium oxide (100 mg in 5 mL) and kept under stirring overnight at 200 rpm. Subsequently, it was freeze-dried for three days. The degree of exchange was considered quantitative considering disappearance of the NH signal by ^{1}H-MAS *ss*-NMR spectrum (Figure SD1).

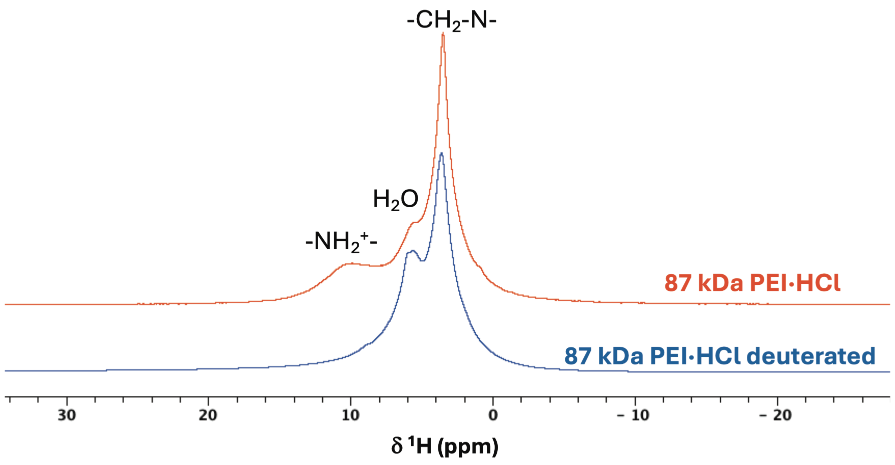

Figure S13. ^{1}H-MAS solid-state NMR spectra (MAS rate = 60 kHz, 499.70 MHz) for the 87 kDa PEI·HCl and deuterated 87 kDa PEI·HCl samples.

Synthesis of lyophilized 87 kDa PEI·HCl

The 87 kDa PEI·HCl was dissolved in distilled water (100 mg in 5 mL) following by a freeze-dried process for three days. The resulting solid material was stored in a desiccator until further use.

-Characterization Techniques (CT)-

For the different characterization techniques, samples were ground in an agate mortar.

General characterization techniques

ATR-FTIR spectra were recorded on a Nicolet iS50 spectrometer (Thermo Scientific-Waltham, MA, USA). The XPS analysis was carried out with a Physical Electronics Versa-Pro II (Chanhassen, MI, USA) operating with a monochromatic X-ray source Al (Ka) of photons at 1486 eV under ultra-high vacuum (10^{-6} Pa).

The organic elemental analysis was performed in a CE440 Elemental Analyser device (Coventry, UK). It is important to remark that the hydrogen contents exceed the values expected from the measured nitrogen contents by a similar amount in the PEI·TFA, PEI·Na_2SO_4 and PEI·H_2SO_4 samples (around 2.5 percentage points). However, no empirical correction has been applied, and hydrogen was not used to determine hydration or protonation quantitatively.

In general, all experimental procedures and characterization techniques were performed at 24±1°C and relative humidity between 40-50%, unless otherwise specified.

X-ray powder diffraction (XRD) studies

The XRD patterns were recorded on an Empyrean diffractometer (Panalytical, The Netherlands), with a PixCel3D area detector in a 1D scanning lineal mode, over the 2θ range of 5−40°, using Cu Kα radiation (1.54184 Å) obtained from an X-ray generator set at 40 kV and 40 mA. Suitable samples for XRPD measurement were prepared in aluminum sample holders. Measurements were performed using a 0.5° divergence slit and 8 mm receiving slit, scanning step 0.0263°, counting time 200 s.

NMR studies

Carbon-13 and nitrogen-15 *solid-state* NMR spectra were acquired using a Bruker Avance-III HD spectrometer (Billerica, MA, USA) at Larmor frequencies of 150.91 and 60.80 MHz, respectively, in a 3.2 mm magic-angle spinning (MAS) probe using ZrO_2 rotors at a controlled rotor temperature of 30±0.1ºC.

High-resolution ^{13}C *solid-state* NMR spectra of all samples were acquired using a ramped cross-polarization magic-angle spinning (ramp-CP/MAS) pulse sequence. During the contact time, the ^{1}H radiofrequency amplitude was linearly ramped from 50% to 100% of its maximum value. The ^{1}H $\pi/2$ pulse length was 3.5 μs (Time-domain = 4096 and spectral width = 400 ppm). Heteronuclear decoupling during acquisition was performed with SPINAL-64 acquisition with a proton field satisfying $\omega^{1}H/2\pi=60$ kHz. The FIDs were apodized with an exponential line broadening of 10–20 Hz. The operating conditions in the acquisition of the ^{13}C CP-MAS experiments (cross-polarisation MAS) were a CP contact time of 2 ms, a recycle delay of 5 s, 4000 transients and spin rate of 10 kHz.

For ^{15}N CP-MAS experiments (Time-domain = 4096 and spectral width = 800 ppm), a CP contact time of 5 ms, a recycle delay of 5 s, 40,000-60,000 transients and spin rate of 10 kHz. It is important to remark that the ^{15}N NMR spectra were performed at natural

abundance (0.37%). The FIDs were apodized with an exponential line broadening of 10–20 Hz.

In both ^{13}C and ^{15}N CP-MAS experiments, the SPINAL-64 sequence for ^{1}H decoupling was applied during signal acquisition [4].

For *solution-state* NMR experiments the same spectrometer was used for the acquisition of ^{1}H, ^{13}C and ^{19}F at frequencies of 600.09, 150.91 and 564.69 MHz, respectively.

Proton MAS spectra were recorded using a Varian InfinityPlus spectrometer operating at a ^{1}H frequency of 499.70 MHz, and a Bruker MAS probe using 1.3 mm diameter zirconia rotors. The ^{1}H $\pi/2$ pulse length was 3.0 μs (Time-domain = 1024 and spectral width = 100 ppm). Spectra were typically acquired using a recycle delay of 5 s, 64 transients, spin rate of 60 kHz and controlled rotor temperature of 30±0.1ºC. The 2D Single Quantum/Double Quantum (SQ/DQ) experiments were recorded using the back-to-back (BaBa) pulse sequence with excitation and reconversion times of different rotor periods (1-4) at a MAS rate of 60 kHz [5].

Proton chemical shifts were referenced to the signal for tetramethylsilane. Carbon chemical shifts were referenced to the signal for tetramethylsilane via a replacement sample of solid adamantane (38.4 ppm for the high-frequency line). Nitrogen chemical shifts were referenced using ^{15}N-enriched glycine at –347.4 ppm relative to the signal of neat nitromethane.

The ^{1}H- and ^{13}C-HRMAS NMR experiments were recorded as described previously using 4 mm ZrO_2 HRMAS rotors with 50 μL spherical insert (3 mg of material and 40 μL of deuterated solvent). The sample was spun at a MAS rate of 4 kHz [6] .

Computational methodology

Density functional theory (DFT) calculations were performed using Gaussian 16 [7]. Geometry optimizations were carried out at the B3LYP/6-31G(d) level and repeated with the B3LYP-D3(BJ) and ωB97X-D functionals, using the same basis set, to evaluate the effect of dispersion corrections. The ^{13}C and ^{15}N magnetic shielding tensors were subsequently computed on the optimized geometries using the GIAO method at the corresponding B3LYP/6-311+G(2d,p), B3LYP-D3(BJ)/6-311+G(2d,p) and ωB97X-D/6-311+G(2d,p) basis set. Frequency analysis was performed to confirm that optimized geometries correspond to local minima on the potential energy surface. Initial geometries were constructed manually and subsequently optimized as described above.

L-PEI polymer was represented by hexamer models (six nitrogen atoms), in which the four internal secondary amines constitute the protonable sites, while the two terminal primary amines were kept unprotonated. Protonation degrees of 0, 25, 50 and 100% were modelled by protonating one, two or four of these internal nitrogen atoms, with the corresponding number of counterions (Cl^- or $CF_3CO_2^-$) included explicitly so that all systems remained neutral and closed-shell. To assess the influence of interchain packing, dimeric models consisting of two stacked hexamer chains were additionally optimized at 0 and 100% protonation with Cl^- as a counterion.

The chemical shifts values (δ) were reported relative to the reference compound, typically tetramethylsilane (TMS), for ^{13}C, and nitromethane for ^{15}N, according to:

$$\delta = \sigma(reference) - \sigma(X) \qquad \textit{(Equation (S1))}$$

Where the isotropic orbital shielding of the reference compound is computed using the same methodology. The two terminal repeat units of the hexamer are adjacent to unprotonated primary amines and therefore constitute a finite-size artifact of the model, with no counterpart in a polymer of high degree of polymerization. The analysis was consequently restricted to the six internal backbone carbons.

All models were treated as isolated molecular clusters in the gas phase; no periodic boundary conditions or explicit solvent were employed. These models are not intended to reproduce the crystalline structure or the bulk properties of the solid, but were deliberately constructed to isolate and rank the relative contributions of protonation degree and interchain packing to the ^{13}C chemical shift. Accordingly, the discussion is based on relative trends and differences between systematically constructed systems rather than on absolute chemical shifts values.

Time-domain NMR studies

The MSE experiments were performed on a Bruker Minispec mq20 spectrometer (Billerica, MA, USA) at a controlled sample temperature of 36.0±0.1°C, using a $\pi/2$ pulse length of 3.7 μs. The pulse sequence consists of an initial $\pi/2$ pulse followed by a delay *t* and a so-called "sandwich" part of duration 4t, which comprises two $\pi/2$ pulses of the same phase enclosing two blocks of four $\pi/2$ pulses each. The echo is subsequently generated after a final delay t. The obtained signal is then fitted using an Abragam function corresponding to the rigid domain, along with two exponential functions representing the interfacial and mobile phases, respectively [8].

$$S(t) = A_c exp^{(-0.5(at)^2)} \frac{sin(bt)}{bt} + A_i exp^{-(\frac{t}{T_{2i}})} + A_a exp^{-(\frac{t}{T_{2a}})} \qquad \textit{(Equation (S2))}$$

Where A_c, A_i, and A_a are the coefficients that correspond to the crystalline (rigid), interfacial and amorphous (mobile) phases. T_{2i} and T_{2a} are the spin – spin relaxation times of the interfacial and amorphous phases. The MSE decays were fitted over the range t = 0–1 ms (n = 2499 points per curve). In addition to the parameters in Equation 2, a constant baseline offset (y0) was included in the fit. The three-component model (Abragam function + two exponentials, k = 8 free parameters) was compared against a reduced two-component model (Abragam function + one exponential, k = 6) using the reduced χ^2, an extra-sum-of-squares F-test, and the corrected Akaike Information Criterion (AICc). The three-component model was statistically favored for all materials except PEI·Na_2SO_4 and PEI·H_2SO_4, for which the three-component fit was not identifiable ($T_{2i} \approx T_{2a}$ within error) and the two-component model was retained; conversely, for 87 kDa PEI·HCl the two-component model required fixing *w* to achieve convergence, further supporting the three-component description for that material.

Small Angle X-ray Scattering studies

The investigation of the ultrastructure of the PEI materials was performed by Small Angle X-ray Scattering using the SAXS1 beamline of the National Synchrotron Light Laboratory (LNLS), Campinas, Brazil. The SAXS measurements were performed at room

temperature in transmission geometry with λ = 1.55 Å (8 keV). The 2D SAXS spectra were monitored using a Pilatus 300 K detector. A sample to-detector distance of 0.9 and 3 m. Radiation damage was evaluated by exposing samples to increasing exposures up to 120 s. For all measurements an exposure time of 60 s were used. Calibration (silver behenate) and beam set-up was performed by beamline officers. The samples were placed within Kapton tape with their surfaces perpendicular to the direction of the incident X-ray beam and parallel to the X-ray detector. The scattering intensity (I) was measured as a function of the scattering vector (q) from 0.01 to 0.26 $Å^{-1}$. The background and parasitic scattering were determined by using an empty sample holder and were subtracted for each measurement. Scattering profiles modeling was performed using SasView 6.1.3 software [9]. For the comparison of PEI samples, a plugin model was generated by the combination of two preexisting models: 1) power_law; 2) peak_lorentz. For the analysis of SAXS profiles, by combining a power-law model and a Lorentzian peak model, a plugin model was generated. For the low-to-mid q region, a power law, described by equation 3, accounted for the scattering of surface fractal like behavior of agglomerated polymer particles. For the high q region function, a peak or bump, described by equation 4, accounted to a Lorentzian correlation peak. Samples which showed a second correlation peak were modelled with equation 4 for their second peak.

$$I(q) = scale \cdot q^{-\alpha} + background \qquad \textit{(Equation (S3))}$$

$$I(q) = \frac{I_0}{\left(1+\left(\frac{q-q_0}{B}\right)^2\right)} + background \qquad \textit{(Equation (S4))}$$

with the peak having height of I_0 centered at q_0 and having a HWHM (half-width half-maximum) of B. *Scale* represents scale factors.

Swelling studies

The swelling behavior of the different materials was studied by the gravimetric method in 10 mM phosphate buffer solution (at pH = 4.0, 6.0, and 8.0) at 24±1°C in triplicate. In each experiment, the dried material was immersed in water for 24 h. Then, the swelled sample was separated from the solution and weighed, after previous surface water removal with filter paper. Finally, the swelled samples were oven-dried at 60ºC until constant weight was obtained. The swelling capacity (S) was calculated using the following equation 5:

$$S(\%) = \frac{Ms - Md}{Md} \times 100 \qquad \textit{(Equation (S5))}$$

where *Ms* and *Md* are the swelled and dried sample weight, respectively.

References:

1. Chatani, Y.; Tadokoro, H.; Saegusa, T.; Ikeda, H. Structural Studies of Poly(Ethylenimine). 1. Structures of Two Hydrates of Poly(Ethylenimine): Sesquihydrate and Dihydrate. *Macromolecules* **1981**, *14*, 315–321, doi:10.1021/ma50003a017.

2. Chatani, Y.; Kobatake, T.; Tadokoro, H.; Tanaka, R. Structural Studies of Poly(Ethylenimine). 2. Double-Stranded Helical Chains in the Anhydrate. *Macromolecules* **1982**, *15*, 170–176, doi:10.1021/ma00229a034.
3. Chatani, Y.; Kobatake, T.; Tadokoro, H. Structural Studies of Poly(Ethylenimine). 3. Structural Characterization of Anhydrous and Hydrous States and Crystal Structure of the Hemihydrate. *Macromolecules* **1983**, *16*, 199–204, doi:10.1021/ma00236a009.
4. Fung, B.M.; Khitrin, A.K.; Ermolaev, K. An Improved Broadband Decoupling Sequence for Liquid Crystals and Solids. *Journal of Magnetic Resonance* **2000**, *142*, 97–101, doi:10.1006/jmre.1999.1896.
5. Feike, M.; Demco, D.E.; Graf, R.; Gottwald, J.; Hafner, S.; Spiess, H.W. Broadband Multiple-Quantum NMR Spectroscopy. *J. Magn. Reson. A* **1996**, *122*, 214–221, doi:10.1006/jmra.1996.0197.
6. Araque, L.M.; Pérez, C.J.; Infantes-Molina, A.; Rodríguez-Castellón, E.; Copello, G.J.; Lázaro-Martínez, J.M. Linear <scp>PEI</Scp> -based Responsive Hydrogels: Synthesis and Characterization. *J. Appl. Polym. Sci.* **2023**, *140*, doi:10.1002/app.54057.
7. Frisch, M.J.; Trucks, G.W.; Schlegel, H.B.; Scuseria, G.E.; Robb, M.A.; Cheeseman, J.R.; Scalmani, G.; Barone, V.; Petersson, G.A.; Nakatsuji, H.; et al. Gaussian 16, Rev. C.01. *Gaussian 16, Rev. C. 01* **2016**.
8. Abragam, A.; Carr, H.Y. *The Principles of Nuclear Magnetism*. *Phys. Today* **1961**, *14*, 56–58, doi:10.1063/1.3057238.
9. J. Krzywon et al. SasView Version 6.1.3 10.5281/Zenodo.18352296 Available online: 10.5281/zenodo.18352296 (accessed on 8 July 2026).
